# Contributions of 2‐h post‐load glucose, fasting blood glucose and glycosylated haemoglobin elevations to the prevalence of diabetes and pre‐diabetes in adults: A systematic analysis of global data

**DOI:** 10.1111/dom.70130

**Published:** 2025-09-15

**Authors:** Xue Xue, Jiaxuan Li, Wenxiao Zheng, Bingrui Zhang, Shuting Wang, Jiayue Zhang, Zuyao Yang

**Affiliations:** ^1^ Department of Nephrology and Hubei Shizhen Laboratory Affiliated Hospital of Hubei University of Chinese Medicine, Hubei Provincial Hospital of Traditional Chinese Medicine Wuhan Hubei China; ^2^ The Jockey Club School of Public Health and Primary Care The Chinese University of Hong Kong Hong Kong Special Administrative Region China; ^3^ Dongzhimen Hospital Beijing University of Chinese Medicine Beijing China; ^4^ Faculty of Medicine Macau University of Science and Technology Macao Special Administrative Region China

**Keywords:** 2‐h post‐load glucose, diabetes, fasting blood glucose, glycosylated haemoglobin, pre‐diabetes, prevalence, systematic review

## Abstract

**Aims:**

Some studies found that the association of fasting blood glucose (FPG) or glycosylated haemoglobin (HbA1c) elevation with diabetic complications was not statistically significant after controlling for the confounding caused by 2‐h post‐load glucose (2hPG) elevation. Furthermore, inclusion of HbA1c as a diagnostic measure for diabetes has raised some concerns about over‐diagnosis and over‐treatment. This study aimed to quantify the contributions of 2hPG, FPG and HbA1c elevations to the prevalence of diabetes and pre‐diabetes respectively, by synthesising global data, which can serve as essential parameters in cost‐effectiveness analysis and inform updates of practice guidelines about prevention and control of diabetes.

**Materials and Methods:**

The levels of 2hPG, FPG and HbA1c were classified as either ‘elevated’ or ‘normal.’ Each distinct combination of these markers (e.g., ‘elevated 2hPG, normal FPG and normal HbA1c’) defined a unique subgroup, resulting in a total of seven subgroups for both diabetes and pre‐diabetes. The contribution of 2hPG elevation to diabetes prevalence was calculated as its proportion among all diabetes cases; the same approach was applied to FPG and HbA1c elevations, as well as to pre‐diabetes prevalence. To retrieve global data, five electronic databases (i.e., PubMed, EMBASE, Web of Science, China National Knowledge Infrastructure and Wan Fang) were searched from their inception to February 2024. Studies that fulfilled the following criteria were considered eligible: (1) were conducted in adults without previously diagnosed diabetes; (2) were cross‐sectional studies or baseline surveys of cohort studies (which can be regarded as a special type of cross‐sectional studies); and (3) reported directly or allowed for calculation of the proportion of each subgroup out of all diabetes and/or the proportion of each subgroup out of all pre‐diabetes. A 10‐item tool selected from literature was used to appraise the quality of included data. The proportions of each subgroup among all diabetes cases were meta‐analysed across eligible studies with the random‐effects model using the MetaXL software. Similar meta‐analyses were conducted for pre‐diabetes.

**Results:**

Thirty‐two eligible studies were identified, with 25 reporting on newly detected diabetes (*n* = 289 094) and 15 on pre‐diabetes (*n* = 221 988) and the majority of them using identical diagnostic cutoffs proposed by the American Diabetes Association (ADA). The mean age of participants ranged from 24 to 68 years (median of mean ages: 51). Twenty‐four studies (75%) were assessed as at low risk for ≥7 out of the 10 quality items. In the general population, based on the ADA criteria, the weighted prevalence of diabetes and pre‐diabetes was 15% and 69%, respectively. Among those with newly detected diabetes (*n* = 24 214), 69% had elevated 2hPG, 44% elevated FPG and 61% elevated HbA1c; 7% had isolated FPG elevation; 20% had isolated HbA1c elevation. Among those with newly detected pre‐diabetes (*n* = 133 621), 33% had elevated 2hPG, 51% elevated FPG and 68% elevated HbA1c; 17% had isolated FPG elevation; 34% had isolated HbA1c elevation. Sensitivity analyses stratified by participant comorbidities and study quality produced results consistent with the main findings.

**Conclusions:**

The largest contributors to the prevalence of diabetes and pre‐diabetes are 2hPG and HbA1c, respectively. Isolated FPG and HbA1c elevations account for over a quarter of all diabetes and more than half of all pre‐diabetes.

## 1 INTRODUCTION

Diabetes is a growing global public health concern, affecting over 537 million individuals worldwide—over 90% of whom have type 2 diabetes.^1^ Even more people are affected by pre‐diabetes, an intermediate stage between normoglycaemia and diabetes, although the introduction of this term has caused some concerns about over‐diagnosis and over‐treatment.^2^ Detection of diabetes and pre‐diabetes aims to identify individuals at increased risk of macrovascular (e.g., cardiovascular disease) and microvascular complications (e.g., retinopathy) and inform subsequent interventions, including pharmacological treatment. The diagnostic criteria for diabetes have been revised several times over the past few decades.^3^, ^4^, ^5^, ^6^, ^7^ Two‐hour post‐load glucose (2hPG) was first used to diagnose diabetes,^3^ and then fasting blood glucose (FPG) and glycosylated haemoglobin (HbA1c) were added to the diagnostic criteria.^3^, ^6^ Likewise, the criteria for diagnosing pre‐diabetes include impaired glucose tolerance (IGT), impaired fasting glucose (IFG) and elevated HbA1c.^8^ According to the latest guidelines of the American Diabetes Association (ADA), a diagnosis of diabetes or pre‐diabetes can be made when any of these glycaemic indicators exceed their respective thresholds.^7^ Although diagnostic practice in other countries or populations may vary in terms of the more commonly used diagnostic measures (e.g., whether HbA1c testing is preferred), the cutoffs adopted (e.g., the lower thresholds of FPG and HbA1c for diagnosing pre‐diabetes) and diagnostic rigour (e.g., whether repeat testing is required if the first testing is positive), most countries' guidelines recommend that 2hPG, FPG and HbA1c can all be used for the diagnosis of diabetes and pre‐diabetes.^9^, ^10^, ^11^, ^12^ The increase in the number of diagnostic measures would inevitably increase the number of new cases and prevalence of diabetes and pre‐diabetes, because the test results of different diagnostic measures do not align with each other perfectly. For example, in the case of isolated FPG elevation, the FPG level exceeds the diagnostic threshold for diabetes, while 2hPG and HbA1c are ‘normal.’

More importantly, not all of the newly identified patients are at increased risk of diabetic complications. For example, previous studies consistently found that 2hPG was independently associated with increased risk of cardiovascular disease after adjustment for FPG, HbA1c and non‐glycaemic risk factors,^13^, ^14^, ^15^ whereas FPG was often not associated with risk of cardiovascular disease after adjusting for 2hPG and HbA1c.^13^, ^14^, ^16^ These findings suggest that some patients (e.g., those with isolated FPG elevation) may derive little benefit from glucose‐lowering drug treatment in terms of preventing diabetic complications as their baseline risk is low in the first place. Thus, it would be of interest to know the contribution of each glycaemic measure to the prevalence of diabetes and pre‐diabetes, for instance, the proportions of ‘individuals with isolated FPG elevation’ and ‘individuals with abnormal values for two or three diagnostic measures’ (who are at different risks of complications), out of all patients detected by the three glycaemic measures. This information can serve as essential parameters in cost‐effective analysis and development of clinical guidelines about diabetes management.

Against this background, we conducted a systematic review to quantify the contributions of 2hPG, FPG and HbA1c to the prevalence of diabetes and pre‐diabetes, respectively, and to examine how the patients identified by one glycaemic measure overlap with those identified by other measures.

## 2 METHODS

### 2.1 Protocol and registration

The protocol of this systematic review was registered on International Platform of Registered Systematic Review and Meta‐analysis Protocols (INPLASY.COM) (Registration Number: 202490045). The review was reported in accordance with Preferred Reporting Items for Systematic Reviews and Meta‐Analyses (PRISMA‐2020).^17^

### 2.2 Eligibility criteria

Cross‐sectional studies and the baseline surveys of cohort studies (which can be regarded a special type of cross‐sectional studies) conducted in adults without previously diagnosed diabetes and reported relevant outcome data were eligible for this systematic review. ‘Previously diagnosed diabetes’ was defined as having ever been diagnosed with diabetes or currently taking blood glucose‐lowering drugs as reported by participants. The outcomes of interest were the proportions of different subgroups defined by combinations of 2hPG, FPG and HbA1c levels among people with newly detected diabetes or pre‐diabetes. Taking people with diabetes as an example, there were seven possible subgroups: (1) 2hPG elevated alone; (2) FPG elevated alone; (3) HbA1c elevated alone; (4) both 2hPG and FPG elevated; (5) both 2hPG and HbA1c elevated; (6) both FPG and HbA1c elevated; (7) all the three measures elevated. Similarly, for people with pre‐diabetes, there were also seven subgroups. Eligible studies were required to have reported either the proportion of each subgroup directly or the data that can be used to calculate it (i.e., the number of participants in each subgroup and the total number of patients with newly detected diabetes or pre‐diabetes), but did not have to use the same criteria to define diabetes and pre‐diabetes (the studies using ADA criteria and those using other criteria were analysed separately; see Section 2.6). Also, eligible studies were not required to be explicitly restricted to individuals with type 2 diabetes, for two reasons. First, our pilot literature search showed that most potentially eligible studies did not differentiate type 2 diabetes from total diabetes, which indicated that there would be little data available for analysis if it were limited to type 2 diabetes. Second, as well documented, type 2 diabetes accounts for over 90% of all diabetes. Thus, it would be reasonable to consider our findings as primarily derived from and applicable to type 2 diabetes populations.

### 2.3 Search strategy

We conducted a systematic literature search in the following databases from their inception to 17 February 2024: PubMed, EMBASE, Web of Science, China National Knowledge Infrastructure (CNKI, in Chinese) and Wan Fang (in Chinese). Additionally, related reviews, conference papers, reference lists and grey literature were searched manually. No restrictions on language or publication status of studies were applied. The literature search strategies are described in detail in Table S1.

### 2.4 Study selection and data extraction

EndNote software (version 21) was used for study selection and deduplication. We firstly removed duplicate records from different databases using the deduplication function of EndNote and then manually screened the remaining unique records one by one, which was done by two reviewers (Xue Xue and Jiaxuan Li) in parallel independently to ensure accuracy. Any conflicts during study selection were resolved through discussion between the two reviewers, with consultation of a third expert (corresponding author) when necessary. We developed and piloted a standardised Excel form for data extraction. Reviewers were trained to use this standard form, extracted data from eligible papers independently, and then cross‐checked the extracted data against the original studies. Disagreements between the two were resolved through discussions with the corresponding author Zuyao Yang. For non‐English and non‐Chinese studies that were considered seemingly eligible according to their titles/abstracts, we planned to translate them using ChatGPT first and then verify the accuracy of the translated contents with the help of qualified bilingual professionals, but the plan was not actually implemented as no such studies were encountered during the screening process. After the eligible papers were identified, the following information was extracted from them: first author, publication year, study design, country/region, total sample size, the characteristics of participants (e.g., age, gender and pre‐existing diseases), number of newly identified patients with diabetes or pre‐diabetes, criteria for diagnosing diabetes or pre‐diabetes and number of participants in each of the seven subgroups mentioned above.

### 2.5 Methodological quality assessment

The risk of bias tool developed by Hoy et al.^18^ was adopted to appraise the methodological quality of the included studies by two authors (Xue Xue and Jiaxuan Li) independently. Any disagreements were resolved by discussion with the corresponding author. This methodological quality assessment tool for prevalence studies consists of 10 items addressing three types of bias, along with a summary risk of bias assessment. Items 1–4 assess external validity, focusing on selection bias arising from the sampling procedure and non‐response. Items 5–10 assess internal validity, with items 5–9 related to measurement bias and item 10 related to bias from data analysis. Studies with ≥7 low‐risk items were considered high quality.

### 2.6 Data synthesis and statistical methods

The analyses for diabetes and pre‐diabetes were conducted separately but using the same methods and following the same steps. Below, the analyses for diabetes were used as an example and elaborated in detail to illustrate the procedures of data synthesis. Firstly, as a basic information, the prevalence of diabetes was meta‐analysed across the included primary studies with the random‐effects model. Secondly, the proportions of each subgroup (e.g., the one with ‘2hPG elevated alone’) out of all diabetes was meta‐analysed across the included primary studies. As listed in Section 2.2, there were in total seven subgroups defined by the combination of 2hPG, FPG and HbA1c levels. Thus, seven meta‐analyses were conducted in this second step. By doing these seven meta‐analyses, the overall proportion of each subgroup out of all diabetes was obtained. Then, based on the seven proportions, area‐proportional three‐circle Venn diagrams (with the three circles representing 2hPG, FPG and HbA1c elevations, respectively) were constructed to demonstrate the overlapping pattern of patients identified by different glycaemic measures. To enhance the interpretability of results, the above meta‐analyses were conducted separately for the following four groups of primary studies: (1) studies conducted in general population and using the ADA diagnostic criteria; (2) studies conducted in general population and using non‐ADA diagnostic criteria; (3) studies conducted in patients with specific diseases and using the ADA diagnostic criteria; (4) studies conducted in patients with specific diseases and using non‐ADA diagnostic criteria. The prevalence and proportions were double‐arcsine transformed before being meta‐analysed. MetaXL^19^ was employed to conduct the meta‐analyses, because it could ensure that the pooled proportions of the seven subgroups added up to 100%. In each meta‐analysis, the pooled results (i.e., ‘overall estimate’) were presented as a point estimate with 95% confidence interval (CI). Statistical heterogeneity among studies was assessed using Cochran's *Q* test and the *I* ^2^ statistic. Subgroup analysis was conducted according to whether the participants were general population or patients with specific diseases. Subgroup analyses according to study location (Asian vs. non‐Asian countries), study quality (high vs. non‐high) and sample size (large vs. small, using the median sample size as the cutoff) were conducted to investigate the potential sources of heterogeneity. Meta‐regression was not feasible due to the small number of studies available for each meta‐analysis (≤15). Sensitivity analyses were performed to evaluate the impact of methodological quality and sample representativeness on the results. Funnel plot analysis was conducted to detect potential publication bias for meta‐analyses containing ≥10 included studies.

## 3 RESULTS

### 3.1 Results of literature search

**FIGURE 1:**
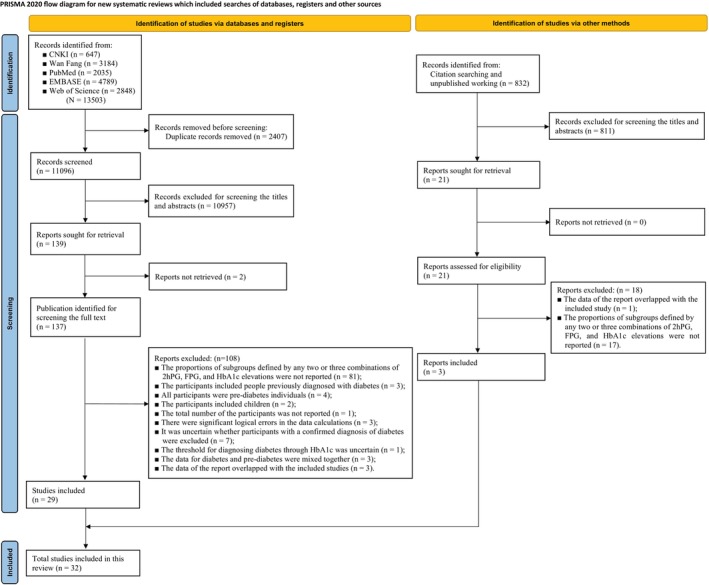
Flow diagram of search and selection. 2hPG, 2‐h post‐load glucose; CNKI, China National Knowledge Infrastructure; FPG, fasting blood glucose; HbA1c: glycosylated haemoglobin.

A total of 13 503 citations were retrieved from the five databases, and 32 studies^13^, ^14^, ^20^, ^21^, ^22^, ^23^, ^24^, ^25^, ^26^, ^27^, ^28^, ^29^, ^30^, ^31^, ^32^, ^33^, ^34^, ^35^, ^36^, ^37^, ^38^, ^39^, ^40^, ^41^, ^42^, ^43^, ^44^, ^45^, ^46^, ^47^, ^48^, ^49^ involving 400 484 participants met the eligibility criteria (Figure 1). All included studies were published in English, comprising 30 full‐text articles,^13^, ^14^, ^20^, ^21^, ^22^, ^23^, ^24^, ^25^, ^26^, ^27^, ^28^, ^29^, ^30^, ^31^, ^32^, ^33^, ^34^, ^35^, ^36^, ^37^, ^38^, ^39^, ^40^, ^41^, ^42^, ^43^, ^44^, ^45^, ^46^, ^47^ one abstract^48^ and one unpublished master's dissertation.^49^ Among the included studies, 23 are cross‐sectional studies,^13^, ^20^, ^21^, ^23^, ^25^, ^26^, ^27^, ^28^, ^29^, ^31^, ^32^, ^33^, ^34^, ^35^, ^36^, ^37^, ^40^, ^41^, ^45^, ^46^, ^47^, ^48^, ^49^ while the other nine^14^, ^22^, ^24^, ^30^, ^38^, ^39^, ^42^, ^43^, ^44^ are baseline surveys of prospective cohort studies. The studies that were seemingly eligible but excluded after full‐text review are listed in Table S2, with reasons for exclusions provided.

### 3.2 Characteristics of included studies

Twenty‐five studies (*n* = 289 094) reported on newly diagnosed diabetes,^13^, ^20^, ^21^, ^22^, ^23^, ^24^, ^25^, ^26^, ^27^, ^28^, ^29^, ^30^, ^31^, ^32^, ^33^, ^34^, ^35^, ^36^, ^37^, ^38^, ^39^, ^40^, ^41^, ^48^, ^49^ while 15 studies (*n* = 221 988) reported on newly identified pre‐diabetes^14^, ^22^, ^30^, ^31^, ^32^, ^36^, ^39^, ^40^, ^42^, ^43^, ^44^, ^45^, ^46^, ^47^, ^49^ (eight studies reporting on both^22^, ^30^, ^31^, ^32^, ^36^, ^39^, ^40^, ^49^). The characteristics of the included studies on diabetes and pre‐diabetes are summarised in Tables 1 and 2, respectively. The mean age of participants in the included studies ranged from 24 to 68 years (median of mean ages: 51). Of the diabetes studies, 15 focused on general populations^13^, ^20^, ^21^, ^22^, ^23^, ^24^, ^25^, ^26^, ^27^, ^28^, ^29^, ^30^, ^31^, ^48^, ^49^ and 10 on specific diseases,^32^, ^33^, ^34^, ^35^, ^36^, ^37^, ^38^, ^39^, ^40^, ^41^ including kidney transplant recipients (1/10, 10%), human immunodeficiency virus [HIV]‐positive individuals (1/10, 10%), coronary artery disease (3/10, 30%), mental illness (1/10, 10%), intracerebral haemorrhage (1/10, 10%), ischaemic stroke (1/10, 10%), polycystic ovary syndrome (1/10, 10%) and heart failure (HF) (1/10, 10%). For pre‐diabetes, seven studied general populations^14^, ^22^, ^30^, ^31^, ^42^, ^43^, ^49^ and eight certain‐disease groups,^32^, ^36^, ^39^, ^40^, ^44^, ^45^, ^46^, ^47^ involving HIV‐positive individuals (1/10, 10%), a history of gestational diabetes mellitus (1/10, 10%), overweight or obese (1/10, 10%), coronary artery disease (1/10, 10%), mental illness (1/10, 10%), ischaemic stroke (1/10, 10%), HF (1/10, 10%) and polycystic ovary syndrome (1/10, 10%). Both diabetes and pre‐diabetes studies were conducted across Asia (16/32, 50%), North America (6/32, 18.8%) and Europe (8/32, 25%), with diabetes research additionally including South American populations (2/32, 6.3%). The diagnostic criteria primarily followed ADA standards for both conditions (diabetes: 23/25 studies, 92%; pre‐diabetes: 12/15, 80%), with diabetes defined as: FPG ≥7.0 mmol/L, and/or 2hPG ≥11.1 mmol/L, and/or HbA1c ≥6.5%; and pre‐diabetes defined as: FPG 5.6–6.9 mmol/L, and/or 2hPG 7.8–11.0 mmol/L, and/or HbA1c 5.7%–6.4%. Alternative criteria included: (1) the Canadian Diabetes Association (CDA) standards,^32^ which defined pre‐diabetes as FPG 6.1–6.9 mmol/L and/or HbA1c 6.0%–6.4% (with 2hPG matching ADA criteria); and (2) study‐defined diagnostic thresholds, which followed ADA standards except for diabetes (HbA1c ≥6.1%)^35^ as well as pre‐diabetes diagnosis (FPG 6.1–6.9 or 5.6–6.0 mmol/L).^42^, ^43^

**TABLE 1:** Characteristics of each included study—newly diagnosed diabetes as the outcome.

Study ID	Study design	Country/region	Total sample	Participant details	Age (years)	Gender (male/female)	BMI (kg/m^2^)	Newly identified diabetes *n* (%)	Criteria of diabetes
Nazir et al.^20^	Cross‐sectional study	India	2188	Chennai adults (CURES study)	38.7 ± 12.6 (≥20)	1006/1182	22.7	333 (15.2%)	ADA
Getaneh et al.^21^	Cross‐sectional study	USA	450	Dominican adults (DIAMOND study 2003–2004)	(≥40)	–	–	72 (16.0%)	ADA
Costa et al.^22^	Cohort study	Spain	1144	FINDRISC score ≥ 15 Caucasian (DE‐PLAN study)	61.4 (Mean) (45–75)	400/744	29.9	201 (17.6%)	ADA
Herath et al.^23^	Cross‐sectional study	Sri Lanka	254	People with history of diabetes among first degree relative: a community‐based study carried out in sub‐urban locality in Southern Sri Lanka	50.5 ± 12.0 (≥20)	131/123	23.7 ± 4.9	79 (31.1%)	ADA
Law et al.^24^	Cohort study	Hong Kong	414	Urban Hong Kong population (CRISPS 3: 2005–2006)	(≥40)	–	–	81 (19.6%)	ADA
Albitres‐Flores et al.^25^	Cross‐sectional study	Peru	1500	Habitual residents in Tumbes: at least 6 months	47.6 ± 10.6 (30–69)	751/749	–	74 (4.9%)	ADA
Araneta et al.^26^	Cross‐sectional study	USA	933	Middle‐aged American recruited from community volunteers in San Diego and Hawaii	54.2 (Mean)	252/681	26.9	228 (24.4%)	ADA
Radhakrishna et al.^27^	Cross‐sectional study	India	332	South Indians adults with higher risk for diabetes^1^ were recruited at a hospital located in South India	53 (Mean) (45–61)	215/117	–	96 (28.9%)	ADA
Carrillo‐Larco et al.^28^	Cross‐sectional study	USA	3652	The US civilian non‐institutionalised population (NHANES database 1988–1994)	55.6 ± 10.4 (≥40)	1804/1848	27.9 (13.3–61.9)	463 (12.7%)	ADA
Tan and Chew^48^	Cross‐sectional study	Singapore	250	Recruited at Tan Tock Seng Hospital Diabetes Centre	–	–	–	133 (53.2%)	ADA
Gujral et al.^29^	Cross‐sectional study	India	1568	Indian living in Chennai (CARRS‐2 Study)	(≥40)	–	–	242 (15.4%)	ADA
		India	1448	Indian living in New Delhi (CARRS‐2 Study)	(≥40)	–	–	190 (13.1%)	
		USA	608	Asian Indian immigrants living in the greater San Francisco Bay and Chicago areas (MASALA Study)	(40–80)	–	–	74 (12.2%)	
Zhang et al.^13^	Cross‐sectional study	China	159 848	Chinese adults (CCDRFS 2013–2014)	(≥18)	–	–	12 781 (8.0%)	ADA
Woo et al.^30^	Cohort study	Hong Kong	1300	Urban Hong Kong population (CRISPS 4: 2010–2012)	(≥40)	–	–	101 (7.8%)	ADA
Xu et al.^31^	Cross‐sectional study	China	95 205	Civilian, non‐institutionalised Chinese adults (CDC study)	41.27 (Mean) (41.9–55.6)	48 668/46 537	22.82 (23.5–25.9)	8050 (8.5%)	ADA
Yang 2024^49^	Cross‐sectional study	USA	10 764	The US civilian non‐institutionalised population (NHANES database 2005–2016)	18–39: 35.9%; 40–59: 34.9%; ≥60: 29.2%	5327/5437	–	1016 (9.4%)	ADA
Pimentel et al.^33^	Cross‐sectional study	Brazil	122	Participants underwent kidney transplantation who were recruited at Hospital de Clínicas de Porto Alegre	46.2 ± 14.1	62/60	25.8 ± 4.2	34 (27.9%)	ADA
Coelho et al.^34^	Cross‐sectional study	Portugal	220	Participants with infected HIV and on combined antiretroviral therapy who were recruited at the Endocrinology Outpatient Clinic of São João Hospital	45.8 ± 11.5	133/87	25.32	17 (7.7%)	ADA
Tekumit et al.^35^	Cross‐sectional study	Turkey	111	Participants underwent elective isolated on‐pump coronary artery bypass grafting surgery who were recruited at Avrupa Safak Hastanesi and John F. Kennedy Hospital	(39–83)	–	21–40	74 (66.7%)	Investigators‐defined^1^
Wang et al.^36^	Cross‐sectional study	Taiwan	689	Participants underwent coronary angiography for suspected or known CAD who were recruited at Taichung Veterans General Hospital	62.23 (Mean)	568/121	25.84	193 (28.0%)	ADA
Romain et al.^32^	Cross‐sectional study	Canada and France	84	Participants with serious mental illness who were recruited at University of Montreal Hospital Research Centre and University Hospital of Montpellier	38.4 ± 12.7	41/43	35.0 ± 6.7	15 (17.9%)	ADA
Gyberg et al.^37^	Cross‐sectional study	Europe	4004	Participants with clinical evidence of CAD at a time 6–36 months before recruitment (EUROASPIRE IV)	63.6 ± 9.8 (≥18–<80)	3062/942	28.6 ± 4.3	1146 (28.6%)	ADA
Zhanget al.^38^	Cohort study	China	357	Participants with intracerebral haemorrhage (ACROSS‐China)	58.2 ± 13.3	232/125	24.52	131 (36.7%)	ADA
Zhang et al.^39^	Cohort study	China	1251	Participants with ischemic stroke (ACROSS‐China)	62.2 ± 12.6	789/452	24.85	539 (43.1%)	ADA
Zhen et al.^40^	Cross‐sectional study	China	161	Females with polycystic ovary syndrome were recruited at the Fifth Affiliated Hospital of Zhengzhou University	23.68 ± 4.23	0/161	27.4 ± 2.20	10 (6.2%)	ADA
Ishikawa et al.^41^	Cross‐sectional study	USA	237	Participants with a self‐reported physician‐diagnosis of heart failure (NHANES database 2005–2016)	65.0 ± 1.1 (≥20)	129/108	–	50 (21.1%)	ADA

*Note*: ADA: diabetes was defined as fasting blood glucose (FPG) ≥7.0 mmol/L (126 mg/dL), and/or 2‐h post‐load glucose (2hPG) value ≥11.1 mmol/L (200 mg/dL), and/or glycosylated haemoglobin (HbA1c) ≥48 mmol/mol (≥6.5%) by ADA criteria. Investigators‐defined^1^: diabetes was defined as FPG ≥7.0 mmol/L, and/or 2hPG value ≥11.1 mmol/L, and/or HbA1c ≥6.1% in this article. ‘higher risk for diabetes’ were one or more of the following: age ≥45 years, abdominal obesity (waist circumference ≥90 cm in men and ≥80 cm in women), BMI ≥23 kg/m^2^, diabetes among biological parents or siblings, hypertension, physical inactivity, dyslipidaemia (fasting serum triglycerides ≥150 mg/dL and/or high‐density lipoprotein‐cholesterol level <40 mg/dL in men and <50 mg/dL in women), known coronary artery or cerebrovascular disease and women with past gestational diabetes or delivery of a baby with birth weight ≥3.5 kg and polycystic ovarian syndrome.

Abbreviations: ACROSS‐China, the study of abnormal glucose regulation in patients with acute stroke across China; ADA, the American Diabetes Association; BMI, body mass index; CAD, coronary artery disease; CARRS, The ‘Centre for cArdiometabolism Risk Reduction in South‐Asia’ Study; CCDRFS, The China Chronic Diseases and Risk Factors Surveillance Study; CDC, the National Disease Surveillance Point System of Chinese Center for Disease Control and Prevention Study; CRISPS 4, the fourth visit of the Hong Kong Cardiovascular Risk Factor Prevalence Study; CURES, The Chennai Urban Rural Epidemiological Study; DE‐PLAN, The ‘Diabetes in Europe‐Prevention using Lifestyle, Physical Activity and Nutritional intervention’ project; DIAMOND, The Diabetes Among Dominicans and Other Minorities in Northern Manhattan study; EUROASPIRE, The European Action on Secondary Prevention through Intervention to Reduce Events; FINDRISC, the Finnish Diabetes Risk Score questionnaire; HIV, human immunodeficiency virus; MASALA, The Mediators of Atherosclerosis in South Asians Living in America study; NHANES, the National Health and Nutrition Examination Survey; USA, the United States of America.

**TABLE 2:** Characteristics of each included study—newly diagnosed pre‐diabetes as the outcome.

Study ID	Study design	Country/region	Total sample	Participant details	Age (years)	Gender (male/female)	BMI (kg/m^2^)	Newly identified diabetes *n* (%)	Criteria of pre‐diabetes
Costa et al.^22^	Cohort study	Spain	1144	FINDRISC score ≥ 15 Caucasian (DE‐PLAN study)	61.4 (Mean) (45–75)	400/744	29.9	1023 (89.4%)	ADA
Lu et al.^14^	Cohort study	China	106 493	Chinese adults (4C study)	(≥40)	–	–	76 202 (71.6%)	ADA
Saukkonen 2011^42^	Cohort study	Finland	486	Inhabitants of Oulu born in 1935	–	–	–	164 (33.7%)	Investigators‐defined^a^
Rathmann et al.^43^	Cohort study	German	896	Middle‐aged German residents (KORA S4 Study)	47.1 ± 7.9 (31–60)	461/435	26.8 ± 4.8	372 (41.1%)	Investigators‐defined^b^
			1764	Older German residents (KORA F4 Study)	67.0 ± 3.9 (61–75)	810/954	28.6 ± 4.1	535 (30.3%)	
Woo et al.^30^	Cohort study	Hong Kong	1300	Urban Hong Kong population (CRISPS 4: 2010–2012)	(≥40)	–	–	849 (65.3%)	ADA
Xu et al.^31^	Cross‐sectional study	China	95 205	Civilian, non‐institutionalised Chinese adults (CDC study)	41.27 (Mean) (41.9–55.6)	48 668/46 537	22.82 (23.5–25.9)	49 043 (51.5%)	ADA
Yang^49^	Cross‐sectional study	USA	10 764	The US civilian non‐institutionalised population (NHANES database 2005–2016)	18–39: 35.9%; 40–59: 34.9%; ≥60: 29.2%	5327/5437	–	6504 (60.4%)	ADA
Phuphuakrat et al.^45^	Cross‐sectional study	Thailand	397	HIV infected adults who received ART and had undetectable plasma viral load (HIV viral load <40 copies/mL)	47.0 ± 9.8	221/176	23.03	129 (32.5%)	ADA
Benaiges et al.^46^	Cross‐sectional study	Spain	141	Participants with a history of gestational diabetes mellitus who were recruited at the Hospital del Mar, Barcelona	34.2 ± 4.8	0/141	27.0 ± 5.1	29 (20.6%)	ADA
Cosson et al.^47^	Cross‐sectional study	France	1157	Adults overweight (BMI ≥25 kg/m^2^) or obese (BMI ≥30 kg/m^2^)	41 ± 13	195/962	37 ± 7	588 (50.8%)	ADA
Wang et al.^36^	Cross‐sectional study	Taiwan	689	Participants underwent coronary angiography for suspected or known CAD who were recruited at Taichung Veterans General Hospital	62.23 (Mean)	568/121	25.84	416 (60.4%)	ADA
Romain et al.^32^	Cross‐sectional study	Canada and France	84	Participants with serious mental illness who were recruited at University of Montreal Hospital Research Centre and University Hospital of Montpellier	38.4 ± 12.7	41/43	35.0 ± 6.7	25 (29.8%)	CDA
Stevens et al.^44^	Cohort study	Belgium	56	Participants with a history of Chronic heart failure ≥6 months and clinically stable for ≥3 months prior to the onset who were recruited from the Heart failure clinic of the Jessa hospital	68.27 (Mean)	37/19	27.68	31 (55.4%)	ADA
Zhang et al.^39^	Cohort study	China	1251	Participants with ischemic stroke (ACROSS‐China)	62.2 ± 12.6	789/452	24.85	471 (37.6%)	ADA
Zhen et al.^40^	Cross‐sectional study	China	161	Participants with polycystic ovary syndrome were recruited at the Fifth Affiliated Hospital of Zhengzhou University	23.68 ± 4.23	0/161	27.4 ± 2.20	23 (14.3%)	ADA

*Note*: ADA: Pre‐diabetes was defined as fasting blood glucose (FPG) level of 5.6–6.9 mmol/L (100–125 mg/dL), and/or 2‐h post‐load glucose (2hPG) value level of 7.8–11.0 mmol/L (140–199 mg/dL), and/or glycosylated haemoglobin (HbA1c) level of 5.7%–6.4% by ADA criteria. CDA: Pre‐diabetes was defined as FPG level of 6.1–6.9 mmol/L, and/or 2hPG value level of 7.8–11.0 mmol/L, and/or HbA1c level of 6.0%–6.4% by CDA criteria.

Abbreviations: 4C, the China Cardiometabolic Disease and Cancer Cohort; ACROSS‐China, the study of abnormal glucose regulation in patients with acute stroke across China; ADA, the American Diabetes Association; BMI, body mass index; CAD, coronary artery disease; CDA, the Canadian Diabetes Association; CDC, the National Disease Surveillance Point System of Chinese Center for Disease Control and Prevention Study; CRISPS 4, the fourth visit of the Hong Kong Cardiovascular Risk Factor Prevalence Study; DE‐PLAN, The ‘Diabetes in Europe‐Prevention using Lifestyle, Physical Activity and Nutritional intervention’ project; FINDRISC, the Finnish Diabetes Risk Score questionnaire; HIV, human immunodeficiency virus; KORA, Cooperative Health Research in the Region of Augsburg; NHANES, the National Health and Nutrition Examination Survey; USA, the United States of America.

^a^ Investigators‐defined: Pre‐diabetes was defined as FPG level of 5.6–6.0 mmol/L, and/or 2hPG value level of 7.8 to <11.1 mmol/L, and/or HbA1c level of 5.7% to <6.5%.

^b^ Investigators‐defined: Pre‐ diabetes was defined as FPG level of 6.1 to <7.0 mmol/L, and/or 2hPG value level of 7.8 to <11.1 mmol/L, and/or HbA1c level of 5.7% to <6.5%.

### 3.3 Assessment of methodological quality

The results of the methodological quality assessment based on the 10‐item tool are summarised in Table S3. The most problematic were: item 1, population representativeness (high risk in 25/32 or 78.1% studies); item 3, random selection (20/32 or 62.5%); item 2, sampling frame adequacy (14/32 or 43.8%); and item 4, non‐response bias (12/32 or 37.5%). The number of low‐risk items ranged from 5 to 10 in individual studies, with a median of 7. Notably, 24 studies (75%) had ≥7 low‐risk items, including 10 (31%) with 7 low‐risk items,^21^, ^22^, ^27^, ^33^, ^34^, ^35^, ^37^, ^40^, ^43^, ^47^ 4 studies (13%) with 8 low‐risk items,^29^, ^38^, ^39^, ^41^ 6 studies (19%) with 9 low‐risk items^20^, ^23^, ^25^, ^28^, ^31^, ^49^ and 4 studies (13%) with 10 low‐risk items.^13^, ^14^, ^24^, ^30^

### 3.4 The proportions of different combinations of 2hPG, FPG and HbA1c among participants newly detected with diabetes

**FIGURE 2:**
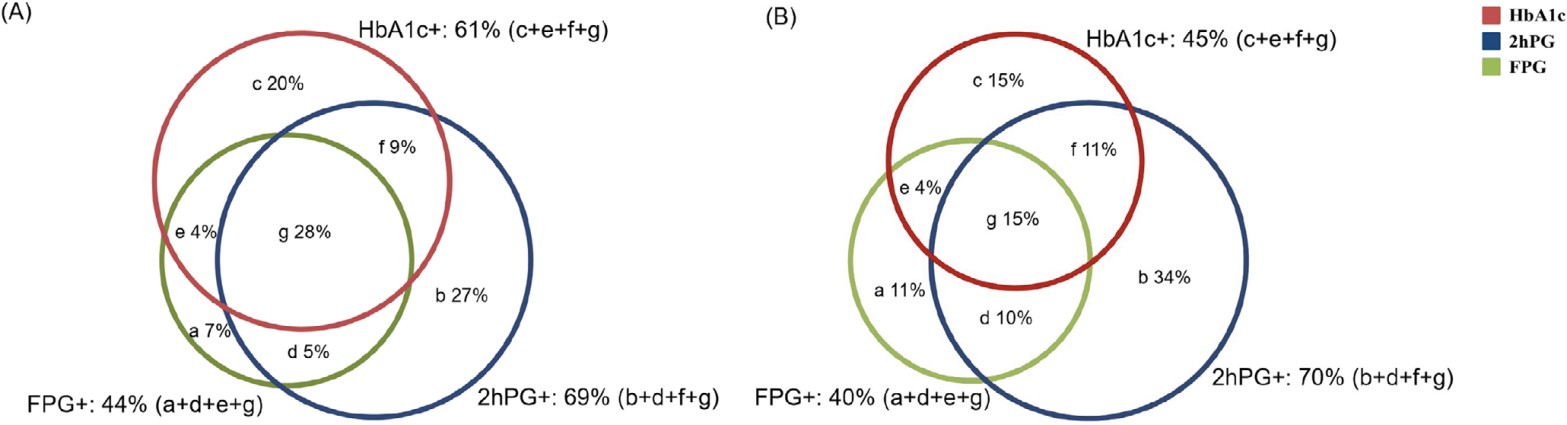
The proportions of different combinations of 2‐h post‐load glucose (2hPG), fasting plasma glucose (FPG) and glycosylated haemoglobin (HbA1c) among adult participants newly diagnosed with diabetes. (A) The general population; (B) the population with specific diseases.

Of the 25 studies reporting data on newly detected diabetes,^13^, ^20^, ^21^, ^23^, ^24^, ^25^, ^26^, ^27^, ^28^, ^29^, ^30^, ^31^, ^32^, ^33^, ^34^, ^35^, ^36^, ^37^, ^38^, ^39^, ^40^, ^41^, ^48^, ^49^ 15 were conducted in the general population and all of these adopted the ADA diagnostic criteria.^13^, ^20^, ^21^, ^22^, ^23^, ^24^, ^25^, ^26^, ^27^, ^28^, ^29^, ^30^, ^31^, ^48^, ^49^ Meta‐analysis of the 15 studies showed that the weighted prevalence of diabetes was 15% (see Table S4 for results of other scenarios). As shown in Figures 2A and S1, among adults with newly detected diabetes (*n* = 24 214), 69% (95% CI, 61%–75%; *I* ^2^ = 99%) had elevated 2hPG, 44% (95% CI, 41%–51%; *I* ^2^ = 97%) elevated FPG, and 61% (95% CI, 52%–69%; *I* ^2^ = 99%) elevated HbA1c; 11% (95% CI, 7%–16%; *I* ^2^ = 99%) had normal 2hPG but elevated FPG (regardless of their HbA1c levels); 7% (95% CI, 3%–11%; *I* ^2^ = 99%) had normal 2hPG and HbA1c but elevated FPG (i.e., isolated FPG elevation); 20% (95% CI, 12%–24%; *I* ^2^ = 99%) had normal 2hPG and normal FPG but elevated HbA1c (i.e., isolated HbA1c elevation).

Of the 10 studies^32^, ^33^, ^34^, ^35^, ^36^, ^37^, ^38^, ^39^, ^40^, ^41^ conducted in adults with specific diseases, nine^32^, ^33^, ^34^, ^36^, ^37^, ^38^, ^39^, ^40^, ^41^ used the ADA diagnostic criteria for diabetes. Meta‐analyses of these nine studies showed that the relative contributions of the three glycaemic measures to the prevalence of diabetes were largely consistent with the results observed in the general population (Figures 2B and S1). Only one study^35^ which was conducted in patients undergoing elective isolated on‐pump coronary artery bypass grafting (*n* = 111) used investigators‐defined criteria (HbA1c ≥ 6.1%). That study showed that elevated HbA1c accounted for the highest proportion of diabetes (91%), followed by elevated 2hPG (81%) and then elevated FPG (74%).

### 3.5 The proportions of different combinations of 2hPG, FPG and HbA1c among adult participants newly detected with pre‐diabetes

**FIGURE 3:**
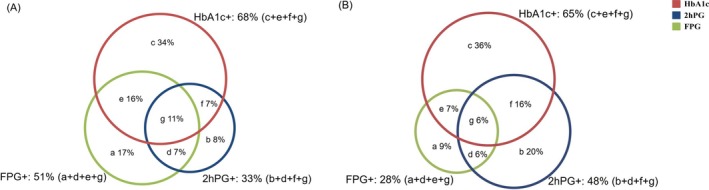
The proportions of different combinations of 2‐h post‐load glucose (2hPG), fasting plasma glucose (FPG) and glycosylated haemoglobin (HbA1c) among adult participants newly diagnosed with pre‐diabetes. (A) The general population; (B) the population with specific diseases.

Of the 15 studies reporting data on newly detected pre‐diabetes,^14^, ^22^, ^30^, ^31^, ^32^, ^36^, ^39^, ^40^, ^42^, ^43^, ^44^, ^45^, ^46^, ^47^, ^49^ 7 were conducted in the general population^14^, ^22^, ^30^, ^31^, ^42^, ^43^, ^49^ and 5 of them adopted the ADA diagnostic criteria.^14^, ^22^, ^30^, ^31^, ^49^ Meta‐analysis of these five studies showed that the weighted prevalence of pre‐diabetes was 69% (see Table S4 for the results based on other criteria or populations). As shown in Figures 3A and S2, among adults with newly detected pre‐diabetes (*n* = 133 621), 33% (95% CI, 21%–47%; *I* ^2^ = 100%) had elevated 2hPG, 51% (95% CI, 42%–60%; *I* ^2^ = 100%) elevated FPG and 68% (95% CI, 59%–75%; *I* ^2^ = 100%) elevated HbA1c; 33% (95% CI, 23%–41%; *I* ^2^ = 100%) had normal 2hPG but elevated FPG (regardless of their HbA1c levels); 17% (95% CI, 9%–23%; *I* ^2^ = 100%) had normal 2hPG and HbA1c but elevated FPG (i.e., isolated FPG elevation); 34% (95% CI, 22%–40%; *I* ^2^ = 100%) had normal 2hPG and normal FPG but elevated HbA1c (i.e., isolated HbA1c elevation). Two general population‐based studies adopted investigators‐defined diagnostic criteria for pre‐diabetes.^42^, ^43^ A Finland study (*n* = 486; FPG cutoff: 5.6–6.0 mmol/L) demonstrated that 2hPG elevation contributed most to pre‐diabetes (58%), followed by HbA1c (42%) and then FPG (27%) elevations,^42^ while a German study of middle‐aged and older adults (*n* = 2660; FPG cutoff: 6.1–6.9 mmol/L) found that HbA1c elevation contributed most to the prevalence of pre‐diabetes (71%), followed by 2hPG (37%) and then FPG (23%) elevations.^43^

Of the eight studies conducted in individuals with specific diseases,^32^, ^36^, ^39^, ^40^, ^44^, ^45^, ^46^, ^47^ seven used the ADA diagnostic criteria for pre‐diabetes.^36^, ^39^, ^40^, ^44^, ^45^, ^46^, ^47^ Analysis of these seven studies also showed that HbA1c elevation was the largest contributor to the prevalence of pre‐diabetes (65%), followed by 2hPG (48%) and least by FPG (28%) (Figures 3B and S2). The remaining study, which was conducted in patients with serious mental illness, applied the pre‐diabetes criteria of the CDA, but its sample size (*n* = 84) was too small to provide a robust estimate of the overlapping pattern of pre‐diabetes defined by different glycaemic measures.^32^

### 3.6 Secondary analyses

The results of subgroup analyses (Tables [Link], [Link]) showed that the prevalence of diabetes and pre‐diabetes and the contribution of HbA1c elevation to them were both higher in the small studies, and the contribution of HbA1c elevation was also higher in Asian studies. The difference between subgroups defined by study quality was not statistically significant. The contributions of 2hPG and FPG elevations to the prevalence of diabetes and pre‐diabetes did not vary considerably across subgroups either. Sensitivity analyses according to sample representativeness in both the general population and individuals with specific diseases yielded consistent results with the main analyses (Figures [Link], [Link]). The funnel plots (Figures [Link], [Link]) were all asymmetric. However, due to the significant heterogeneity among studies in all the meta‐analyses, it was possible that the asymmetry was caused by heterogeneity, rather than publication bias; thus, interpretation should be cautious.^50^

## 4 DISCUSSION

This systematic review showed that 2hPG elevation is the largest contributor (69%) to the prevalence of diabetes, followed by HbA1c (61%) elevation and then FPG elevation (44%). Inclusion of FPG as a diagnostic measure increases the number of cases by 11% relatively on top of those already identified by 2hPG, and inclusion of HbA1c further increases it by 20% on top of those already identified by 2hPG and FPG. Isolated FPG and HbA1c elevations account for 7% and 20% of all diabetes, respectively. For pre‐diabetes, HbA1c elevation is the largest contributor (68%) to the prevalence, followed by FPG elevation and then 2hPG elevation. Isolated FPG and HbA1c elevations account for 17% and 34% of all pre‐diabetes, respectively. According to the estimate of the International Diabetes Federation, approximately 589 million people are currently living with diabetes.^1^ Based on this number and the findings of this systematic review, the numbers of cases with diabetes or pre‐diabetes newly identified by FPG and HbA1c were estimated and shown in Table 3. However, it should be noted that these were extrapolations based on data from multiple sources, with inherent assumptions and potential for overestimation, and should therefore be interpreted as rough approximations with appropriate caution.

**TABLE 3:** Number of new cases with diabetes or pre‐diabetes caused by including fasting blood glucose (FPG) and glycosylated haemoglobin (HbA1c) as diagnostic measures.

	Diabetes	Pre‐diabetes
Total number of cases	588.7 million^a^	1923.6 million^b^
2hPG elevated		
% of the total prevalence	69	33
Number of cases	406.2 million	634.8 million
FPG or HbA1c elevated		
% of the total prevalence	73	92
Number of cases	429.8 million	1769.7 million
FPG elevated alone		
% of the total prevalence	44	51
Number of cases	259.0 million	981.1 million
HbA1c elevated alone		
% of the total prevalence	61	68
Number of cases	359.1 million	1308.1 million

Abbreviation: 2hPG, 2‐h post‐load glucose.

^a^ This number was estimated by International Diabetes Federation (IDF).^1^

^b^ This number was estimated by authors of this study based on (1) the number of impaired glucose tolerance estimated by IDF and (2) the percentage of impaired glucose tolerance out of all pre‐diabetes, that is, 51%, as estimated by the present study (see Figure 3A).

While FPG and HbA1c elevations contribute significantly to the case number and prevalence of diabetes, their association with diabetic complications has been less certain. Previous studies consistently showed that 2hPG was still associated with increased risks of cardiovascular disease and mortality after adjusting for FPG, HbA1c and other non‐glycaemic risk factors,^13^, ^14^, ^15^ suggesting that it was an ‘independent’ risk factor. By contrast, although numerous studies have shown that FPG and HbA1c were associated with the risk of diabetic complications when used individually, this association often disappeared after controlling for 2hPG, especially in the case of FPG.^13^, ^14^, ^15^ These findings cast doubt on whether FPG and HbA1c are independent risk factors of diabetic complications and whether treatment of individuals with isolated FPG or HbA1c elevation, particularly the former (which accounts 7% of all diabetes), would confer clinically important benefits in terms of preventing diabetic complications such as cardiovascular disease. The reason why 2hPG is more predictive of cardiovascular disease may be related to its stronger indication of postprandial glucose metabolism and insulin resistance. Postprandial glucose spikes contribute significantly to oxidative stress and endothelial dysfunction,^51^ key drivers of atherosclerosis. 2hPG also correlates closely with insulin resistance,^52^ which is a major risk factor for cardiovascular disease. By contrast, FPG mainly captures basal glycaemia, which is more dependent on hepatic glucose output^53^ and may not fully represent systemic metabolic dysfunction, while HbA1c reflects an average glucose but misses acute fluctuations linked to vascular damage. Having said that, we acknowledge that the evidence on the role of FPG and HbA1c in independently increasing risks of diabetic complications (both macrovascular and microvascular) is insufficient, and more research on this topic is warranted.

The management of pre‐diabetes is more controversial than that of diabetes, both because the huge number of people labelled as having this condition (two‐thirds of which being FPG and HbA1c elevations, as shown by this study) and because of the uncertainty around the causal relation between FPG and HbA1c and diabetic complications. Like the case of diabetes, a large body of evidence shows that the three glycaemic measures, when used individually, were each associated with increased cardiovascular disease and mortality, but few studies examined their ‘independent effects’ on the outcomes after controlling for the confounding caused by the other two glycaemic measures. One of the few studies that did so was Lind et al., which found that the 2hPG level in people with IGT was associated with an increased risk of cardiovascular disease after adjusting for FPG, 1‐h post‐load glucose, HbA1c and other major risk factors,^54^ whereas FPG and HbA1c were not associated with the outcome when analysed in the same model. However, a limitation of that study was its relatively small sample size (*n* = 504) and number of outcome events (*n* = 34). Other studies conducted in people with pre‐diabetes were mostly focused on one or two glycaemic measures only and thus were not able to clarify whether the observed association with outcomes (if any) resulted from confounding caused by other glycaemic measures.^55^ Because of this, whether it is justifiable to intervene with or ‘manage’ pre‐diabetes defined by FPG and HbA1c is open to question. Indeed, several randomised controlled trials have shown that lifestyle interventions and even some pharmacological treatment in people with pre‐diabetes did not lead to reductions in cardiovascular disease or mortality, although the progression to diabetes was slowed down to some extent.^56^, ^57^ Thus, further prospective studies evaluating the independent effects of FPG and HbA1c levels on future diabetic complications among the pre‐diabetes population are warranted.

The findings of this study have implications for the diagnosis and management of hyperglycaemia, especially pre‐diabetes. The ultimate goal of diagnosis is to identify the individuals at higher risk of complications and mitigate the risk through effective interventions. This study revealed that including FPG and HbA1c as diagnostic measures doubled the size of the pre‐diabetes population (with isolated FPG or HbA1c elevations accounting for 17% + 34% of all pre‐diabetes, see above) as compared with using 2hPG alone, whereas, as mentioned above, it remains largely unclear whether the newly identified individuals were at higher risk of diabetic complications, with limited evidence suggesting that they were not. Furthermore, as recognised by ADA,^58^ the efficacy of interventions for primary prevention of type 2 diabetes has been demonstrated mainly among individuals with pre‐diabetes defined by 2hPG (i.e., IGT), not for those with isolated FPG or HbA1c elevations,^59^ and pharmacological interventions for primary prevention are not superior to lifestyle modifications.^10^ For HbA1c elevation, which dominates the pre‐diabetes, there are more issues that need to be taken into account. First, the accuracy of HbA1c measurement could be affected by multiple factors, such as anaemia, haemoglobin variants, chronic liver disease, splenomegaly, rheumatoid arthritis and drugs or diseases that cause haemolysis.^60^ Second, HbA1c levels vary with age and ethnicity,^61^ which means that applying the same cutoff (e.g., the widely used ‘≥6.5%’) to different populations could have very different resource implications. This is particularly relevant to low‐ and middle‐income countries. Taken as a whole, currently available evidence suggests that the use of FPG and HbA1c for screening for or diagnosing pre‐diabetes and pharmacological interventions for treating pre‐diabetes (as recommended by some guidelines^9^, ^10^) are worth reconsideration or at least refinement, especially in view of the huge population affected and the unintended harms that may be caused to them, for example, anxiety and over‐treatment.

This study has some limitations. First, due to the small number of studies included in individual meta‐analyses (particularly those for pre‐diabetes) or significant heterogeneity among studies, funnel plots were either not feasible or could be misleading, which limited our ability to investigate the possibility of publication bias. Second, previous studies have shown that the phenotypes of pre‐diabetes differed with gender, age and other characteristics.^62^ However, due to the lack of detailed information on these characteristics in the included studies, we were unable to present the distribution of different combinations of 2hPG, FPG and HbA1c levels in different subgroups defined by age, gender and body mass index. Third, as with other meta‐analyses of single‐group proportions, we observed significant heterogeneity among studies. This heterogeneity was unlikely caused by different diagnostic criteria because the studies using non‐ADA criteria were few (≤2 in all analytic scenarios) and small in sample size and were analysed separately. Subgroup analyses showed that the prevalence of diabetes and pre‐diabetes and the contribution of HbA1c elevation to them were both higher in the small studies, and the contribution of HbA1c elevation was also higher in Asian studies. Given that large studies typically produce more robust estimates than do small ones, the actual prevalence of diabetes could be lower than 15% (the overall estimate from meta‐analysis of all studies) and close to 11% (the overall estimate from meta‐analysis of large studies). The much higher contribution of HbA1c to the prevalence of diabetes and pre‐diabetes in Asian populations (70.4% vs. 42.5% in non‐Asian for diabetes) suggests that the use of HbA1c for diagnosis in these populations should be particularly carefully considered, taking into account the large number of new cases and the magnitude of possible treatment efficacy in these cases.

## 5 CONCLUSION

This study shows that the largest contributors to the prevalence of diabetes and pre‐diabetes are 2hPG and HbA1c, respectively. FPG and HbA1c measurements identify a large number of new cases, with isolated FPG and HbA1c elevations accounting for over a quarter of all diabetes and more than half of all pre‐diabetes. However, not all of the new cases, particularly those with isolated FPG or HbA1c elevation, are at higher risk of diabetic complications or could benefit from treatment. While FPG and HbA1c are convenient to measure, current evidence shows that 2hPG could better stratify the risk of macrovascular complications, and those with 2hPG elevation have been consistently demonstrated to be able to benefit from treatment. Thus, refinement of the diagnostic algorithms for diabetes and pre‐diabetes may be warranted, for instance, through modifying the strategy of joint use of different glycaemic measures or reducing the number of diagnostic measures. To better inform clinical decision‐making and policy development, more longitudinal studies focusing on the prognostic validity of each glycaemic measure and the treatment benefit in individuals identified by them, particularly FPG and HbA1c, are needed, and both macrovascular and microvascular complications of diabetes should be examined as important outcomes in such studies.

## AUTHOR CONTRIBUTIONS

Conceptualization: **Zuyao Yang**. Acquisition and analysis of data: **Xue Xue**, **Jiaxuan Li** and **Bingrui Zhang**. Writing—original draft preparation: **Xue Xue**. Writing—review and editing: **Xue Xue**, **Zuyao Yang**, **Jiaxuan Li**, **Wenxiao Zheng**, **Bingrui Zhang**, **Jiayue Zhang** and **Shuting Wang**. All authors have read and agreed to the published version of the manuscript.

## FUNDING INFORMATION

This work was supported by the Teaching Development and Language Enhancement Grant 2022–2025 (project code: 4171066) and the Research Data Management Development Fund (Round 2) of The Chinese University of Hong Kong.

## CONFLICT OF INTEREST STATEMENT

The authors declare no conflicts of interest.

## PEER REVIEW

The peer review history for this article is available at https://www.webofscience.com/api/gateway/wos/peer-review/10.1111/dom.70130.

## Supporting information


**Table S1.** Searching strategies for electronic databases.

**Table S2.** The list of excluded studies during full‐text searching and screening process.

**Table S3.** Methodological quality evaluation of the included studies.

**Table S4.** The prevalence of diabetes and pre‐diabetes.

**Table S5.** Characteristics of subgroup analyses—newly diagnosed diabetes as the outcome.

**Table S6.** Characteristics of subgroup analyses—newly diagnosed pre‐diabetes as the outcome.

**Table S7.** Characteristics of subgroup analyses—newly diagnosed diabetes by 2hPG criteria.

**Table S8.** Characteristics of subgroup analyses—newly diagnosed diabetes by FPG criteria.

**Table S9.** Characteristics of subgroup analyses—newly diagnosed diabetes by HbA1c criteria.

**Table S10.** Characteristics of subgroup analyses—newly diagnosed pre‐diabetes by 2hPG criteria.

**Table S11.** Characteristics of subgroup analyses—newly diagnosed pre‐diabetes by FPG criteria.

**Table S12.** Characteristics of subgroup analyses—newly diagnosed pre‐diabetes by HbA1c criteria.

**Figure S1.** Forest plot of the proportions of each combination of 2‐h post‐load glucose, fasting plasma glucose and glycosylated haemoglobin among adult participants newly diagnosed with diabetes. (A) The general population; (B)the population with specific diseases.

**Figure S2.** Forest plot of the proportions of each combination of 2‐h post‐load glucose, fasting plasma glucose and glycosylated haemoglobin among adult participants newly diagnosed with pre‐diabetes. (A) The general population; (B) the population with specific diseases.

**Figure S3.** Sensitivity analyses (retaining only studies with nationally or regionally representative samples)—The proportions of different combinations of 2‐h post‐load glucose, fasting plasma glucose and glycoslyated haemoglobin among adult participants newly diagnosed with diabetes. (A) The general population; (B) the population with specific diseases.

**Figure S4.** Sensitivity analyses (retaining only studies with nationally or regionally representative samples)—the proportions of different combinations of 2‐h post‐load glucose, fasting plasma glucose and glycosylated haemoglobin among general population newly diagnosed with pre‐diabetes.

**Figure S5.** Sensitivity analyses (retaining only studies with nationally or regionally representative samples)—forest plot of the proportions of each combination of 2‐h post‐load glucose, fasting plasma glucose and glycosylated haemoglobin among adult participants newly diagnosed with diabetes. (A) The general population; (B) the population with specific diseases.

**Figure S6.** Sensitivity analyses (retaining only studies with nationally or regionally representative samples)—forest plot of the proportions of each combination of 2‐h post‐load glucose, fasting plasma glucose and glycosylated haemoglobin among general population newly diagnosed with pre‐diabetes.

**Figure S7.** Sensitivity analyses (retaining only studies with ≥7 low‐risk items)—the proportions of different combinations of 2‐h post‐load glucose, fasting plasma glucose and glycosylated haemoglobin among adult participants newly diagnosed with diabetes. (A) The general population; (B) the population with specific diseases.

**Figure S8.** Sensitivity analyses (retaining only studies with ≥7 low‐risk items)—the proportions of different combinations of 2‐h post‐load glucose, fasting plasma glucose and glycosylated haemoglobin among general adult population newly diagnosed with pre‐diabetes.

**Figure S9.** Sensitivity analyses (retaining only studies with ≥7 low‐risk items)—Forest plot of the proportions of each combination of 2‐h post‐load glucose, fasting plasma glucose and glycosylated haemoglobin among adult participants newly diagnosed with diabetes. (A) The general population; (B) the population with specific diseases.

**Figure S10.** Sensitivity analyses (retaining only studies with≥7 low‐risk items)—forest plot of the proportions of each combination of 2‐h post‐load glucose, fasting plasma glucose and glycosylated haemoglobin among the adult population with specific diseases newly diagnosed with pre‐diabetes.

**Figure S11.** Funnel plot for the meta‐analysis of contribution of glycosylated haemoglobin elevation to prevalence of diabetes in general population.

**Figure S12.** Funnel plot for the meta‐analysis of contribution of 2‐h post‐load glucose elevation to prevalence of diabetes in general population.

**Figure S13.** Funnel plot for the meta‐analysis of contribution of fasting plasma glucose diagnosis elevation to prevalence of diabetes in general population.

**Figure S14.** Funnel plot for the meta‐analysis of contribution of 2‐h post‐load glucose diagnosis elevation to prevalence of diabetes in specific diseases population.

**Figure S15.** Funnel plot for the meta‐analysis of contribution of fasting plasma glucose diagnosis elevation to prevalence of diabetes in specific diseases population.

## Data Availability

All data in the study are included in the article/Supporting Information; further inquiries can be directed to the corresponding author.
