# Supplementary material for: Contributions of 2‐h post‐load glucose, fasting blood glucose and glycosylated haemoglobin elevations to the prevalence of diabetes and pre‐diabetes in adults: A systematic analysis of global data
Source: Diabetes Obes Metab. 2025 Sep 15;27(12):7285–98. doi: 10.1111/dom.70130 (PMC12587253; doi:10.1111/dom.70130)
Supplement: Supplementary file 1 — Table S1. Searching strategies for electronic databases. [file DOM-27-7285-s007.docx]

**Supplementary Table 1 Searching strategies for electronic databases**

| **Databases** | **Searching strategies** | **Results (n)** |
| --- | --- | --- |
| PubMed | #1. ("fasting*"[MeSH:noexp] OR "Fasting/blood*"[MeSH:noexp] OR "fast*"[All Fields]) AND "glucose"[All Fields]  #2. "FPG"[All Fields] OR "FBG"[All Fields]  #3. #1 OR #2  #4. "post load"[All Fields] OR "postload"[All Fields] OR "post prandial"[All Fields] OR "postchallenge plasma glucose"[All Fields] OR "oral glucose tolerance test"[All Fields] OR "glucose tolerance test"[MeSH:noexp] OR "OGTT"[All Fields] OR "2hPG"[All Fields] OR "2h PG"[All Fields] OR "2 hPG"[All Fields] OR "2h-PG"[All Fields] OR "2-h plasma glucose"[All Fields] OR "2-hour plasma glucose"[All Fields] OR "2-h post-challenge glucose"[All Fields] OR "2-hour post-load glucose"[All Fields] OR "2-h PG"[All Fields] OR "2-h glucose"[All Fields] OR "2-hour glucose"[All Fields] OR "2h glucose"[All Fields] OR "PCPG"[All Fields] OR "blood glucose 2 h"[All Fields] OR "2-hr glucose"[All Fields]  #5. "Glycated Hemoglobin"[MeSH:noexp] OR "GHb"[All Fields] OR "Hemoglobin"[All Fields] OR "HbA1c"[All Fields] OR "A1C"[All Fields]  #6. "cohort studies"[MeSH:noexp] OR "longitudinal studies"[MeSH:noexp] OR "follow up studies"[MeSH:noexp] OR "prospective studies"[MeSH:noexp] OR "retrospective studies"[MeSH:noexp] OR "cohort"[All Fields] OR "longitudinal"[All Fields] OR "followed"[All Fields] OR "follow-up"[All Fields] OR "prospective*"[All Fields]  #7."Cross-Sectional Studies"[MeSH:noexp] OR "Prevalence"[MeSH:noexp] OR ("association"[All Fields] and "stud*"[All Fields]) OR "cross-sectional[All Fields] OR "cross sectional[All Fields] OR "prevalence"[All Fields] OR "transversal"[All Fields] OR "survey"[All Fields] OR "association"[Title] OR "associations"[Title]  #8.#6 OR #7  #9.#3 AND #4 AND #5 AND #8 | 2035 |
| Web of Science | #1.TS=((fast* AND glucose) OR (FPG OR FBG))  #2.TS = (post load OR postload OR post prandial OR postchallenge plasma glucose OR oral glucose tolerance test OR glucose tolerance test OR OGTT OR 2hPG OR 2h PG OR 2 hPG OR 2h-PG OR 2-h plasma glucose OR 2-hour plasma glucose OR 2-h post-challenge glucose OR 2-hour post-load glucose OR 2-h PG OR 2-h glucose OR 2-hour glucose OR 2h glucose OR PCPG OR blood glucose 2 h OR 2-hr glucose)  #3.TS = (Glycated Hemoglobin OR GHb OR Hemoglobin OR HbA1c OR A1C)  #4.((TS=(association AND stud*)) OR TS=(cohort OR prospective* OR follow-up OR follow up OR followed OR longitudinal OR retrospective OR cross-sectional OR cross sectional OR transversal OR prevalence OR survey)) OR TI=(association OR associations)  #5.#1 AND #2 AND #3 AND #4 | 2848 |
| EMBASE | #1.('fasting'/exp OR 'fast*':ti,ab,kw) AND ('glucose'/exp OR 'glucose':ti,ab,kw)  #2.'fasting blood glucose'/exp OR 'fasting blood glucose level'/exp OR 'FPG':ti,ab,kw OR 'FBG':ti,ab,kw  #3.#1 OR #2  #4.'post load':ti,ab,kw OR 'postload':ti,ab,kw OR 'post prandial':ti,ab,kw OR 'postchallenge plasma glucose':ti,ab,kw OR 'oral glucose tolerance test'/exp OR 'oral glucose tolerance test':ti,ab,kw OR 'glucose tolerance test':ti,ab,kw OR 'OGTT':ti,ab,kw OR '2hPG':ti,ab,kw OR '2h PG':ti,ab,kw OR '2 hPG':ti,ab,kw OR '2h-PG':ti,ab,kw OR '2-h plasma glucose':ti,ab,kw OR '2-hour plasma glucose':ti,ab,kw OR '2-h post-challenge glucose':ti,ab,kw OR '2-hour post-load glucose':ti,ab,kw OR '2-h PG':ti,ab,kw OR '2-h glucose':ti,ab,kw OR '2-hour glucose':ti,ab,kw OR '2h glucose':ti,ab,kw OR 'PCGC':ti,ab,kw OR 'blood glucose 2 h':ti,ab,kw OR '2-hr glucose':ti,ab,kw  #5.'Glycated Hemoglobin'/exp OR 'Glycated Hemoglobin':ti,ab,kw OR 'GHb':ti,ab,kw OR 'Hemoglobin a1c'/exp OR 'Hemoglobin':ti,ab,kw OR 'HbA1c':ti,ab,kw OR 'A1C':ti,ab,kw  #6.'cohort analysis'/exp OR 'longitudinal study'/exp OR 'follow up'/exp OR 'prospective study'/exp OR 'cohort':ti,ab,kw OR 'longitudinal':ti,ab,kw OR 'followed':ti,ab,kw OR 'follow-up':ti,ab,kw OR 'prospective*':ti,ab,kw  #7.'cross-sectional study'/exp OR 'prevalence'/exp OR 'cross-sectional':ti,ab,kw OR 'cross sectional':ti,ab,kw OR 'prevalence':ti,ab,kw OR 'transversal':ti,ab,kw OR ('association':ti,ab,kw AND 'stud*':ti,ab,kw) OR 'association':ti OR 'associations':ti OR 'survey':ti,ab,kw  #8.#6 OR #7  #9.#3 AND #4 AND #5 AND #8 | 4789 |
| Chinese databases:  CNKI  Wan Fang | (TKA='空腹血糖') AND (TKA='餐后*血糖') AND (TKA='糖化血红蛋白') AND (TKA='队列*' OR TKA='前膽*' OR TKA='随访*' OR TKA='纵向*' OR TKA='横断面*' OR TKA='现况*' OR TKA='调查' OR TKA='现患*')  主题:(空腹 AND 血糖) AND 主题:(餐后 AND 血糖) AND 主题:(糖化血红蛋白) AND 主题:(队列* OR 前瞻* OR 随访* OR 纵向* OR 横断面* OR 现况* OR 调查 OR 现患*) | 647    3184 |
