# Supplementary material for: Contributions of 2‐h post‐load glucose, fasting blood glucose and glycosylated haemoglobin elevations to the prevalence of diabetes and pre‐diabetes in adults: A systematic analysis of global data
Source: Diabetes Obes Metab. 2025 Sep 15;27(12):7285–98. doi: 10.1111/dom.70130 (PMC12587253; doi:10.1111/dom.70130)
Supplement: Supplementary file 2 — Table S2. The list of excluded studies during full‐text searching and screening process. [file DOM-27-7285-s024.docx]

**SupplementaryTable 2 The list of excluded studies during full-text searching and screening process**

| No. | Study ID | Title | The reason for exclusion |
| --- | --- | --- | --- |
| 1 | Woo 2014 | Cardio-metabolic characteristics of Hong Kong Chinese adults with prediabetes diagnosed by HbA1c but not by glycaemic criteria. | The proportions of subgroups defined by any two or three combinations of elevated 2hPG, FPG, and HbA1c levels were not reported. |
| 2 | Midthjell 2010 | Comparison of HbA1c and OGTT in the diagnosis of diabetes in a highrisk population. The HUNT-DE-PLAN Study, Norway. | The proportions of subgroups defined by any two or three combinations of elevated 2hPG, FPG, and HbA1c levels were not reported. |
| 3 | Kim 2014 | Comparison of HBA1C and OGTT to diagnose diabetes in korean children. | The proportions of subgroups defined by any two or three combinations of elevated 2hPG, FPG, and HbA1c levels were not reported. |
| 4 | Manley 2010 | Comparison of HbA1c cut-point of ≥6.5% for diagnosis of diabetes with WHO criteria and an algorithm combining HbA1c and FPG. | The proportions of subgroups defined by any two or three combinations of elevated 2hPG, FPG, and HbA1c levels were not reported. |
| 5 | Byrne 2013 | Detecting undiagnosed diabetes mellitus in patients with acute ischaemic stroke. | The proportions of subgroups defined by any two or three combinations of elevated 2hPG, FPG, and HbA1c levels were not reported. |
| 6 | Niflioglu 2011 | Detection of diabetes mellitus and pre-diabetes with A1C, fasting plasma glucose and oral glucose tolerance test. A1C based screening may be more diagnostic test for diabetes mellitus. | The proportions of subgroups defined by any two or three combinations of elevated 2hPG, FPG, and HbA1c levels were not reported. |
| 7 | Sahay 2021 | Diabetes Prevalence and Risk Factors in Patients With Chronic Mental Illness on Second-Generation Antipsychotics. | The proportions of subgroups defined by any two or three combinations of elevated 2hPG, FPG, and HbA1c levels were not reported. |
| 8 | Oliveira 2015 | Diagnosis of diabetes mellitus in a population of obese patients: OGTT or A1c/fasting plasma glucose. | The proportions of subgroups defined by any two or three combinations of elevated 2hPG, FPG, and HbA1c levels were not reported. |
| 9 | Roeder 2014 | Diagnosis of gestational diabetes in the first trimester is associated with a greater need for pharmacotherapy. | The proportions of subgroups defined by any two or three combinations of elevated 2hPG, FPG, and HbA1c levels were not reported. |
| 10 | Garvin 2019 | Diagnostic Concordance of Hemoglobin A1c for Insulin Sensitivity Testing in Polycystic Ovarian Syndrome. | The proportions of subgroups defined by any two or three combinations of elevated 2hPG, FPG, and HbA1c levels were not reported. |
| 11 | Foo 2010 | Glycated hemoglobin is a more sensitive test compared to fasting plasma glucose in diagnosing diabetes mellitus in the obese. | The proportions of subgroups defined by any two or three combinations of elevated 2hPG, FPG, and HbA1c levels were not reported. |
| 12 | Khanh 2014 | HbA1c, fasting blood glucose, and OGTT in the diagnosis of diabetes mellitus in Ho Chi Minh City, Vietnam. | The proportions of subgroups defined by any two or three combinations of elevated 2hPG, FPG, and HbA1c levels were not reported. |
| 13 | Villasmil 2023 | Hemoglobin A1c and Fasting Blood Glucose Correlate Poorly with Oral Glucose Tolerance in Turner Syndrome. | The proportions of subgroups defined by any two or three combinations of elevated 2hPG, FPG, and HbA1c levels were not reported. |
| 14 | Kapadia 2012 | Hemoglobin A1c measurement for the diagnosis of type 2 diabetes in Korean children. | The proportions of subgroups defined by any two or three combinations of elevated 2hPG, FPG, and HbA1c levels were not reported. |
| 15 | Schnell 2012 | Impaired glucose tolerance and cardiovascular risk. | The proportions of subgroups defined by any two or three combinations of elevated 2hPG, FPG, and HbA1c levels were not reported. |
| 16 | Cabre 2010 | Moving to the new HbA1c diagnostic criteria has a deep impact on prevalence of gluco-metabolic abnormalities among high-risk Spanish population. | The proportions of subgroups defined by any two or three combinations of elevated 2hPG, FPG, and HbA1c levels were not reported. |
| 17 | Xiliang Z 2014 | New American diabetes association diagnostic criteria for screening previously unknown diabetes in patients undergoing elective coronary angiography. | The proportions of subgroups defined by any two or three combinations of elevated 2hPG, FPG, and HbA1c levels were not reported. |
| 18 | Baptista 2014 | Oral glucose tolerance test is still needed in patients with coronary artery disease. | The proportions of subgroups defined by any two or three combinations of elevated 2hPG, FPG, and HbA1c levels were not reported. |
| 19 | Ogbonna 2010 | Performance of HbA1c as a diagnostic test for diabetes and pre-diabetes. | The proportions of subgroups defined by any two or three combinations of elevated 2hPG, FPG, and HbA1c levels were not reported. |
| 20 | Schernthaner 2011 | The diagnostic dilemma for diabetes in patients with morbid obesity. | The proportions of subgroups defined by any two or three combinations of elevated 2hPG, FPG, and HbA1c levels were not reported. |
| 21 | Feder 2015 | The diagnostic dilemma for type 2 diabetes in patients with morbid obesity. | The proportions of subgroups defined by any two or three combinations of elevated 2hPG, FPG, and HbA1c levels were not reported. |
| 22 | Byrne 2012 | Which test should be used to screen for type 2 diabetes mellitus in acute ischaemic stroke? | The proportions of subgroups defined by any two or three combinations of elevated 2hPG, FPG, and HbA1c levels were not reported. |
| 23 | Viveca 2014 | Oral glucose tolerance test superior to HbA1c when screening for undiagnosed diabetes in patients with coronary artery disease. A report from EuroAspire IV. | The proportions of subgroups defined by any two or three combinations of elevated 2hPG, FPG, and HbA1c levels were not reported. |
| 24 | XJ Chen 2019 | Clinical significance of different diagnostic indexes of diabetes. (in Chinese) | The proportions of subgroups defined by any two or three combinations of elevated 2hPG, FPG, and HbA1c levels were not reported. |
| 25 | Hsia 2020 | Implications of the Hemoglobin Glycation Index on the Diagnosis of Prediabetes and Diabetes. | The proportions of subgroups defined by any two or three combinations of elevated 2hPG, FPG, and HbA1c levels were not reported. |
| 26 | CH Lei 2011 | Value of Fasting Blood Glucose Combined with QuantitativePostprandial Urine Glucose for Diabetes Screeningin Community high risk population.(in Chinese) | The proportions of subgroups defined by any two or three combinations of elevated 2hPG, FPG, and HbA1c levels were not reported. |
| 27 | Adamu 2011 | Comparative performance of HbA1c 6.5% for FPG ≥7.0 vs 2hr PG≥11.1 criteria for diagnosis of type 2 diabetes. | The proportions of subgroups defined by any two or three combinations of elevated 2hPG, FPG, and HbA1c levels were not reported. |
| 28 | Gianchandani 2011 | Evaluation of hemoglobin A1c criteria to assess preoperative diabetes risk in cardiac surgery patients. | The proportions of subgroups defined by any two or three combinations of elevated 2hPG, FPG, and HbA1c levels were not reported. |
| 29 | Zemlin 2011 | HbA1c of 6.5% to diagnose diabetes mellitus -- does it work for us? -- the Bellville South Africa study. | The proportions of subgroups defined by any two or three combinations of elevated 2hPG, FPG, and HbA1c levels were not reported. |
| 30 | Alqahtani 2013 | Use of Glycated Hemoglobin in the Diagnosis of Diabetes Mellitus and Pre-diabetes and Role of Fasting Plasma Glucose, Oral Glucose Tolerance Test. | The proportions of subgroups defined by any two or three combinations of elevated 2hPG, FPG, and HbA1c levels were not reported. |
| 31 | Roest 2022 | Oral Glucose Tolerance Test for the Screening of Glucose Intolerance Long Term Post-Heart Transplantation. | The proportions of subgroups defined by any two or three combinations of elevated 2hPG, FPG, and HbA1c levels were not reported. |
| 32 | Prakaschandra 2018 | FASTING PLASMA GLUCOSE AND THE HBA1C ARE NOT OPTIMAL SCREENING MODALITIES FOR THE DIAGNOSIS OF NEW DIABETES IN PREVIOUSLY UNDIAGNOSED ASIAN INDIAN COMMUNITY PARTICIPANTS | The proportions of subgroups defined by any two or three combinations of elevated 2hPG, FPG, and HbA1c levels were not reported. |
| 33 | Okosun 2012 | Applicability of a combination of hemoglobin A1c and fasting plasma glucose in population-based prediabetes screening. | The proportions of subgroups defined by any two or three combinations of elevated 2hPG, FPG, and HbA1c levels were not reported. |
| 34 | M Mo 2013 | Combining glycosylated hemoglobin A1c and fasting plasma glucose for diagnosis of type 2 diabetes in Chinese adults | The proportions of subgroups defined by any two or three combinations of elevated 2hPG, FPG, and HbA1c levels were not reported. |
| 35 | Maynard 2007 | Noninvasive type 2 diabetes screening - Superior or sensitivity to fasting plasma glucose and A1C | The proportions of subgroups defined by any two or three combinations of elevated 2hPG, FPG, and HbA1c levels were not reported. |
| 36 | Manley 2016 | Can HbA1c detect undiagnosed diabetes in acute medical hospital admissions? | The proportions of subgroups defined by any two or three combinations of elevated 2hPG, FPG, and HbA1c levels were not reported. |
| 37 | G Li 2018 | Evaluation of ADA HbA1c criteria in the diagnosis of pre-diabetes and diabetes in a population of Chinese adolescents and young adults at high risk for diabetes: a cross-sectional study | The proportions of subgroups defined by any two or three combinations of elevated 2hPG, FPG, and HbA1c levels were not reported. |
| 38 | Kim 2019 | Comparison of HbA1c and OGTT for the diagnosis of type 2 diabetes in children at risk of diabetes | The proportions of subgroups defined by any two or three combinations of elevated 2hPG, FPG, and HbA1c levels were not reported. |
| 39 | Kibirige 2023 | Diagnostic accuracy of two confirmatory tests for diabetes mellitus in adult Ugandans with recently diagnosed tuberculosis. | The proportions of subgroups defined by any two or three combinations of elevated 2hPG, FPG, and HbA1c levels were not reported. |
| 40 | Józwa 2021 | HbA1c Screening for Diabetes in Patients with Acute Coronary Syndrome: A Worthwhile Test or a Pitfall? | The proportions of subgroups defined by any two or three combinations of elevated 2hPG, FPG, and HbA1c levels were not reported. |
| 41 | Gignac 2023 | Glycated Hemoglobin Is Suboptimal for the Screening of Prediabetes and Type 2 Diabetes in Adults With Nonalcoholic Fatty Liver Disease. | The proportions of subgroups defined by any two or three combinations of elevated 2hPG, FPG, and HbA1c levels were not reported. |
| 42 | Ellison 2005 | HbA1C screening for undiagnosed diabetes in New Zealand | The proportions of subgroups defined by any two or three combinations of elevated 2hPG, FPG, and HbA1c levels were not reported. |
| 43 | Doerr 2011 | Oral glucose tolerance test and HbA1c for diagnosis of diabetes in patients undergoing coronary angiography the Silent Diabetes Study. | The proportions of subgroups defined by any two or three combinations of elevated 2hPG, FPG, and HbA1c levels were not reported. |
| 44 | Cetin 2020 | The Compatibility of Hemoglobin A1c with Oral Glucose Tolerance Test and Fasting Plasma Glucose. | The proportions of subgroups defined by any two or three combinations of elevated 2hPG, FPG, and HbA1c levels were not reported. |
| 45 | Costa 2013 | Shifting from glucose diagnosis to the new HbA1c diagnosis reduces the capability of the Finnish Diabetes Risk Score (FINDRISC) to screen for glucose abnormalities within a real-life primary healthcare. preventive strategy | The proportions of subgroups defined by any two or three combinations of elevated 2hPG, FPG, and HbA1c levels were not reported. |
| 46 | Agbozo 2018 | Accuracy of glycosuria, random blood glucose and risk factors as selective screening tools for gestational diabetes mellitus in comparison with universal diagnosing. | The proportions of subgroups defined by any two or three combinations of elevated 2hPG, FPG, and HbA1c levels were not reported. |
| 47 | Hird 2016 | Burden of diabetes and first evidence for the utility of HbA1c for diagnosis and detection of diabetes in urban black South Africans: The durban diabetes study. | The proportions of subgroups defined by any two or three combinations of elevated 2hPG, FPG, and HbA1c levels were not reported. |
| 48 | Q Li 2011 | Combined utility of hemoglobin A1c and glycated albumin in diabetic screening. | The proportions of subgroups defined by any two or three combinations of elevated 2hPG, FPG, and HbA1c levels were not reported. |
| 49 | López 2017 | Diabetic by HbA1c, normal by OGTT: A frequent finding in the Mexico City Diabetes Study. | The proportions of subgroups defined by any two or three combinations of elevated 2hPG, FPG, and HbA1c levels were not reported. |
| 50 | Coetzee 2023 | Early postpartum HbA1c after hyperglycemia first detected in pregnancy—Imperfect but not without value. | The proportions of subgroups defined by any two or three combinations of elevated 2hPG, FPG, and HbA1c levels were not reported. |
| 51 | Saunajoki 2022 | Elevated One-Hour Post-Load Glucose Is Independently Associated with Albuminuria: A Cross-Sectional Population Study. | The proportions of subgroups defined by any two or three combinations of elevated 2hPG, FPG, and HbA1c levels were not reported. |
| 52 | Kumpatla 2013 | Evaluation of performance of A1c and FPG tests for screening newly diagnosed diabetes defined by an OGTT among tuberculosis patients-A study from India. | The proportions of subgroups defined by any two or three combinations of elevated 2hPG, FPG, and HbA1c levels were not reported. |
| 53 | S Lin 2014 | Glycated haemoglobin A1c for diagnosing diabetes in Chinese subjects over 50 years old: A community-based cross-sectional study. | The proportions of subgroups defined by any two or three combinations of elevated 2hPG, FPG, and HbA1c levels were not reported. |
| 54 | Kasujja 2022 | Glycated haemoglobin and fasting plasma glucose tests in the screening of outpatients for diabetes and abnormal glucose regulation in Uganda: A diagnostic accuracy study. | The proportions of subgroups defined by any two or three combinations of elevated 2hPG, FPG, and HbA1c levels were not reported. |
| 55 | Hutchinson 2012 | Glycated hemoglobin in diagnosis of diabetes mellitus and pre-diabetes; validation by oral glucose tolerance test. The Tromsø OGTT Study. | The proportions of subgroups defined by any two or three combinations of elevated 2hPG, FPG, and HbA1c levels were not reported. |
| 56 | Moreira 2019 | Glycated hemoglobin in the diagnosis of diabetes mellitus in a Semi-Urban Brazilian population. | The proportions of subgroups defined by any two or three combinations of elevated 2hPG, FPG, and HbA1c levels were not reported. |
| 57 | Apostolakis 2018 | HbA1c presents low sensitivity as a post-pregnancy screening test for both diabetes and prediabetes in Greek women with history of gestational diabetes mellitus. | The proportions of subgroups defined by any two or three combinations of elevated 2hPG, FPG, and HbA1c levels were not reported. |
| 58 | Picón 2012 | Hemoglobin A1c versus oral glucose tolerance test in postpartum diabetes screening. | The proportions of subgroups defined by any two or three combinations of elevated 2hPG, FPG, and HbA1c levels were not reported. |
| 59 | Mokta 2017 | High incidence of abnormal glucose metabolism in acute coronary syndrome patients at a moderate altitude: A sub-Himalayan study. | The proportions of subgroups defined by any two or three combinations of elevated 2hPG, FPG, and HbA1c levels were not reported. |
| 60 | Bur 2003 | Is fasting blood glucose a reliable parameter for screening for diabetes in hypertension? | The proportions of subgroups defined by any two or three combinations of elevated 2hPG, FPG, and HbA1c levels were not reported. |
| 61 | Hanna 2011 | Limitations of glycosylated haemoglobin (HbA1c) in diabetes screening. | The proportions of subgroups defined by any two or three combinations of elevated 2hPG, FPG, and HbA1c levels were not reported. |
| 62 | Tucker 2020 | Limited agreement between classifications of diabetes and prediabetes resulting from the ogtt, hemoglobin a1c, and fasting glucose tests in 7412 U.S. adults. | The proportions of subgroups defined by any two or three combinations of elevated 2hPG, FPG, and HbA1c levels were not reported. |
| 63 | Misnikova 2014 | Optimizing screening procedures for early detection of glycemic disorders. | The proportions of subgroups defined by any two or three combinations of elevated 2hPG, FPG, and HbA1c levels were not reported. |
| 64 | Staimez 2022 | Potential misclassification of diabetes and prediabetes in the U.S.: Mismatched HbA1c and glucose in NHANES 2005–2016. | The proportions of subgroups defined by any two or three combinations of elevated 2hPG, FPG, and HbA1c levels were not reported. |
| 65 | Wilmot 2013 | Prevalence of diabetes and impaired glucose metabolism in younger 'at risk' UK adults: Insights from the STAND programme of research. | The proportions of subgroups defined by any two or three combinations of elevated 2hPG, FPG, and HbA1c levels were not reported. |
| 66 | Mohammad 2021 | Prevalence of prediabetes and undiagnosed diabetes among kuwaiti adults: A cross-sectional study. | The proportions of subgroups defined by any two or three combinations of elevated 2hPG, FPG, and HbA1c levels were not reported. |
| 67 | Bandela 2022 | PREVALENCE OF UNDIAGNOSED PREDIABETES / DIABETES IN PERIODONTITIS IN YOUNG (18-50 YEARS) POPULATION. | The proportions of subgroups defined by any two or three combinations of elevated 2hPG, FPG, and HbA1c levels were not reported. |
| 68 | Claesson 2015 | Role of HbA1c in post-partum screening of women with gestational diabetes mellitus. | The proportions of subgroups defined by any two or three combinations of elevated 2hPG, FPG, and HbA1c levels were not reported. |
| 69 | Shestakova 2016 | Russian multicentre type 2 diabetes screening program in patients with cardiovascular disease. | The proportions of subgroups defined by any two or three combinations of elevated 2hPG, FPG, and HbA1c levels were not reported. |
| 70 | Yates 2013 | Screening for new-onset diabetes after kidney transplantation: Limitations of fasting glucose and advantages of afternoon glucose and glycated hemoglobin. | The proportions of subgroups defined by any two or three combinations of elevated 2hPG, FPG, and HbA1c levels were not reported. |
| 71 | Y Wang 2017 | Serum 1,5-anhydroglucitol level as a screening tool for diabetes mellitus in a community-based population at high risk of diabetes. | The proportions of subgroups defined by any two or three combinations of elevated 2hPG, FPG, and HbA1c levels were not reported. |
| 72 | Al Amiri 2015 | The prevalence, risk factors, and screening measure for prediabetes and diabetes among Emirati overweight/obese children and adolescents. | The proportions of subgroups defined by any two or three combinations of elevated 2hPG, FPG, and HbA1c levels were not reported. |
| 73 | Ussif 2019 | Validation of diagnostic utility of fasting plasma glucose and HbA1c in stable renal transplant recipients one year after transplantation. | The proportions of subgroups defined by any two or three combinations of elevated 2hPG, FPG, and HbA1c levels were not reported. |
| 74 | Kyung-Soo 2016 | Diagnostic value of hemoglobin A1C in post-partum screening of women with gestational diabetes mellitus. | The proportions of subgroups defined by any two or three combinations of elevated 2hPG, FPG, and HbA1c levels were not reported. |
| 75 | Lopez Rios 2010 | Differences in cardiovascular risk profile of diabetic subjects discordantly classified by diagnostic criteria based on glycated haemoglobin and oral glucose tolerance test. | The proportions of subgroups defined by any two or three combinations of elevated 2hPG, FPG, and HbA1c levels were not reported. |
| 76 | Viswanathan 2012 | Evaluation of performance of A1C and fpg tests for screening prediabetes and newly diagnosed diabetes defined by an OGTT among tuberculosis patients-a study from India. | The proportions of subgroups defined by any two or three combinations of elevated 2hPG, FPG, and HbA1c levels were not reported. |
| 77 | Araneta 2014 | Optimum BMI cut points to screen Asian Americans for type 2 diabetes: The UCSD filipino health study and the North Kohala study. | The proportions of subgroups defined by any two or three combinations of elevated 2hPG, FPG, and HbA1c levels were not reported. |
| 78 | Karnchanasorn 2016 | Comparison of the Current Diagnostic Criterion of HbA1c with Fasting and 2-Hour Plasma Glucose Concentration. | The proportions of subgroups defined by any two or three combinations of elevated 2hPG, FPG, and HbA1c levels were not reported. |
| 79 | Najeeb 2015 | A comparative study of fasting, postprandial blood glucose and glycated hemoglobin for diagnosing diabetes mellitus in staff members of MMIMSR, Mullana, Ambala MMIMSR、Mullana、Ambala. | The proportions of subgroups defined by any two or three combinations of elevated 2hPG, FPG, and HbA1c levels were not reported. |
| 80 | Marini 2012 | Comparison of A1C, fasting and 2-h post-load plasma glucose criteria to diagnose diabetes in Italian Caucasians. | The proportions of subgroups defined by any two or three combinations of elevated 2hPG, FPG, and HbA1c levels were not reported. |
| 81 | Hempe 2023 | Effect of biological variation in HbA1c and blood glucose on the diagnosis of prediabetes. | The proportions of subgroups defined by any two or three combinations of elevated 2hPG, FPG, and HbA1c levels were not reported. |
| 82 | Larissa 2016 | Identifying probable diabetes mellitus among hispanics/latinos from four U.S. cities: findings from the hispanic community health study/study of latinos. | The participants included people previously diagnosed with diabetes. |
| 83 | XX He 2017 | An additional measurement of glycated albumin can help prevent missed diagnosis of diabetes in Chinese population. | The participants included people previously diagnosed with diabetes. |
| 84 | Kim 2011 | The Utility of HbA1c as a Diagnostic Criterion of Diabetes. | The participants included people previously diagnosed with diabetes. |
| 85 | Chadha 2020 | Reproducibility of a prediabetes classification in a contemporary population. | All participants were pre-diabetes individuals. |
| 86 | Chilelli 2014 | Screening with HbA1c identifies only one in two individuals with diagnosis of prediabetes at oral glucose tolerance test: findings in a real-world Caucasian population. | All participants were pre-diabetes individuals. |
| 87 | Lee 2021 | Impaired fasting glucose levels in overweight or obese subjects for screening of type 2 diabetes in Korea | All participants were pre-diabetes individuals. |
| 88 | Kim 2016 | Is an Oral Glucose Tolerance Test Still Valid for Diagnosing Diabetes Mellitus? | All participants were pre-diabetes individuals. |
| 89 | Minkova 2022 | Prediabetes in obesity adolescents: Frequency, structure, relationship with body mass index. | The participants included children. |
| 90 | Bonito 2022 | Phenotypes of prediabetes and metabolic risk in Caucasian youths with overweight or obesity. | The participants included children. |
| 91 | Okosun 2015 | Improving Detection of Prediabetes in Children and Adults: Using Combinations of Blood Glucose Tests. | The total number of the participants was not reported. We contacted the corresponding author by email and did not receive a response. |
| 92 | Brix 2023 | Assessment of Prediabetes in Patients with Morbid Obesity (MO). | There were significant logical errors in the data calculations. We contacted the corresponding author by email and did not receive a response. |
| 93 | FJ Guo 2014 | Use of HbA1c for Diagnoses of Diabetes and Prediabetes: Comparison with Diagnoses Based on Fasting and 2-Hr Glucose Values and Effects of Gender, Race, and Age. | There were significant logical errors in the data calculations. We contacted the corresponding author by email and did not receive a response. |
| 94 | James 2011 | Implications of alternative definitions of prediabetes for prevalence in U.S. adults. | There were significant logical errors in the data calculations. We contacted the corresponding author by email and did not receive a response. |
| 95 | Lopez-Lopez 2018 | The simultaneous assessment of glycosylated hemoglobin, fasting plasma glucose and oral glucose tolerance test does not improve the detection of type 2 diabetes mellitus in Colombian adults. | It is uncertain whether participants with a confirmed diagnosis of diabetes were excluded. |
| 96 | Simon 1985 | Comparison of glycosylated hemoglobin and fasting plasma glucose with two-hour post-load plasma glucose in the detection of diabetes mellitus. | It was uncertain whether participants with a confirmed diagnosis of diabetes were excluded. |
| 97 | Y Lin 2012 | Glycated hemoglobin, diabetes mellitus, and cardiovascular risk in a cross-sectional study among She Chinese population. | It was uncertain whether participants with a confirmed diagnosis of diabetes were excluded. |
| 98 | McGing 2014 | An audit of HbA1c vs oral glucose tolerance test in the diagnosis of diabetes and pre-diabetes. | It was uncertain whether participants with a confirmed diagnosis of diabetes were excluded. |
| 99 | Magnussen 2011 | Hemoglobin A1c as a tool for the diagnosis of type 2 diabetes in 208 premenopausal women with polycystic ovary syndrome | It was uncertain whether participants with a confirmed diagnosis of diabetes were excluded. |
| 100 | Hage 2013 | Fasting glucose, HbA1c, or oral glucose tolerance testing for the detection of glucose abnormalities in patients with acute coronary syndromes. | It was uncertain whether participants with a confirmed diagnosis of diabetes were excluded. |
| 101 | Megia 2012 | The usefulness of HbA1c in postpartum reclassification of gestational diabetes. | It was uncertain whether participants with a confirmed diagnosis of diabetes were excluded. |
| 102 | Tuomilehto 2013 | How to detect previously undiagnosed diabetes in patients with acute stroke. | The threshold for diagnosing diabetes through HbA1c was uncertain. The email address of the corresponding author was not available, we could not contact him. |
| 103 | Sumne 2015 | Detection of abnormal glucose tolerance in Africans is improved by combining A1c with fasting glucose: The Africans in America study. | The data for diabetes and pre-diabetes were mixed together. |
| 104 | Gooding 2014 | Diagnosing dysglycemia in adolescents with polycystic ovary syndrome. | The data for diabetes and pre-diabetes were mixed together. |
| 105 | S WU 2014 | Glycated hemoglobin independently or in combination with fasting plasma glucose versus oral glucose tolerance test to detect abnormal glycometabolism in acute ischemic stroke: a Chinese cross-sectional study | The data for diabetes and pre-diabetes were mixed together. |
| 106 | YH Zhang 2012 | Diabetes and pre-diabetes as determined by glycated haemoglobin A1c and glucose levels in a developing southern Chinese population | Duplicate data with reference 27. |
| 107 | Cowie 2010 | Prevalence of Diabetes and High Risk for Diabetes Using A1C Criteria in the U.S.Population in 1988 –2006. | Duplicate data with reference 24. |
| 108 | Menke 2018 | Contributions of A1c, fasting plasma glucose, and 2-hour plasma glucose to prediabetes prevalence: NHANES 2011-2014. | Duplicate data with reference 45. |
| 109 | Y Xu 2013 | Prevalence and Control of Diabetes in Chinese Adults. | Duplicate data with reference 27. |
| 110 | Nazaimoon 2013 | Prevalence of diabetes in Malaysia and usefulness of HbA1c as a diagnostic criterion. | The proportions of subgroups defined by any two or three combinations of elevated 2hPG, FPG, and HbA1c levels were not reported. |
| 111 | Mohan 2010 | A1C Cut Points to Define Various Glucose Intolerance Groups in Asian Indians. | The proportions of subgroups defined by any two or three combinations of elevated 2hPG, FPG, and HbA1c levels were not reported. |
| 112 | SL Wu 2013 | HbA1c and the diagnosis of diabetes and prediabetes in a middle-aged and elderly Han population from northwest China | The proportions of subgroups defined by any two or three combinations of elevated 2hPG, FPG, and HbA1c levels were not reported. |
| 113 | Lorenzo 2010 | A1C between 5.7 and 6.4% as a marker for identifying pre-diabetes, insulin sensitivity and secretion, and cardiovascular risk factors: the Insulin Resistance Atherosclerosis Study (IRAS). | The proportions of subgroups defined by any two or three combinations of elevated 2hPG, FPG, and HbA1c levels were not reported. |
| 114 | Shabir 2013 | Validity of glycated haemoglobin to diagnose new onset diabetes after transplantation. | The proportions of subgroups defined by any two or three combinations of elevated 2hPG, FPG, and HbA1c levels were not reported. |
| 115 | Eide2015 | Limitations of hemoglobin A1c for the diagnosis of posttransplant diabetes mellitus. | The proportions of subgroups defined by any two or three combinations of elevated 2hPG, FPG, and HbA1c levels were not reported. |
| 116 | Marini2012 | Cardiometabolic risk profiles and carotid atherosclerosis in individuals with prediabetes identified by fasting glucose, postchallenge glucose, and hemoglobin A1c criteria. | The proportions of subgroups defined by any two or three combinations of elevated 2hPG, FPG, and HbA1c levels were not reported. |
| 117 | Farhan2012 | Comparison of HbA1c and oral glucose tolerance test for diagnosis of diabetes in patients with coronary artery disease. | The proportions of subgroups defined by any two or three combinations of elevated 2hPG, FPG, and HbA1c levels were not reported. |
| 118 | Mulder2012 | Comparison of diagnostic criteria to detect undiagnosed diabetes in hyperglycaemic patients with acute coronary syndrome. | The proportions of subgroups defined by any two or three combinations of elevated 2hPG, FPG, and HbA1c levels were not reported. |
| 119 | Y Bao2010 | Glycated haemoglobin A1c for diagnosing diabetes in Chinese population: cross sectional epidemiological survey. | The proportions of subgroups defined by any two or three combinations of elevated 2hPG, FPG, and HbA1c levels were not reported. |
| 120 | Meijnikman2017 | Not performing an OGTT results in significant underdiagnosis of (pre)diabetes in a high risk adult Caucasian population. | The proportions of subgroups defined by any two or three combinations of elevated 2hPG, FPG, and HbA1c levels were not reported. |
| 121 | Fonville2013 | Prevalence of prediabetes and newly diagnosed diabetes in patients with a transient ischemic attack or stroke. | The proportions of subgroups defined by any two or three combinations of elevated 2hPG, FPG, and HbA1c levels were not reported. |
| 122 | Hutchinson2012 | Glycated haemoglobin A1c in diagnosis of diabetes mellitus and pre-diabetes; validation by oral glucose tolerance test. The Tromsø OGTT Study. | The proportions of subgroups defined by any two or three combinations of elevated 2hPG, FPG, and HbA1c levels were not reported. |
| 123 | Kim2011 | Comparison of hemoglobin A1c with fasting plasma glucose and 2-h postchallenge glucose for risk stratification among women with recent gestational diabetes mellitus. | The proportions of subgroups defined by any two or three combinations of elevated 2hPG, FPG, and HbA1c levels were not reported. |
| 124 | Cavagnolli2011 | HbA1c measurement for the diagnosis of diabetes: is it enough? | The proportions of subgroups defined by any two or three combinations of elevated 2hPG, FPG, and HbA1c levels were not reported. |
| 125 | McCane1994 | Comparison of tests for glycated haemoglobin and fasting and two hour plasma glucose concentrations as diagnostic methods for diabetes. | The proportions of subgroups defined by any two or three combinations of elevated 2hPG, FPG, and HbA1c levels were not reported. |
| 126 | Engelgau1997 | Comparison of fasting and 2-hour glucose and HbAlc levels for diagnosing diabetes: diagnostic criteria and performance revisited. | The proportions of subgroups defined by any two or three combinations of elevated 2hPG, FPG, and HbA1c levels were not reported. |
| 127 | Olos2011 | Prevalence of abnormal glucose regulation and its optimal screening in patients with acute STEMI. | Full text was not available. |
| 128 | Naim2019 | Prevalence of diabetes mellitus and impaired glucose tolerance in cirrhosis patients. | Full text was not available. |
