# Supplementary material for: Contributions of 2‐h post‐load glucose, fasting blood glucose and glycosylated haemoglobin elevations to the prevalence of diabetes and pre‐diabetes in adults: A systematic analysis of global data
Source: Diabetes Obes Metab. 2025 Sep 15;27(12):7285–98. doi: 10.1111/dom.70130 (PMC12587253; doi:10.1111/dom.70130)
Supplement: Supplementary file 3 — Table S3. Methodological quality evaluation of the included studies. [file DOM-27-7285-s008.doc]

Supplementary Table 3 Methodological quality evaluation of the included studies

| Study ID | External Validity | | | | | Internal Validity | | | | | The number of ‘Y’ |
| --- | --- | --- | --- | --- | --- | --- | --- | --- | --- | --- | --- |
|  | Item 1 | Item 2 | Item 3 | Item 4 | Item 5 | Item 6 | Item 7 | Item 8 | Item 9 | Item 10 |  |
| Adamsha 2012^20^ | N | Y | Y | Y | Y | Y | Y | Y | Y | Y | 9 |
| Ana Rita 2018^34^ | N | N | N | Y | Y | Y | Y | Y | Y | Y | 7 |
| Ana 2015^33^ | N | N | N | Y | Y | Y | Y | Y | Y | Y | 7 |
| Angsana 2020^45^ | N | N | N | N | Y | Y | Y | Y | Y | Y | 6 |
| Asqual 2011^21^ | N | N | N | Y | Y | Y | Y | Y | Y | Y | 7 |
| Benaiges 2013^46^ | N | N | N | N | Y | Y | Y | Y | Y | N | 5 |
| Cosson 2011^47^ | N | N | N | Y | Y | Y | Y | Y | Y | Y | 7 |
| Costa 2011^22^ | N | Y | N | N | Y | Y | Y | Y | Y | Y | 7 |
| Gujral 2019^29^ | N | Y | N | Y | Y | Y | Y | Y | Y | Y | 8 |
| Hayrettin 2010^35^ | N | N | N | Y | Y | Y | Y | Y | Y | Y | 7 |
| Herath 2017^23^ | N | Y | Y | Y | Y | Y | Y | Y | Y | Y | 9 |
| JL Lu 2019^14^ | Y | Y | Y | Y | Y | Y | Y | Y | Y | Y | 10 |
| JS Wang 2015^36^ | N | N | N | N | Y | Y | Y | Y | Y | Y | 6 |
| Lawrence 2010^24^ | Y | Y | Y | Y | Y | Y | Y | Y | Y | Y | 10 |
| Leonardo 2020^25^ | N | Y | Y | Y | Y | Y | Y | Y | Y | Y | 9 |
| Maria 2010^26^ | N | N | N | N | Y | Y | Y | Y | Y | N | 5 |
| Pedapati 2018^27^ | N | N | N | Y | Y | Y | Y | Y | Y | Y | 7 |
| Rodrigo 2023^28^ | Y | Y | Y | N | Y | Y | Y | Y | Y | Y | 9 |
| Romain 2016^32^ | N | N | N | N | Y | Y | Y | Y | Y | Y | 6 |
| Stevens 2014^44^ | N | N | N | N | Y | Y | Y | Y | Y | Y | 6 |
| Tan 2012^48^ | N | N | N | N | Y | Y | N | Y | Y | Y | 5 |
| Tuula 2011^42^ | N | N | N | Y | Y | Y | Y | Y | Y | N | 6 |
| Viveca 2015^37^ | N | Y | Y | N | Y | Y | Y | Y | Y | N | 7 |
| Wolfgang 2012^43^ | N | Y | N | N | Y | Y | Y | Y | Y | Y | 7 |
| X Zhang 2021^13^ | Y | Y | Y | Y | Y | Y | Y | Y | Y | Y | 10 |
| XM Zhang 2018(1)^38^ | N | Y | N | Y | Y | Y | Y | Y | Y | Y | 8 |
| XM Zhang 2018(2)^39^ | N | Y | N | Y | Y | Y | Y | Y | Y | Y | 8 |
| Y XU 2016^31^ | Y | Y | Y | Y | Y | Y | Y | Y | Y | N | 9 |
| YC Woo 2015^30^ | Y | Y | Y | Y | Y | Y | Y | Y | Y | Y | 10 |
| YQ Zhen 2014^40^ | N | N | N | Y | Y | Y | Y | Y | Y | Y | 7 |
| Yuta 2023^41^ | N | Y | Y | Y | Y | Y | Y | Y | Y | N | 8 |
| ZY Yang 2024^49^ | Y | Y | Y | N | Y | Y | Y | Y | Y | Y | 9 |

**Abbreviations:** N: No (HIGH RISK); Y: (LOW RISK).

**Notes:**

Item 1. Was the study’s target population a close representation of the national population in relation to relevant variables?

Item 2. Was the sampling frame a true or close representation of the target population?

Item 3. Was some form of random selection used to select the sample, OR was a census undertaken?

Item 4. Was the likelihood of nonresponse bias minimal?

Item 5. Were data collected directly from the subjects (as opposed to a proxy)?

Item 6. Was an acceptable case definition used in the study?

Item 7. Was the study instrument that measured the parameter of interest shown to have validity and reliability?

Item 8. Was the same mode of data collection used for all subjects?

Item 9. Was the length of the shortest prevalence period for the parameter of interest appropriate?

Item 10. Were the numerator(s) and denominator(s) for the parameter of interest appropriate?
