# Supplementary material for: Contributions of 2‐h post‐load glucose, fasting blood glucose and glycosylated haemoglobin elevations to the prevalence of diabetes and pre‐diabetes in adults: A systematic analysis of global data
Source: Diabetes Obes Metab. 2025 Sep 15;27(12):7285–98. doi: 10.1111/dom.70130 (PMC12587253; doi:10.1111/dom.70130)
Supplement: Supplementary file 4 — Table S4. The prevalence of diabetes and pre‐diabetes. [file DOM-27-7285-s022.docx]

**Supplementary Table 4. The prevalence of diabetes and pre-****diabetes**

| Participants | The prevalence of diabetes | | The prevalence of pre-diabetes | |
| --- | --- | --- | --- | --- |
|  | Diagnosis of Diabetes According to ADA Standards | Diagnosis of Diabetes According to ADA and Investigators-defined Standards | Diagnosis of pre-Diabetes According to ADA Standards | Diagnosis of pre-Diabetes According to ADA, CDA and Investigators-defined Standards |
| General adults | 15.34%(13.79%-16.95%) | 15.34%(13.79%-16.95%) | 68.82%(56.89%-79.64%) | 55.59%(43.76%-67.12%) |
| Adults with specific diseases | 23.18%(16.83%-30.19%) | 26.81%(19.82%-34.43%) | 37.90%(27.46%-48.93%) | 36.95%(27.25%-47.20%) |
| All adults | 18.34%(16.13%-20.66%) | 19.52%(17.23%-21.91%) | 51.30%(43.13%-16.32%) | 45.81%(37.71%-54.02%) |

**Abbreviations:** ADA: the American Diabetes Association; CDA: the Canadian Diabetes Association.
