# Supplementary material for: Contributions of 2‐h post‐load glucose, fasting blood glucose and glycosylated haemoglobin elevations to the prevalence of diabetes and pre‐diabetes in adults: A systematic analysis of global data
Source: Diabetes Obes Metab. 2025 Sep 15;27(12):7285–98. doi: 10.1111/dom.70130 (PMC12587253; doi:10.1111/dom.70130)
Supplement: Supplementary file 5 — Table S5. Characteristics of subgroup analyses—newly diagnosed diabetes as the outcome. [file DOM-27-7285-s016.docx]

**Supplementary Table 5 Characteristics of subgroup analyses**—**newly diagnosed diabetes as the outcome**

| **Subgroups** | **No. of studies** | **Sample** | **Prevalence**  **（95% CI）** | **Heterogeneity**  **of subgroup**  **(I^2^)** | **Test for subgroup differences**  **(*P* value)** |
| --- | --- | --- | --- | --- | --- |
| **Study location** |  |  |  |  |  |
| General adults | 15 | 281858 | 15.33% (13.79%-16.95%) |  | 0.34 |
| Asian | 9 | 263415 | 16.26% (14.47%-18.13%) | 99% |  |
| Non-Asian | 6 | 18443 | 13.49% (09.23%-18.07%) | 98% |  |
| Adults with specific diseases | 9 | 7236 | 23.18% (16.83%-30.19%) |  | 0.48 |
| Asian | 4 | 2458 | 26.94% (14.63%-40.50%) | 98% |  |
| Non-Asian | 5 | 4667 | 20.04% (11.70%-29.47%) | 95% |  |
| **Study Quality*** |  |  |  |  |  |
| General adults | 15 | 281858 | 15.33% (13.79%-16.95%) |  | 0.06 |
| High quality | 13 | 280675 | 13.09 (11.86%-14.38%) | 98% |  |
| Non-high quality | 2 | 1183 | 38.18% (10.22%-68.58%) | 99% |  |
| Adults with specific diseases | 9 | 7236 | 23.18% (16.83%-30.19%) |  | 0.92 |
| High quality | 7 | 6352 | 23.11% (15.13%-31.95%) | 98% |  |
| Non high quality | 2 | 773 | 23.73% (14.58%-33.86%) | 76% |  |
| **Sample (Divided by median)**^#^ |  |  |  |  |  |
| General adults | 15 | 281858 | 15.33% (13.79%-16.95%) |  | <0.01 |
| Large sample | 10 | 280158 | 11.81% (10.60%-13.08%) | 98% |  |
| Small sample | 5 | 1700 | 29.01% (17.36%-41.41%) | 97% |  |
| Adults with specific diseases | 9 | 7236 | 23.18% (16.83%-30.19%) |  | 0.06 |
| Large sample | 2 | 5255 | 35.17% (22.03%-49.08%) | 99% |  |
| Small sample | 7 | 1870 | 19.73% (11.58%-28.97%) | 95% |  |

Note: *Studies with ≥7 low-risk items were considered high-quality.

^#^The total sample of the study, ≥800 was considered large sample;＜800 was considered small sample.
