# Supplementary material for: Contributions of 2‐h post‐load glucose, fasting blood glucose and glycosylated haemoglobin elevations to the prevalence of diabetes and pre‐diabetes in adults: A systematic analysis of global data
Source: Diabetes Obes Metab. 2025 Sep 15;27(12):7285–98. doi: 10.1111/dom.70130 (PMC12587253; doi:10.1111/dom.70130)
Supplement: Supplementary file 6 — Table S6. Characteristics of subgroup analyses—newly diagnosed pre‐diabetes as the outcome. [file DOM-27-7285-s010.docx]

**Supplementary Table 6 Characteristics of subgroup analyses**—**newly diagnosed pre-diabetes as the outcome**

| **Subgroups** | **No. of studies** | **Sample** | **Prevalence**  **（95% CI）** | **Heterogeneity**  **of subgroup**  **(I^2^)** | **Test for subgroup differences**  **(*P* value)** |
| --- | --- | --- | --- | --- | --- |
| **Study location** |  |  |  |  |  |
| General adults | 5 | 214906 | 68.82% (56.89%-79.64%) |  | 0.42 |
| Asian | 3 | 202998 | 63.16% (46.52%-78.67%) | 100% |  |
| Non-Asian | 2 | 11908 | 76.88% (43.04%-99.99%) | 100% |  |
| Adults with specific diseases | 7 | 3852 | 37.90% (27.46%-48.93%) |  | 0.69 |
| Asian | 4 | 2498 | 35.45% (20.02%-52.04%) | 98% |  |
| Non-Asian | 3 | 1354 | 41.39% (19.71%-64.19%) | 96% |  |
| **Study quality*** |  |  |  |  |  |
| General adults | 5 | 214906 | 68.82% (56.89%-79.64%) |  | - |
| High quality | 5 | - | - | - |  |
| Non-high quality | 0 | - | - | - |  |
| Adults with specific diseases | 7 | 3852 | 37.90% (27.46%-48.93%) |  | 0.59 |
| High quality | 3 | 2569 | 33.56% (19.10%-49.25%) | 98% |  |
| Non high quality | 4 | 1283 | 41.50% (21.61%-62.32%) | 98% |  |
| **Sample (Divided by median)**^#^ |  |  |  |  |  |
| General adults | 5 | 214906 | 68.82% (56.89%-79.64%) |  | <0.01 |
| Large sample | 4 | 213762 | 62.51% (49.10%-75.11%) | 100% |  |
| Small sample | 1 | 1144 | 89.99% (87.57%-91.14%) | - |  |
| Adults with specific diseases | 7 | 3852 | 37.90% (27.46%-48.93%) |  | 0.46 |
| Large sample | 2 | 2408 | 44.11% (31.27%-57.29%) | 98% |  |
| Small sample | 5 | 1444 | 35.38% (16.46%-55.92%) | 98% |  |

Note: *Studies with ≥7 low-risk items were considered high-quality.

^#^The total sample of the study, ≥1150 was considered large sample;＜1150 was considered small sample.
