# Supplementary material for: Contributions of 2‐h post‐load glucose, fasting blood glucose and glycosylated haemoglobin elevations to the prevalence of diabetes and pre‐diabetes in adults: A systematic analysis of global data
Source: Diabetes Obes Metab. 2025 Sep 15;27(12):7285–98. doi: 10.1111/dom.70130 (PMC12587253; doi:10.1111/dom.70130)
Supplement: Supplementary file 7 — Table S7. Characteristics of subgroup analyses—newly diagnosed diabetes by 2hPG criteria. [file DOM-27-7285-s004.docx]

**Supplementary Table 7 Characteristics of subgroup analyses**—**newly diagnosed diabetes by 2hPG criteria**

| **Subgroups** | **No. of studies** | **Newly identified diabetes** | **Proportion**  **（95% CI）** | **Heterogeneity**  **of subgroup**  **(I^2^)** | **Test for subgroup differences**  **(*P* value)** |
| --- | --- | --- | --- | --- | --- |
| **Study location** |  |  |  |  |  |
| General adults | 15 | 24214 | 68.40% (61.02%-75.34%) |  | 0.64 |
| Asian | 9 | 22160 | 66.15% (58.34%-73.56%) | 99% |  |
| Non-Asian | 6 | 2054 | 72.00% (55.24%-87.10%) | 98% |  |
| Adults with specific diseases | 9 | 2135 | 68.52% (55.16%-80.54%) |  | 0.92 |
| Asian | 4 | 873 | 69.60% (66.16%-72.28%) | 0% |  |
| Non-Asian | 5 | 1262 | 69.10% (44.02%-90.89%) | 94% |  |
| **Study Quality*** |  |  |  |  |  |
| General adults | 15 | 24214 | 68.40% (61.02%-75.34%) |  | 0.82 |
| High quality | 13 | 23853 | 68.96% (61.15%-76.29%) | 99% |  |
| Non-high quality | 2 | 361 | 64.39% (21.18%-100.0%) | 98% |  |
| Adults with specific diseases | 9 | 2135 | 68.52% (55.16%-80.54%) |  | 0.90 |
| High quality | 7 | 1927 | 68.92% (52.81%-83.32%) | 96% |  |
| Non-high quality | 2 | 208 | 67.96% (61.19%-73.90%) | 0% |  |
| **Sample (Divided by median)**^#^ |  |  |  |  |  |
| General adults | 15 | 24214 | 68.40% (61.02%-75.34%) |  | 0.91 |
| Large sample | 10 | 23753 | 67.88% (59.44%-75.83%) | 99% |  |
| Small sample | 5 | 461 | 69.48% (45.74%-90.53%) | 96% |  |
| Adults with specific diseases | 9 | 2135 | 68.52% (55.16%-80.54%) |  | 0.19 |
| Large sample | 2 | 1685 | 54.58% (26.13%-82.07%) | 99% |  |
| Small sample | 7 | 450 | 73.46% (66.33%-79.47%) | 46% |  |

Note: *Studies with ≥7 low-risk items were considered high-quality.

^#^The total sample of the study, ≥800 was considered large sample;＜800 was considered small sample.
