# Supplementary material for: Contributions of 2‐h post‐load glucose, fasting blood glucose and glycosylated haemoglobin elevations to the prevalence of diabetes and pre‐diabetes in adults: A systematic analysis of global data
Source: Diabetes Obes Metab. 2025 Sep 15;27(12):7285–98. doi: 10.1111/dom.70130 (PMC12587253; doi:10.1111/dom.70130)
Supplement: Supplementary file 8 — Table S8. Characteristics of subgroup analyses—newly diagnosed diabetes by FPG criteria. [file DOM-27-7285-s013.docx]

**Supplementary Table 8 Characteristics of subgroup analyses**—**newly diagnosed diabetes by FPG criteria**

| **Subgroups** | **No. of studies** | **Newly identified diabetes** | **Proportion**  **（95% CI）** | **Heterogeneity**  **of subgroup**  **(I^2^)** | **Test for subgroup differences**  **(*P* value)** |
| --- | --- | --- | --- | --- | --- |
| **Study location** |  |  |  |  |  |
| General adults | 15 | 24214 | 45.97% (41.01%-50.98%) |  | 0.77 |
| Asian | 9 | 22160 | 45.44% (39.45%-51.49%) | 98% |  |
| Non-Asian | 6 | 2054 | 47.07% (38.72%-55.46%) | 91% |  |
| Adults with specific diseases | 9 | 2135 | 40.06% (18.56%-63.57%) |  | 0.01 |
| Asian | 4 | 873 | 22.60% (15.08%-33.37%) | 85% |  |
| Non-Asian | 5 | 1262 | 51.58% (30.43%-72.45%) | 91% |  |
| **Study Quality*** |  |  |  |  |  |
| General adults | 15 | 24214 | 45.97% (41.01%-50.98%) |  | 0.62 |
| High quality | 13 | 23853 | 45.59% (40.29%-50.94%) | 97% |  |
| Non-high quality | 2 | 361 | 48.37% (41.17%-55.60%) | 46% |  |
| Adults with specific diseases | 9 | 2135 | 40.06% (18.56%-63.57%) |  | 0.56 |
| High quality | 7 | 1927 | 43.17% (18.88%-69.00%) | 99% |  |
| Non-high quality | 2 | 208 | 29.08% (00.00%-75.32%) | 91% |  |
| **Sample (Divided by median)**^#^ |  |  |  |  |  |
| General adults | 15 | 24214 | 45.97% (41.01%-50.98%) |  | 0.94 |
| Large sample^#^ | 10 | 23753 | 46.38% (41.16%-51.63%) | 97% |  |
| Small sample | 5 | 461 | 44.78% (19.75%-70.55%) | 97% |  |
| Adults with specific diseases | 9 | 2135 | 40.06% (18.56%-63.57%) |  | 0.58 |
| Large sample | 2 | 1685 | 50.27% (00.00%-99.99%) | 100% |  |
| Small sample | 7 | 450 | 35.21% (22.28%-50.17%) | 86% |  |

Note: *Studies with ≥7 low-risk items were considered high-quality.

^#^The total sample of the study, ≥800 was considered large sample;＜800 was considered small sample.
