# Supplementary material for: Contributions of 2‐h post‐load glucose, fasting blood glucose and glycosylated haemoglobin elevations to the prevalence of diabetes and pre‐diabetes in adults: A systematic analysis of global data
Source: Diabetes Obes Metab. 2025 Sep 15;27(12):7285–98. doi: 10.1111/dom.70130 (PMC12587253; doi:10.1111/dom.70130)
Supplement: Supplementary file 9 — Table S9. Characteristics of subgroup analyses—newly diagnosed diabetes by HbA1c criteria. [file DOM-27-7285-s020.docx]

**Supplementary Table 9 Characteristics of subgroup analyses**—**newly diagnosed diabetes by HbA1c criteria**

| **Subgroups** | **No. of studies** | **Newly identified diabetes** | **Proportion**  **（95% CI）** | **Heterogeneity**  **of subgroup**  **(I^2^)** | **Test for subgroup differences**  **(*P* value)** |
| --- | --- | --- | --- | --- | --- |
| **Study location** |  |  |  |  |  |
| General adults | 15 | 24214 | 60.88% (52.45%-69.00%) |  | 0.01 |
| Asian | 9 | 22160 | 70.39% (59.87%-80.13%) | 99% |  |
| Non-Asian | 6 | 2054 | 42.52% (30.64%-54.73%) | 96% |  |
| Adults with specific diseases | 9 | 2135 | 41.96% (20.90%-64.52%) |  | <0.01 |
| Asian | 4 | 873 | 62.83% (54.30%-70.02%) | 73% |  |
| Non-Asian | 5 | 1262 | 25.08% (10.40%-43.78%) | 89% |  |
| **Study Quality*** |  |  |  |  |  |
| General adults | 15 | 24214 | 60.88% (52.45%-69.00%) |  | 0.36 |
| High quality | 13 | 23853 | 62.43% (53.39%-71.09%) | 99% |  |
| Non-high quality | 2 | 361 | 49.21% (23.15%-75.40%) | 96% |  |
| Adults with specific diseases | 9 | 2135 | 41.96% (29.90%-64.52%) |  | 0.03 |
| High quality | 7 | 1927 | 36.31% (12.70%-63.10%) | 99% |  |
| Non-high quality | 2 | 208 | 63.99% (56.74%-69.83%) | 0% |  |
| **Sample (Divided by median)**^#^ |  |  |  |  |  |
| General adults | 15 | 24214 | 60.88% (52.45%-69.00%) |  | 0.01 |
| Large sample^#^ | 10 | 23753 | 53.48% (43.94%-62.90%) | 99% |  |
| Small sample | 5 | 461 | 78.09% (63.60%-90.67%) | 92% |  |
| Adults with specific diseases | 9 | 2135 | 41.96% (29.90%-64.52%) |  | 0.93 |
| Large sample | 2 | 1685 | 40.04% (00.00%-99.99%) | 100% |  |
| Small sample | 7 | 450 | 42.76% (26.64%-59.93%) | 90% |  |

Note: *Studies with ≥7 low-risk items were considered high-quality.

^#^The total sample of the study, ≥800 was considered large sample;＜800 was considered small sample.
