# Supplementary material for: Contributions of 2‐h post‐load glucose, fasting blood glucose and glycosylated haemoglobin elevations to the prevalence of diabetes and pre‐diabetes in adults: A systematic analysis of global data
Source: Diabetes Obes Metab. 2025 Sep 15;27(12):7285–98. doi: 10.1111/dom.70130 (PMC12587253; doi:10.1111/dom.70130)
Supplement: Supplementary file 10 — Table S10. Characteristics of subgroup analyses—newly diagnosed pre‐diabetes by 2hPG criteria. [file DOM-27-7285-s021.docx]

**Supplementary Table 10 Characteristics of subgroup analyses—newly diagnosed pre-diabetes by 2hPG criteria**

| **Subgroups** | **No. of studies** | **Newly identified pre-diabetes** | **Proportion**  **（95% CI）** | **Heterogeneity**  **of subgroup**  **(I^2^)** | **Test for subgroup differences**  **(*P* value)** |
| --- | --- | --- | --- | --- | --- |
| **Study location** |  |  |  |  |  |
| General adults | 5 | 133621 | 33.57% (21.44%-46.88%) |  | 0.02 |
| Asian | 3 | 126094 | 23.60% (09.82%-39.98%) | 100% |  |
| Non-Asian | 2 | 7527 | 49.83% (30.61%-69.07%) | 99% |  |
| Adults with specific diseases | 7 | 1687 | 47.38% (34.02%-60.92%) |  | 0.30 |
| Asian | 4 | 1039 | 51.98% (42.59%-61.26%) | 85% |  |
| Non-Asian | 3 | 648 | 41.28% (20.60%-63.09%) | 87% |  |
| **Study quality*** |  |  |  |  |  |
| General adults | 5 | 133621 | 33.57% (21.44%-46.88%) |  | - |
| High quality | 5 | - | - | - |  |
| Non-high quality | 0 | - | - | - |  |
| Adults with specific diseases | 7 | 1687 | 47.38% (34.02%-60.92%) |  | 0.84 |
| High quality | 3 | 1082 | 45.96% (16.94%-75.90%) | 99% |  |
| Non high quality | 4 | 605 | 48.24% (44.30%-52.25%) | 0% |  |
| **Sample (Divided by median)**^#^ |  |  |  |  |  |
| General adults | 5 | 133621 | 33.57% (21.44%-46.88%) |  | <0.01 |
| Large sample | 4 | 132598 | 27.50% (15.19%-41.58%) | 100% |  |
| Small sample | 1 | 1023 | 59.70% (56.50%-62.52%) | - |  |
| Adults with specific diseases | 7 | 1687 | 47.38% (34.02%-60.92%) |  | 0.79 |
| Large sample | 2 | 1059 | 43.32% (08.29%-80.84%) | 99% |  |
| Small sample | 5 | 628 | 48.38% (44.51%-52.32%) | 0% |  |

Note: *Studies with ≥7 low-risk items were considered high-quality.

^#^The total sample of the study, ≥1150 was considered large sample;＜1150 was considered small sample.
