# Supplementary material for: Contributions of 2‐h post‐load glucose, fasting blood glucose and glycosylated haemoglobin elevations to the prevalence of diabetes and pre‐diabetes in adults: A systematic analysis of global data
Source: Diabetes Obes Metab. 2025 Sep 15;27(12):7285–98. doi: 10.1111/dom.70130 (PMC12587253; doi:10.1111/dom.70130)
Supplement: Supplementary file 11 — Table S11. Characteristics of subgroup analyses—newly diagnosed pre‐diabetes by FPG criteria. [file DOM-27-7285-s018.docx]

**Supplementary Table 11 Characteristics of subgroup analyses—newly diagnosed pre-diabetes by FPG criteria**

| **Subgroups** | **No. of studies** | **Newly identified pre-diabetes** | **Proportion**  **（95% CI）** | **Heterogeneity**  **of subgroup**  **(I^2^)** | **Test for subgroup differences**  **(*P* value)** |
| --- | --- | --- | --- | --- | --- |
| **Study location** |  |  |  |  |  |
| General adults | 5 | 133621 | 51.21% (42.05%-60.33%) |  | 0.04 |
| Asian | 3 | 126094 | 37.64% (31.07%-44.54%) | 100% |  |
| Non-Asian | 2 | 7527 | 71.82% (56.04%-86.03%) | 99% |  |
| Adults with specific diseases | 7 | 1687 | 27.89% (18.49%-38.34%) |  | 0.39 |
| Asian | 4 | 1039 | 23.14% (11.18%-37.00%) | 95% |  |
| Non-Asian | 3 | 648 | 36.83% (09.17%-67.53%) | 93% |  |
| **Study quality*** |  |  |  |  |  |
| General adults | 5 | 133621 | 51.21% (42.05%-60.33%) |  | - |
| High quality | 5 | - | - | - |  |
| Non-high quality | 0 | - | - | - |  |
| Adults with specific diseases | 7 | 1687 | 27.89% (18.49%-38.34%) |  | 0.02 |
| High quality | 3 | 1082 | 14.77% (06.82%-24.64%) | 91% |  |
| Non-high quality | 4 | 605 | 42.00% (21.57%-63.35%) | 94% |  |
| **Sample (Divided by median)**^#^ |  |  |  |  |  |
| General adults | 5 | 133621 | 51.21% (42.05%-60.33%) |  | 0.30 |
| Large sample | 4 | 132598 | 48.05% (37.85%-58.33%) | 100% |  |
| Small sample | 1 | 1023 | 63.84% (60.76%-66.65%) | - |  |
| Adults with specific diseases | 7 | 1687 | 27.89% (18.49%-38.34%) |  | 0.40 |
| Large sample | 2 | 1059 | 20.44% (13.01%-29.17%) | 91% |  |
| Small sample | 5 | 628 | 31.29% (12.59%-52.18%) | 94% |  |

Note: *Studies with ≥7 low-risk items were considered high-quality.

^#^The total sample of the study, ≥1150 was considered large sample;＜1150 was considered small sample.
