# Supplementary material for: Contributions of 2‐h post‐load glucose, fasting blood glucose and glycosylated haemoglobin elevations to the prevalence of diabetes and pre‐diabetes in adults: A systematic analysis of global data
Source: Diabetes Obes Metab. 2025 Sep 15;27(12):7285–98. doi: 10.1111/dom.70130 (PMC12587253; doi:10.1111/dom.70130)
Supplement: Supplementary file 12 — Table S12. Characteristics of subgroup analyses—newly diagnosed pre‐diabetes by HbA1c criteria. [file DOM-27-7285-s006.docx]

**Supplementary Table 12 Characteristics of subgroup analyses**—**newly diagnosed pre-diabetes by HbA1c criteria**

| **Subgroups** | **No. of studies** | **Newly identified pre-diabetes** | **Proportion**  **（95% CI）** | **Heterogeneity**  **(I^2^)** | **Test for subgroup differences**  **(*P* value)** |
| --- | --- | --- | --- | --- | --- |
| **Study location** |  |  |  |  |  |
| General adults | 5 | 133621 | 67.17% (58.66%-75.16%) |  | <0.01 |
| Asian | 3 | 126094 | 81.22% (75.89%-85.81%) | 99% |  |
| Non-Asian | 2 | 7527 | 42.92% (25.98%-59.80%) | 98% |  |
| Adults with specific diseases | 7 | 1687 | 65.86% (51.93%-78.56%) |  | 0.83 |
| Asian | 4 | 1039 | 67.20% (44.64%-87.28%) | 98% |  |
| Non-Asian | 3 | 648 | 63.41% (36.42%-87.85%) | 92% |  |
| **Study quality*** |  |  |  |  |  |
| General adults | 5 | 133621 | 67.17% (58.66%-75.16%) |  | - |
| High quality | 5 | - | - | - |  |
| Non-high quality | 0 | - | - | - |  |
| Adults with specific diseases | 7 | 1687 | 65.86% (51.93%-78.56%) |  | 0.23 |
| High quality | 3 | 1082 | 74.49% (61.29%-85.80%) | 93% |  |
| Non-high quality | 4 | 605 | 58.21% (23.20%-90.61%) | 98% |  |
| **Sample (Divided by median)**^#^ |  |  |  |  |  |
| General adults | 5 | 133621 | 67.17% (58.66%-75.16%) |  | <0.01 |
| Large sample | 4 | 132598 | 74.66% (66.71%-81.86%) | 100% |  |
| Small sample | 1 | 1023 | 34.10% (31.33%-37.15%) | - |  |
| Adults with specific diseases | 7 | 1687 | 65.86% (51.93%-78.56%) |  | 0.29 |
| Large sample | 2 | 1059 | 74.79% (59.13%-88.13%) | 97% |  |
| Small sample | 5 | 628 | 61.25% (31.80%-88.29%) | 97% |  |

Note: *Studies with ≥7 low-risk items were considered high-quality.

^#^The total sample of the study, ≥1150 was considered large sample;＜1150 was considered small sample.
