# Supplementary material for: Contributions of 2‐h post‐load glucose, fasting blood glucose and glycosylated haemoglobin elevations to the prevalence of diabetes and pre‐diabetes in adults: A systematic analysis of global data
Source: Diabetes Obes Metab. 2025 Sep 15;27(12):7285–98. doi: 10.1111/dom.70130 (PMC12587253; doi:10.1111/dom.70130)
Supplement: Supplementary file 13 — Figure S1. Forest plot of the proportions of each combination of 2‐h post‐load glucose, fasting plasma glucose and glycosylated haemoglobin among adult participants newly diagnosed with diabetes. (A) The general population; (B)the population with specific diseases. [file DOM-27-7285-s017.pdf]

(A) the general population

a. normal 2hPG and HbA1c but elevated FPG(isolated FPG elevation)

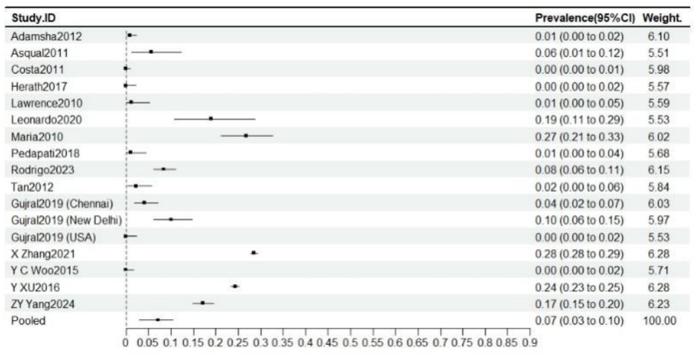

e. normal 2hPG but elevated FPG and HbA1c

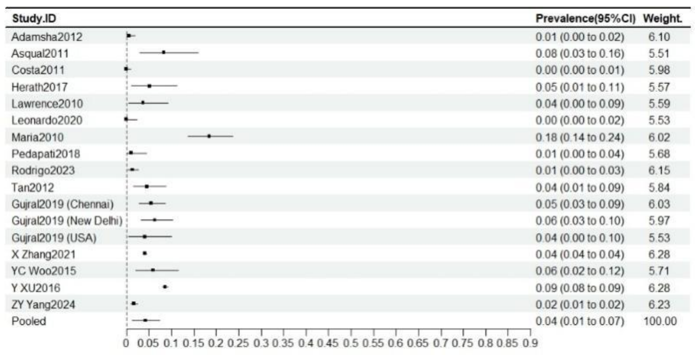

b. normal FPG and HbA1c but elevated 2hPG (isolated 2hPG elevation)

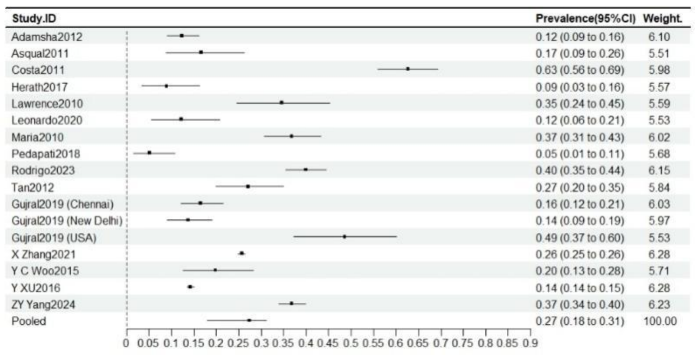

f. normal FPG but elevated 2hPG and HbA1c

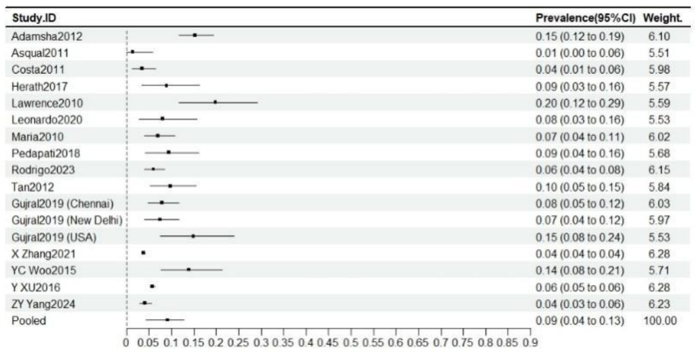

c. normal FPG and 2hPG but elevated HbA1c(isolated HbA1c elevation)

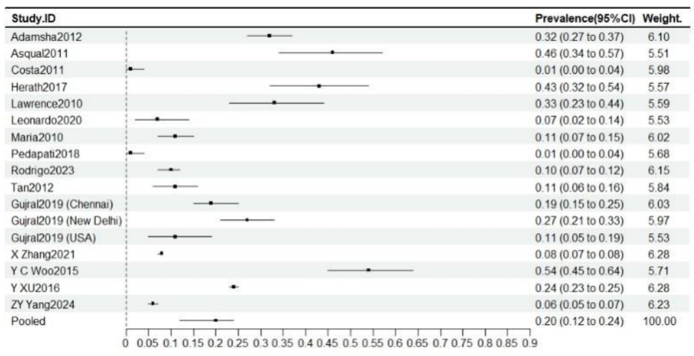

g. elevated FPG, 2hPG and HbA1c

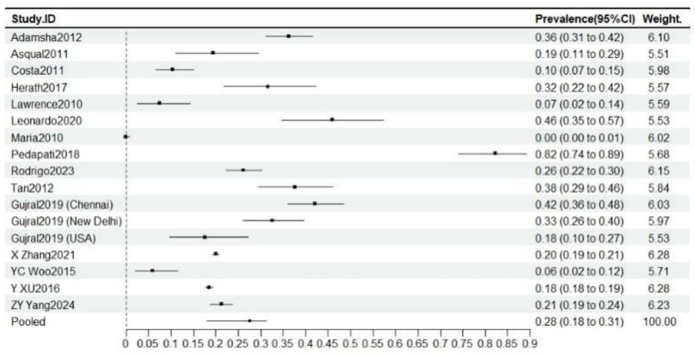

d. normal HbA1c but elevated FPG and 2hPG

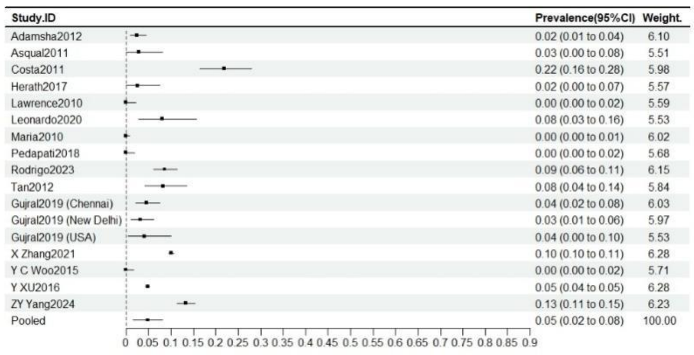

Statistics:

I-squared(95%CI): 98.900(98.679 - 99.083)

Cochran's Q: 1453.926

Chi2, p: 0

tau2: 0.098

FPG. (a+d+e+g)

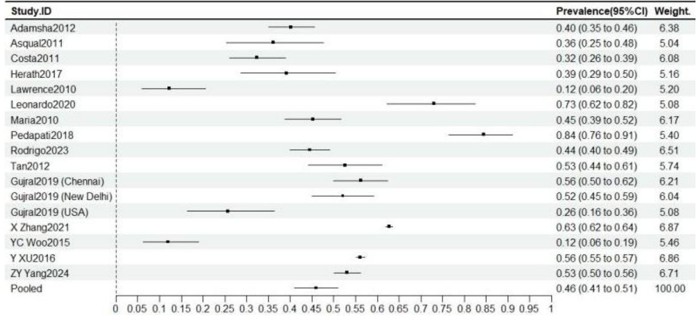

Statistics:

I-squared(95%CI): 97.209 (96.424 - 97.822)

Cochran's Q: 573.294

Chi2, p: 0

tau2: 0.038

2hPG. (b+d+f+g)

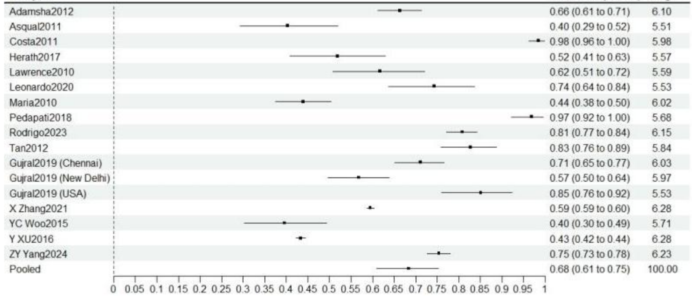

Statistics:

I-squared(95%CI): 98.900(98.678 - 99.083)

Cochran's Q: 1452.752

Chi2, p: 0

tau2: 0.098

HbA1c. (c+e+f+g)

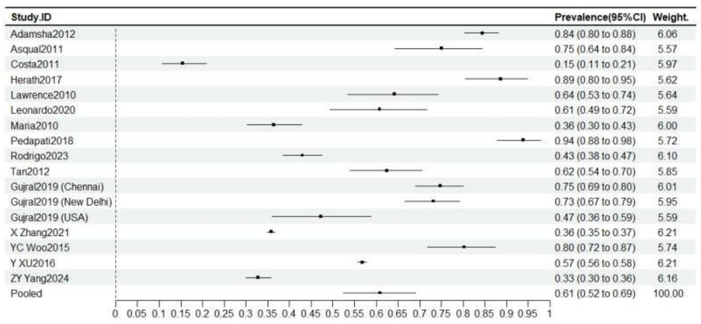

Statistics:

I-squared(95%CI): 99.102(98.934 - 99.243)

Cochran's Q: 1780.945

Chi2, p: 0

tau2: 0.120

(B) the population with specific diseases

a. normal 2hPG and HbA1c but elevated FPG (isolated FPG elevation)

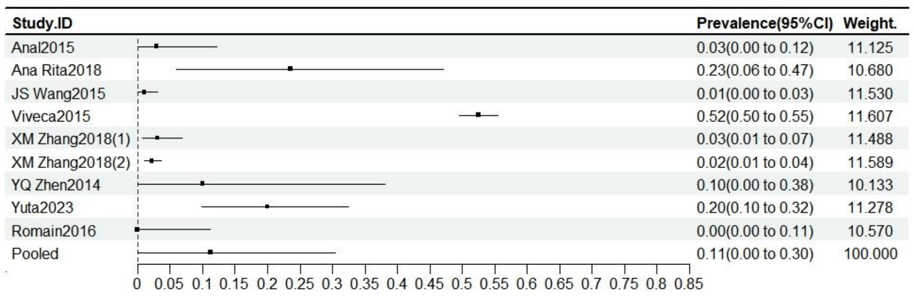

e. normal 2hPG but elevated FPG and HbA1c

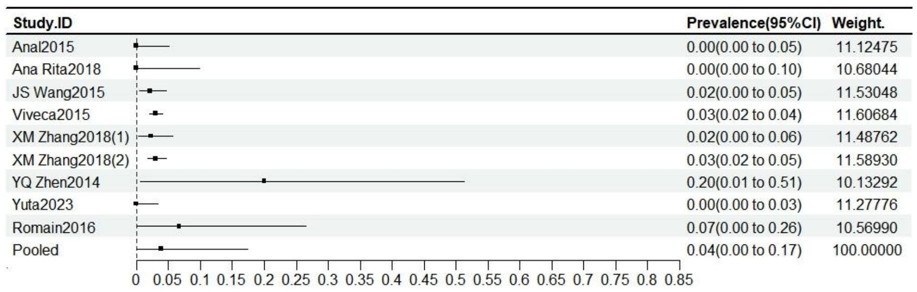

b. normal FPG and HbA1c but elevated 2hPG (isolated 2hPG elevation)

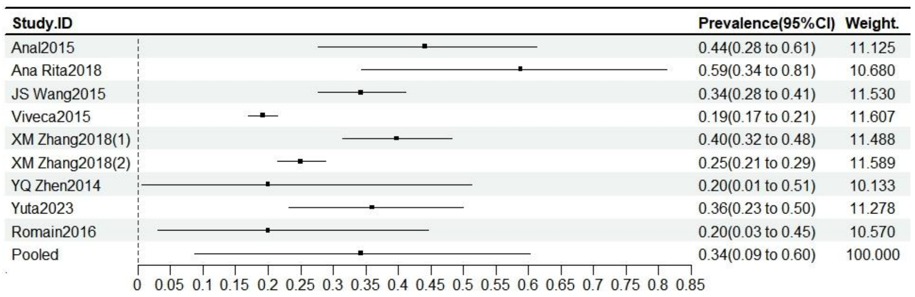

f. normal FPG but elevated 2hPG and HbA1c

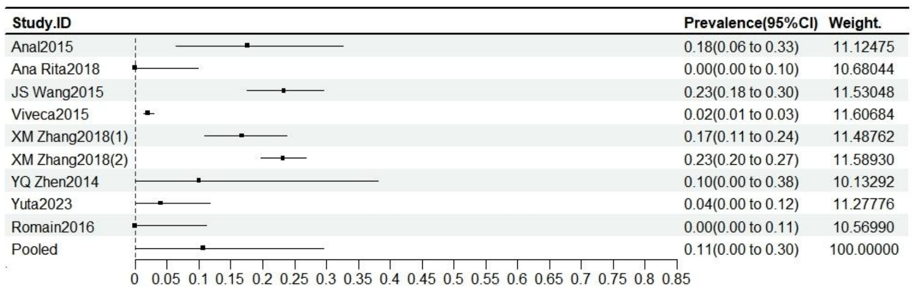

c. normal FPG and 2hPG but elevated HbA1c (isolated HbA1c elevation)

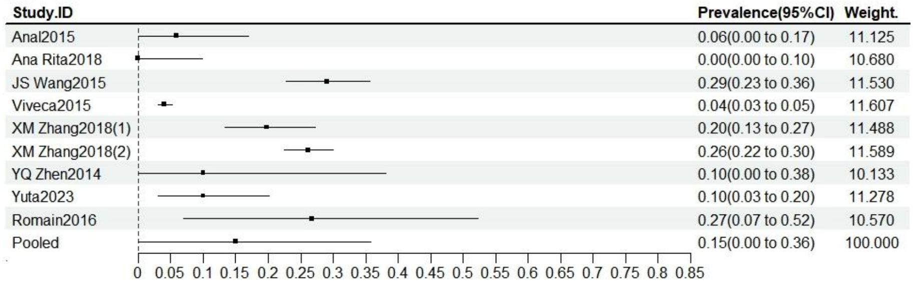

g. elevated FPG, 2hPG and HbA1c

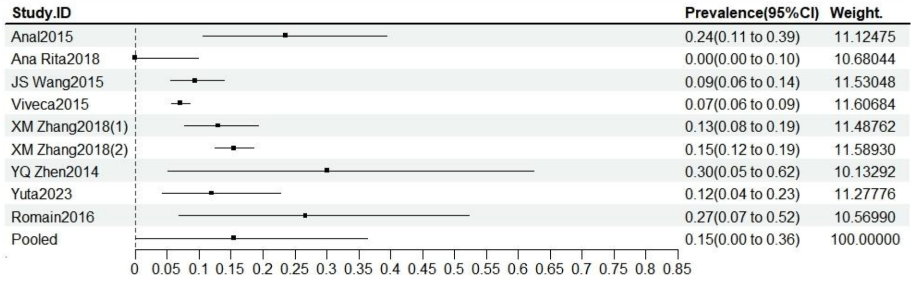

d. normal HbA1c but elevated FPG and 2hPG

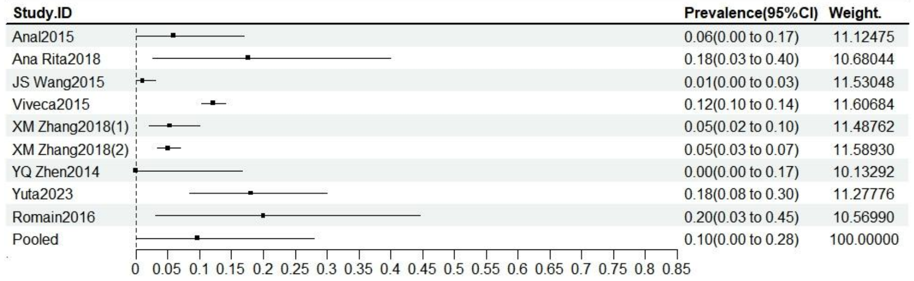

Statistics:
I-squared(95%CI): 99.101 (98.856 - 99.294)
Cochran's Q: 890.126
Chi2, p: 0
tau2: 0.648

FPG. (a+d+e+g)

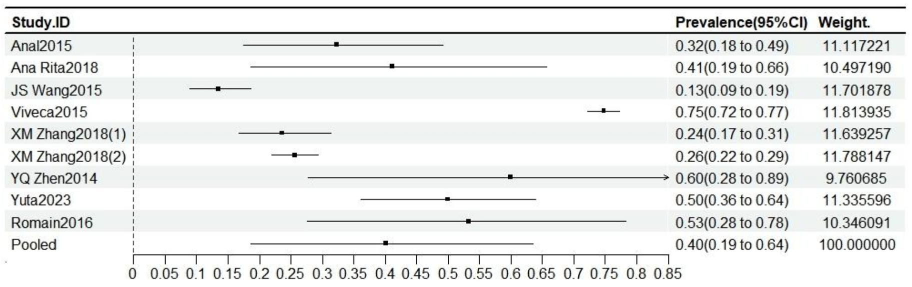

Statistics:
I-squared(95%CI): 98.705 (98.299 - 99.013)
Cochran's Q: 617.547
Chi2, p: 0
tau2: 0.448

2hPG. (b+d+f+g)

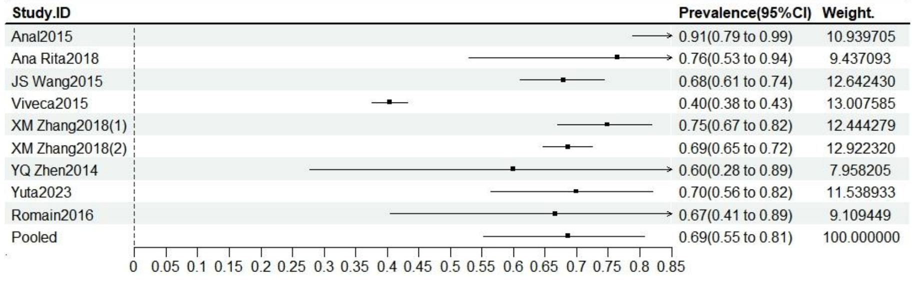

Statistics:
I-squared(95%CI): 96.178 (94.379 - 97.401)
Cochran's Q: 209.297
Chi2, p: 0
tau2: 0.148

HbA1c. (c+e+f+g)

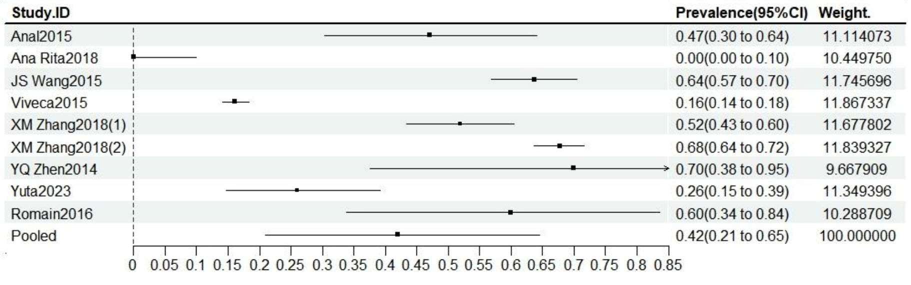

Statistics:
I-squared(95%CI): 98.601 (98.149 - 98.942)
Cochran's Q: 571.538
Chi2, p: 0
tau2: 0.414

Supplementary Figure 1. Forest plot of the proportions of each combination of 2-hour post-load glucose, fasting plasma glucose, and glycated hemoglobin among adult participants newly diagnosed with diabetes. (A) the general population; (B)the population with specific diseases
