# Supplementary material for: Contributions of 2‐h post‐load glucose, fasting blood glucose and glycosylated haemoglobin elevations to the prevalence of diabetes and pre‐diabetes in adults: A systematic analysis of global data
Source: Diabetes Obes Metab. 2025 Sep 15;27(12):7285–98. doi: 10.1111/dom.70130 (PMC12587253; doi:10.1111/dom.70130)
Supplement: Supplementary file 14 — Figure S2. Forest plot of the proportions of each combination of 2‐h post‐load glucose, fasting plasma glucose and glycosylated haemoglobin among adult participants newly diagnosed with pre‐diabetes. (A) The general population; (B) the population with specific diseases. [file DOM-27-7285-s014.pdf]

(A) the general population

a. normal 2hPG and HbA1c but elevated FPG (isolated FPG elevation)

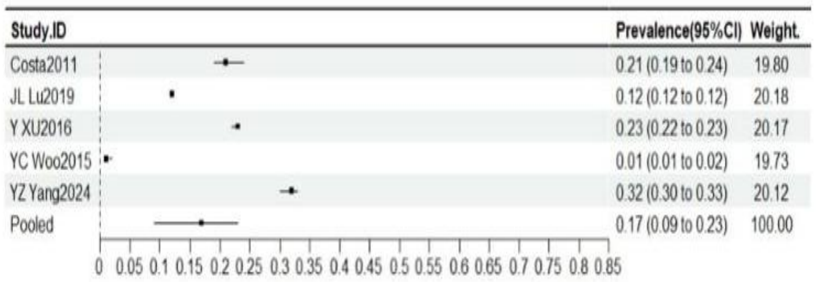

e. normal 2hPG but elevated FPG and HbA1c

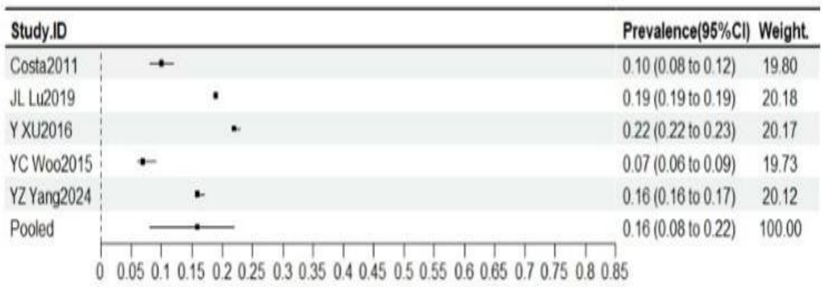

b. normal FPG and HbA1c but elevated 2hPG (isolated 2hPG elevation)

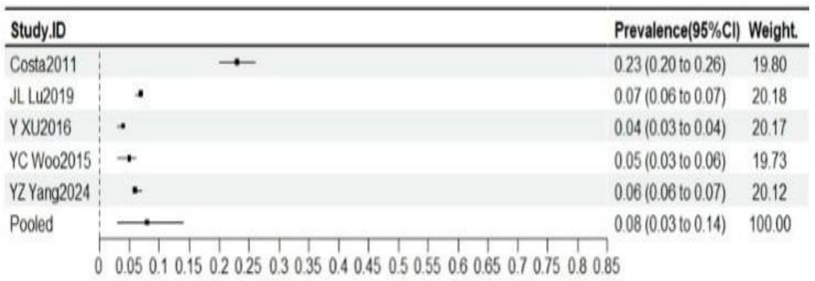

f. normal FPG but elevated 2hPG and HbA1c

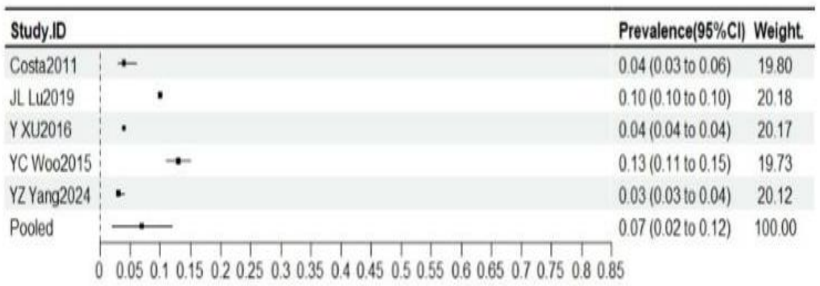

c. normal FPG and 2hPG but elevated HbA1c (isolated HbA1c elevation)

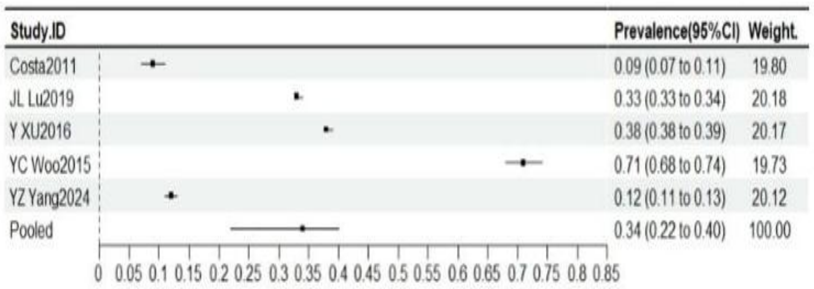

g. elevated FPG, 2hPG and HbA1c

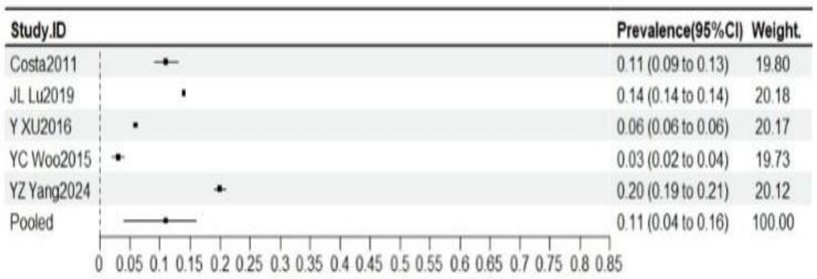

d. normal HbA1c but elevated FPG and 2hPG

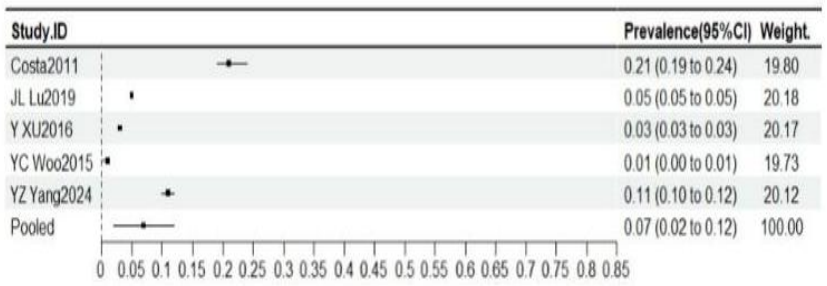

Statistics:
I-squared(95%CI): 99.890(99.891 - 99.907)
Cochran's Q: 3651.552
Chi2, p: 0
tau2: 0.051

FPG. (a+d+e+g)

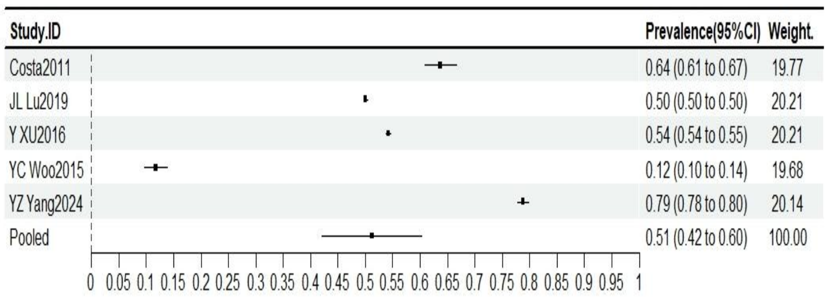

Statistics:
I-squared(95%CI): 99.870(99.846 - 99.891)
Cochran's Q: 3080.445
Chi2, p: 0
tau2: 0.043

2hPG. (b+d+f+g)

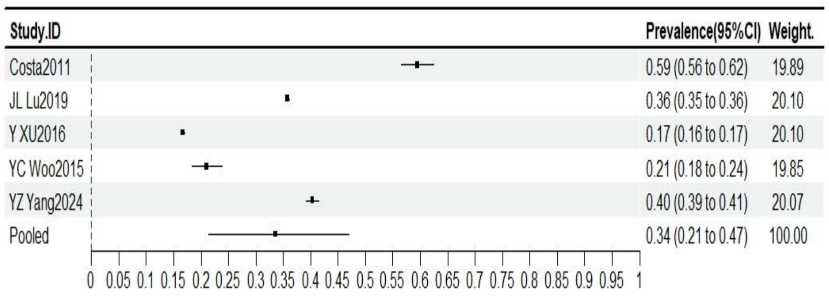

Statistics:
I-squared(95%CI): 99.940(99.931 - 99.947)
Cochran's Q: 6641.740
Chi2, p: 0
tau2: 0.092

HbA1c. (c+e+f+g)

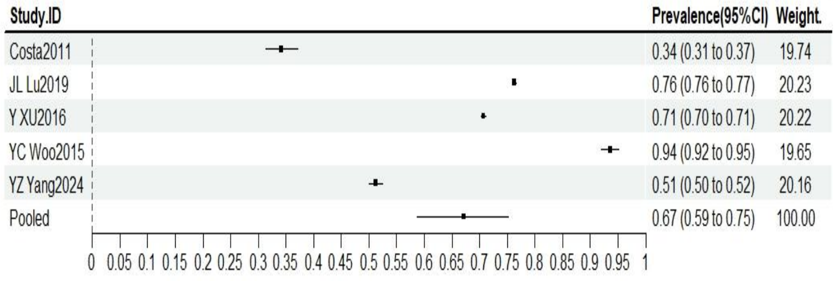

Statistics:
I-squared(95%CI): 99.859(99.832 - 99.882)
Cochran's Q: 2834.263
Chi2, p: 0
tau2: 0.039

(B) the population with specific diseases

a. normal 2hPG and HbA1c but elevated FPG (isolated FPG elevation)

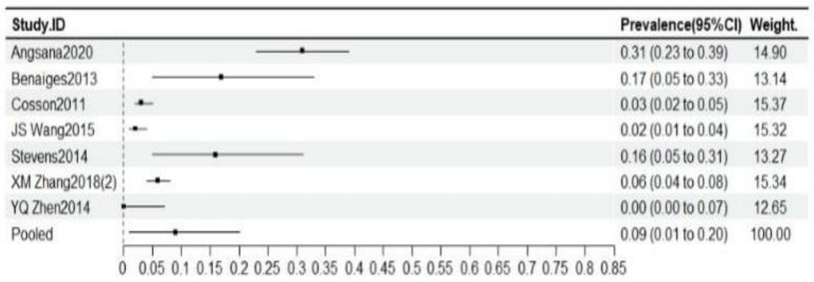

e. normal 2hPG but elevated FPG and HbA1c

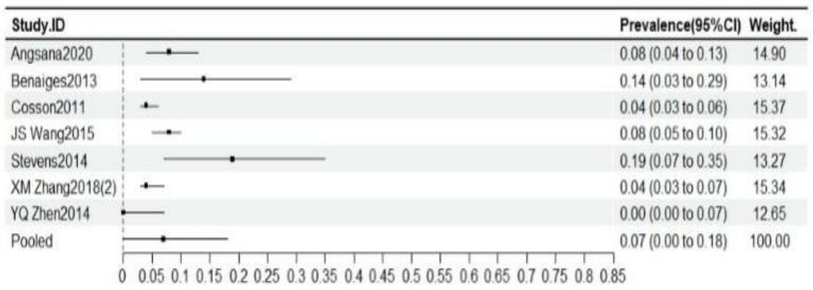

b. normal FPG and HbA1c but elevated 2hPG (isolated 2hPG elevation)

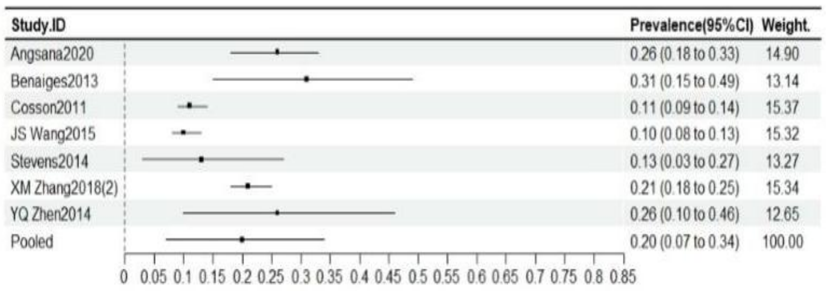

f. normal FPG but elevated 2hPG and HbA1c

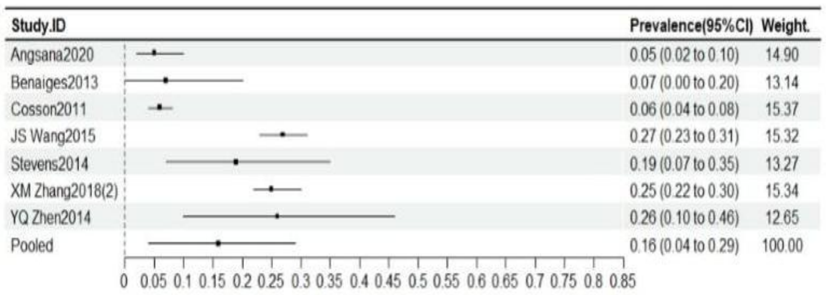

c. normal FPG and 2hPG but elevated HbA1c (isolated HbA1c elevation)

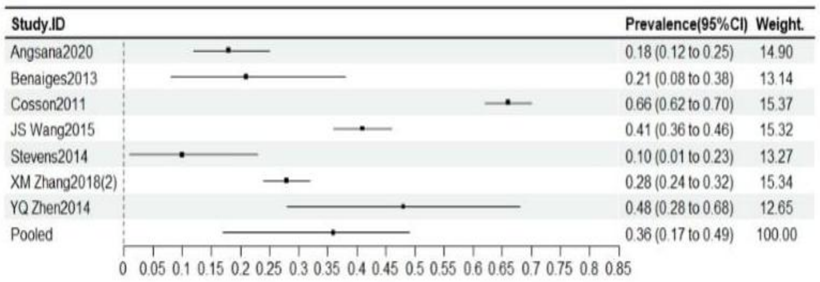

g. elevated FPG, 2hPG and HbA1c

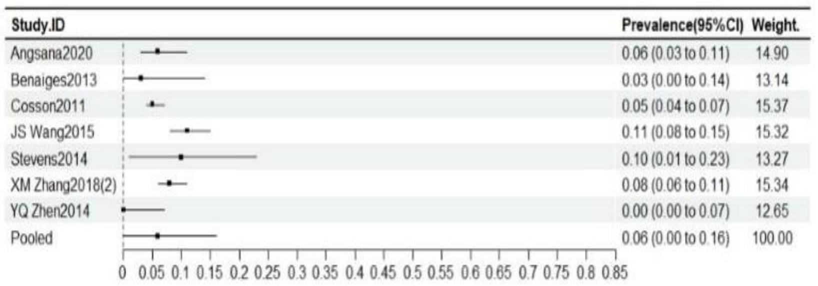

d. normal HbA1c but elevated FPG and 2hPG

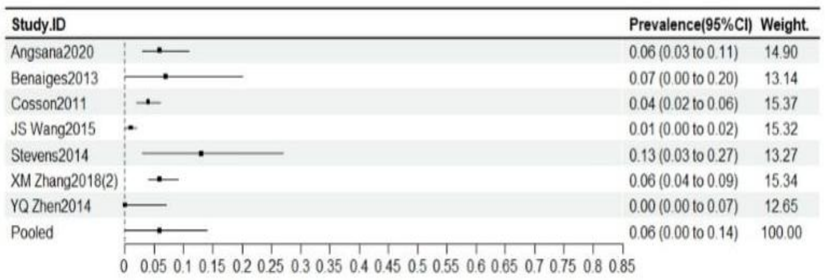

Statistics:
I-squared(95%CI): 97.490(96.295 - 98.299)
Cochran's Q: 2239.032
Chi2, p: 0
tau2: 0.188

FPG. (a+d+e+g)

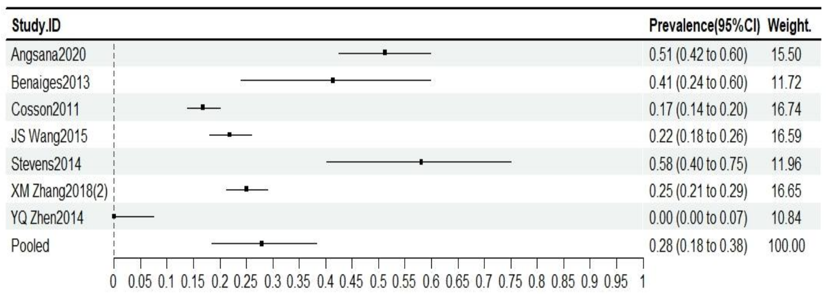

Statistics:
I-squared(95%CI): 93.815(89.659 - 96.301)
Cochran's Q: 97.008
Chi2, p: 0
tau2: 0.073

2hPG. (b+d+f+g)

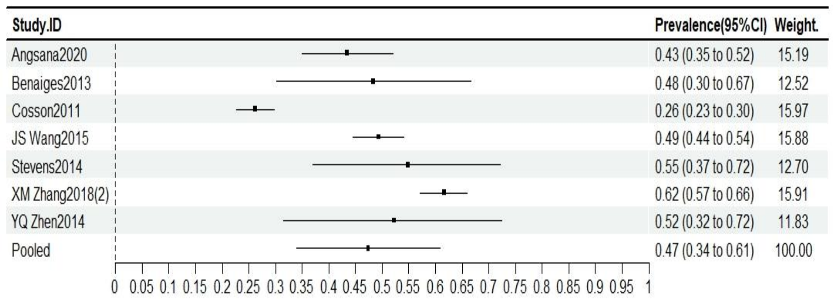

Statistics:
I-squared(95%CI): 95.963(93.658 - 97.431)
Cochran's Q: 148.638
Chi2, p: 0
tau2: 0.115

HbA1c. (c+e+f+g)

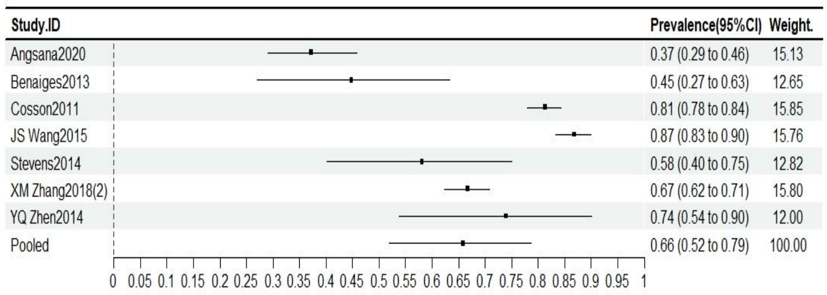

Statistics:
I-squared(95%CI): 96.289(94.236 - 97.610)
Cochran's Q: 161.675
Chi2, p: 0
tau2: 0.126

Supplementary Figure 2. Forest plot of the proportions of each combination of 2-hour post-load glucose, fasting plasma glucose, and glycated hemoglobin among adult participants newly diagnosed with pre-diabetes.
(A) the general population; (B) the population with specific diseases
