# Supplementary material for: Contributions of 2‐h post‐load glucose, fasting blood glucose and glycosylated haemoglobin elevations to the prevalence of diabetes and pre‐diabetes in adults: A systematic analysis of global data
Source: Diabetes Obes Metab. 2025 Sep 15;27(12):7285–98. doi: 10.1111/dom.70130 (PMC12587253; doi:10.1111/dom.70130)
Supplement: Supplementary file 15 — Figure S3. Sensitivity analyses (retaining only studies with nationally or regionally representative samples)—The proportions of different combinations of 2‐h post‐load glucose, fasting plasma glucose and glycoslyated haemoglobin among adult participants newly diagnosed with diabetes. (A) The general population; (B) the population with specific diseases. [file DOM-27-7285-s005.pdf]

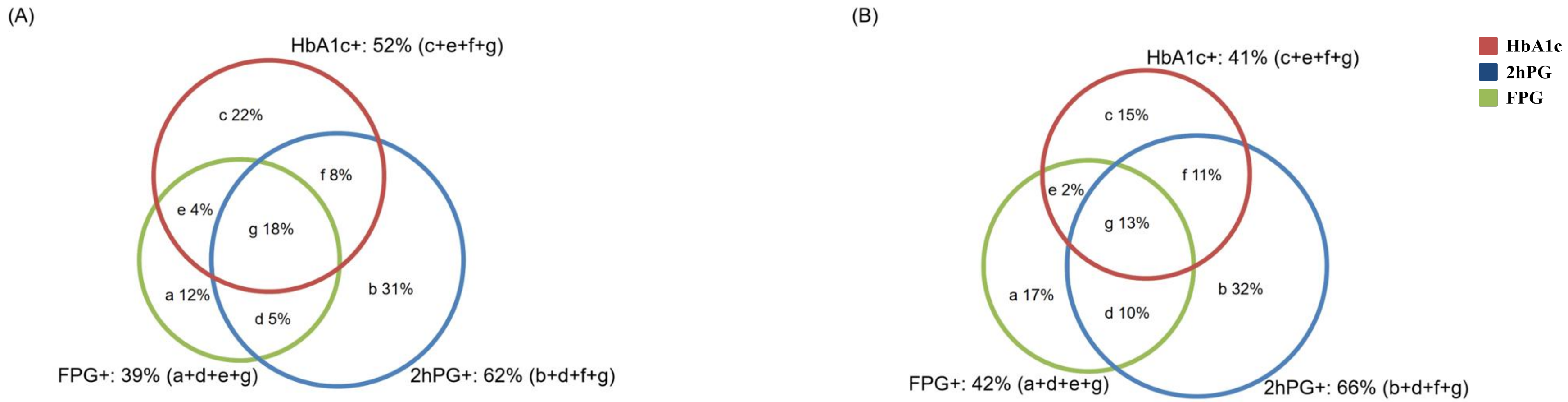

Supplementary Figure 3. Sensitivity analyses (retaining only studies with nationally or regionally representative samples)—The proportions of different combinations of 2-hour post-load glucose, fasting plasma glucose, and glycated hemoglobin among adult participants newly diagnosed with diabetes. (A) the general population; (B) the population with specific diseases

Abbreviations: FPG: fasting blood glucose; 2hPG: 2-hour post-load glucose; HbA1c: glycosylated hemoglobin
