# Supplementary material for: Contributions of 2‐h post‐load glucose, fasting blood glucose and glycosylated haemoglobin elevations to the prevalence of diabetes and pre‐diabetes in adults: A systematic analysis of global data
Source: Diabetes Obes Metab. 2025 Sep 15;27(12):7285–98. doi: 10.1111/dom.70130 (PMC12587253; doi:10.1111/dom.70130)
Supplement: Supplementary file 16 — Figure S4. Sensitivity analyses (retaining only studies with nationally or regionally representative samples)—the proportions of different combinations of 2‐h post‐load glucose, fasting plasma glucose and glycosylated haemoglobin among general population newly diagnosed with pre‐diabetes. [file DOM-27-7285-s026.pdf]

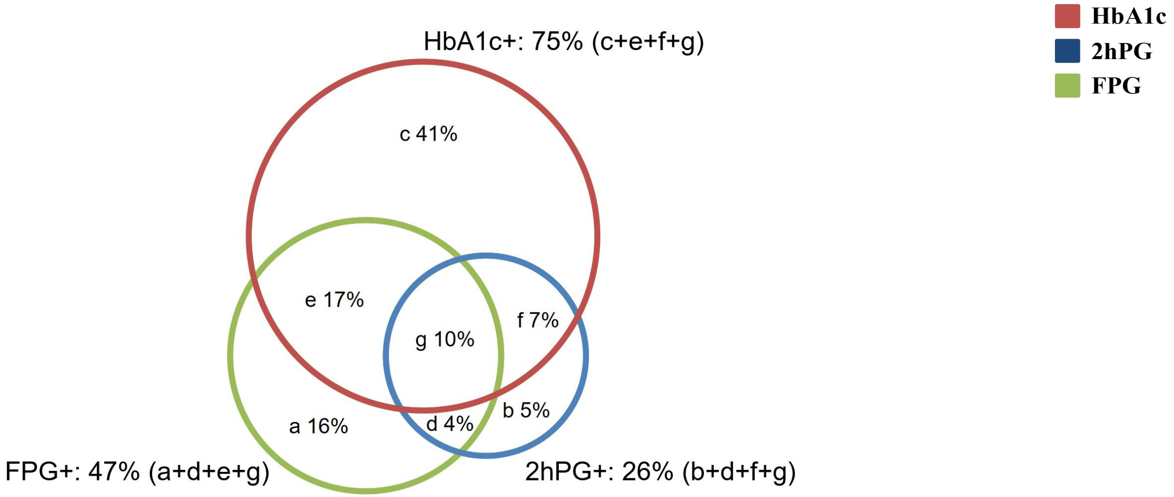

Supplementary Figure 4. Sensitivity analyses (retaining only studies with nationally or regionally representative samples)—The proportions of different combinations of 2-hour post-load glucose, fasting plasma glucose, and glycated hemoglobin among general population newly diagnosed with pre-diabetes

Abbreviations: FPG: fasting blood glucose; 2hPG: 2-hour post-load glucose; HbA1c: glycosylated hemoglobin
