# Supplementary material for: Contributions of 2‐h post‐load glucose, fasting blood glucose and glycosylated haemoglobin elevations to the prevalence of diabetes and pre‐diabetes in adults: A systematic analysis of global data
Source: Diabetes Obes Metab. 2025 Sep 15;27(12):7285–98. doi: 10.1111/dom.70130 (PMC12587253; doi:10.1111/dom.70130)
Supplement: Supplementary file 17 — Figure S5. Sensitivity analyses (retaining only studies with nationally or regionally representative samples)—forest plot of the proportions of each combination of 2‐h post‐load glucose, fasting plasma glucose and glycosylated haemoglobin among adult participants newly diagnosed with diabetes. (A) The general population; (B) the population with specific diseases. [file DOM-27-7285-s009.pdf]

(A) the general population

a. normal 2hPG and HbA1c but elevated FPG (isolated FPG elevation)

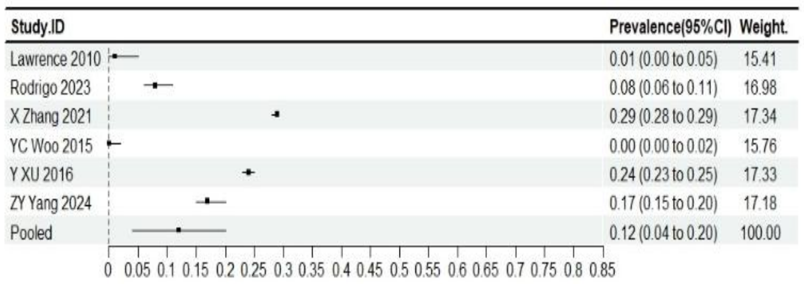

e. normal 2hPG but elevated FPG and HbA1c

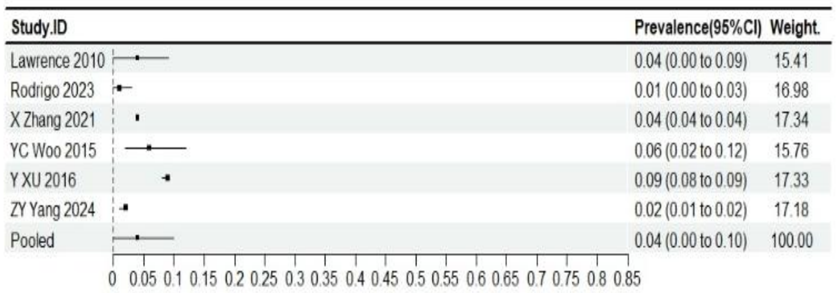

b. normal FPG and HbA1c but elevated 2hPG (isolated 2hPG elevation)

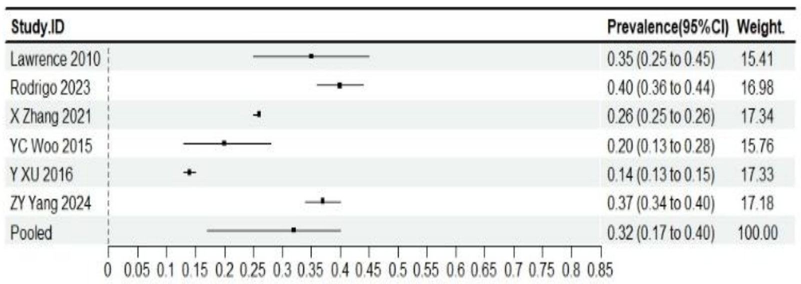

f. normal FPG but elevated 2hPG and HbA1c

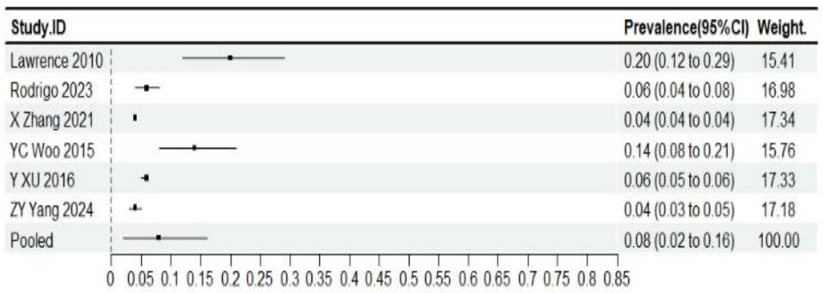

c. normal FPG and 2hPG but elevated HbA1c (isolated HbA1c elevation)

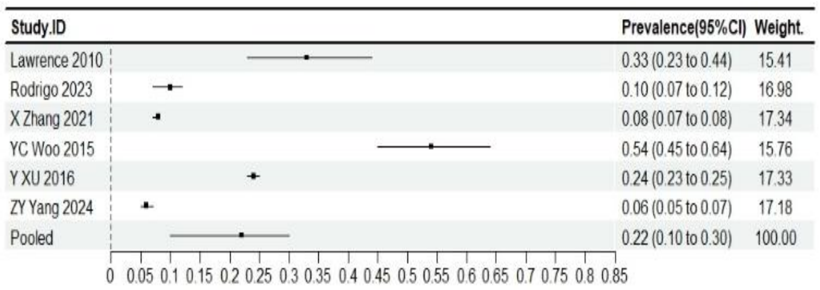

g. elevated FPG, 2hPG and HbA1c

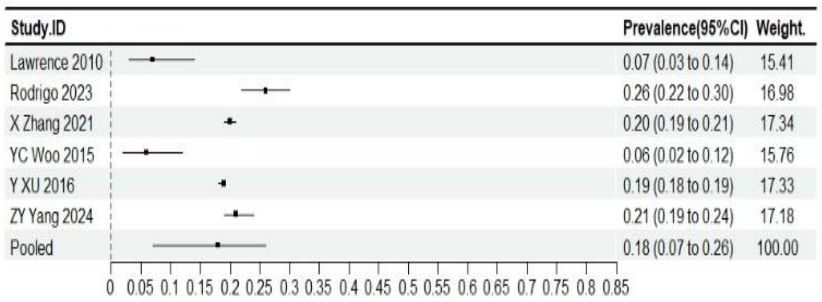

d. normal HbA1c but elevated FPG and 2hPG

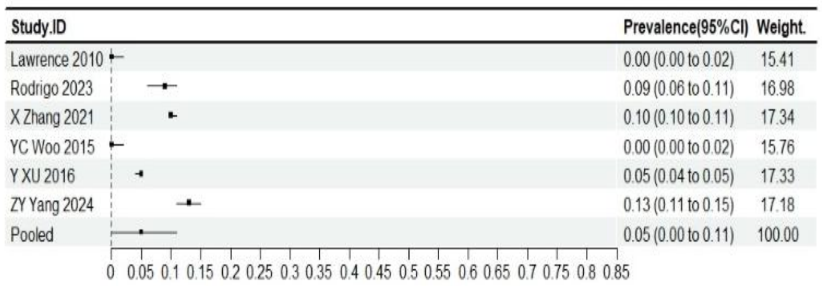

Statistics:
I-squared(95%CI): 99.582 (99.472 - 99.669)
Cochran's Q: 1197.198
Chi2, p: 0
tau2: 0.097

FPG. (a+d+e+g)

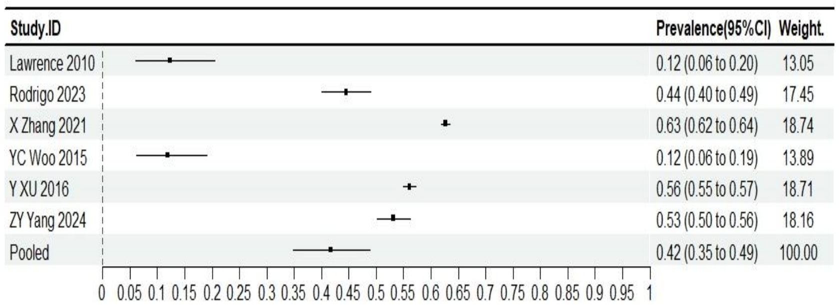

Statistics:
I-squared(95%CI): 98.562 (97.948 - 98.993)
Cochran's Q: 347.816
Chi2, p: 0
tau2: 0.028

2hPG. (b+d+f+g)

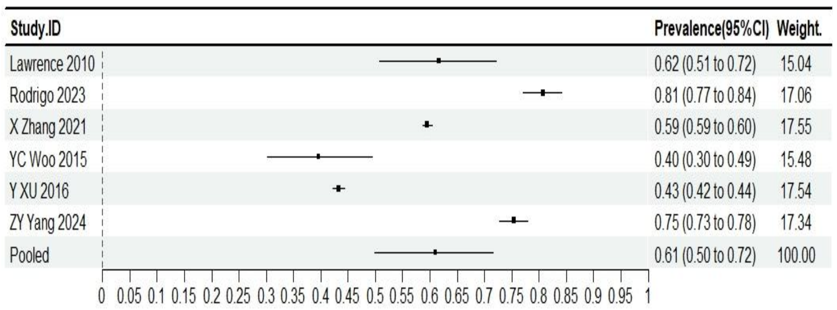

Statistics:
I-squared(95%CI): 99.445 (99.281 - 99.571)
Cochran's Q: 900.454
Chi2, p: 0
tau2: 0.073

HbA1c. (c+e+f+g)

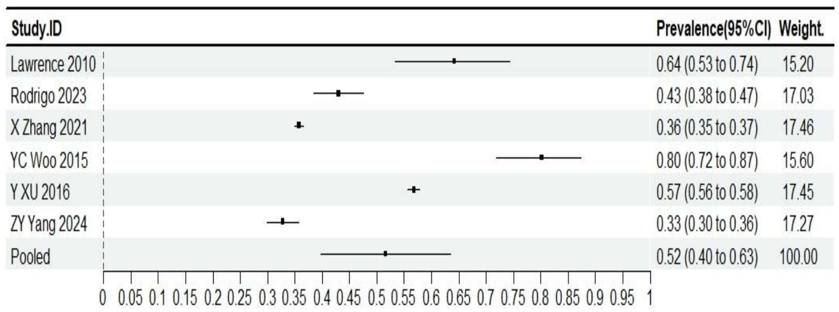

Statistics:
I-squared(95%CI): 99.506 (99.368 - 99.615)
Cochran's Q: 1012.845
Chi2, p: 0
tau2: 0.082

(B) the population with specific diseases

a. normal 2hPG and HbA1c but elevated FPG (isolated FPG elevation)

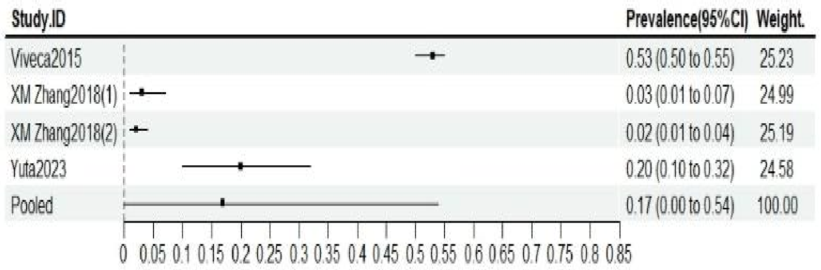

e. normal 2hPG but elevated FPG and HbA1c

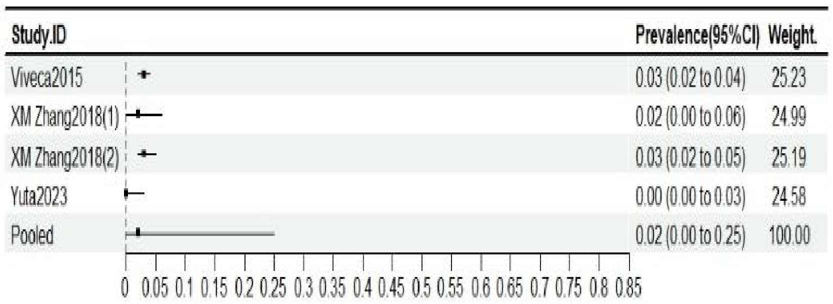

b. normal FPG and HbA1c but elevated 2hPG (isolated 2hPG elevation)

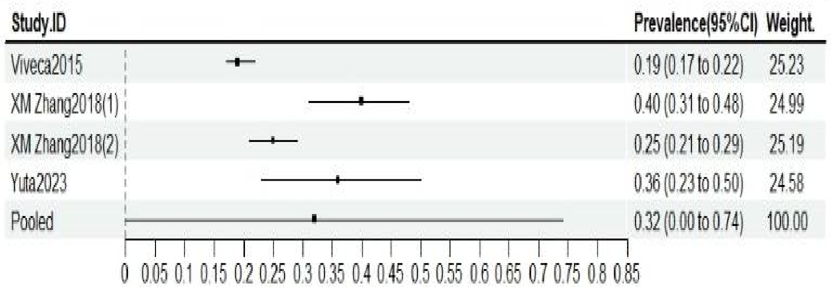

f. normal FPG but elevated 2hPG and HbA1c

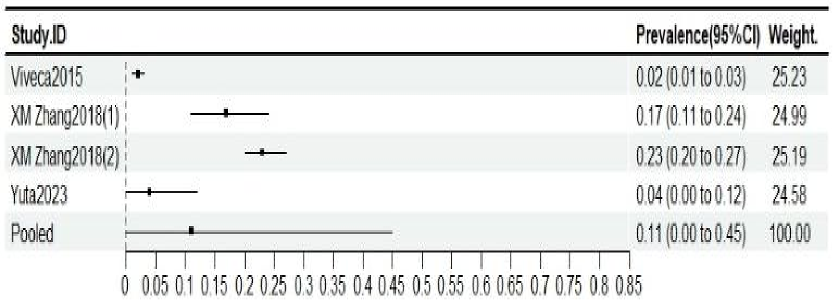

c. normal FPG and 2hPG but elevated HbA1c (isolated HbA1c elevation)

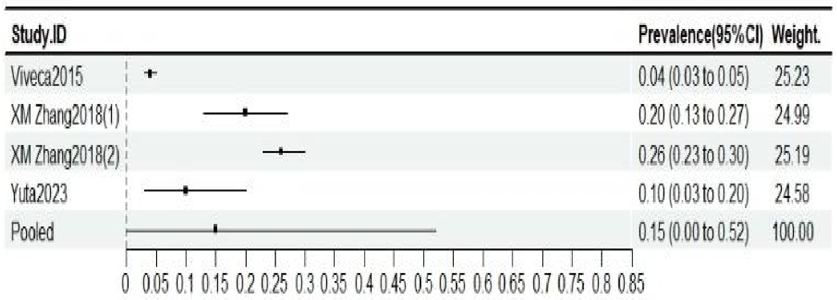

g. elevated FPG, 2hPG and HbA1c

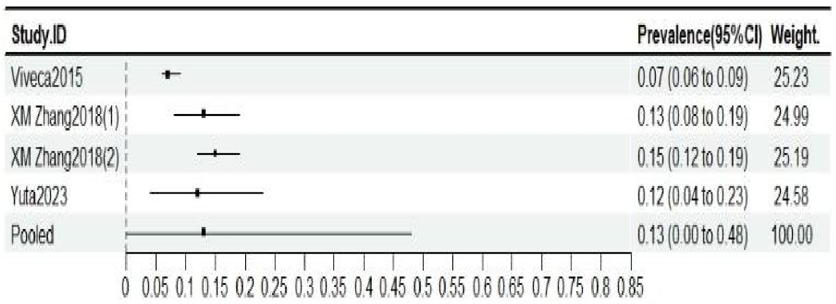

d. normal HbA1c but elevated FPG and 2hPG

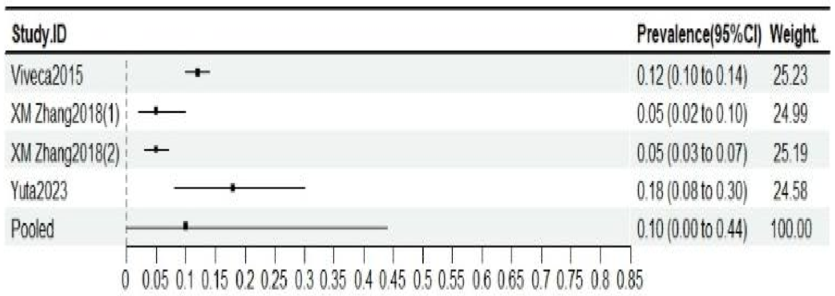

Statistics:
I-squared(95%CI): 99.584 (99.438 - 99.692)
Cochran's Q: 721.172
Chi2, p: 0
tau2: 0.720

FPG. (a+d+e+g)

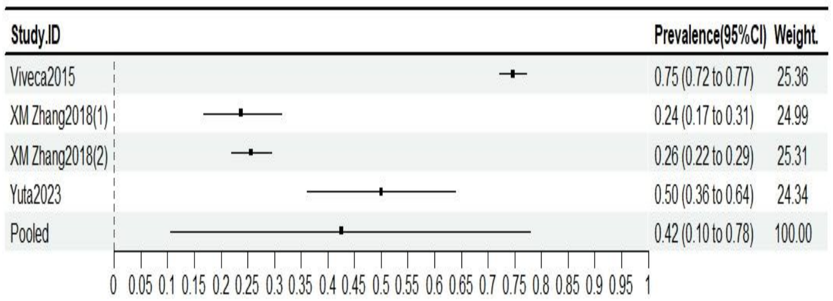

Statistics:
I-squared(95%CI): 99.339 (99.059 - 99.535)
Cochran's Q: 453.766
Chi2, p: 0
tau2: 0.452

2hPG. (b+d+f+g)

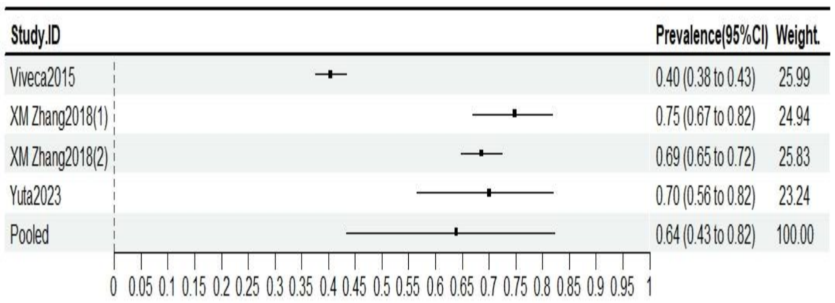

Statistics:
I-squared(95%CI): 99.142 (96.947 - 98.869)
Cochran's Q: 161.430
Chi2, p: 0
tau2: 0.159

HbA1c. (c+e+f+g)

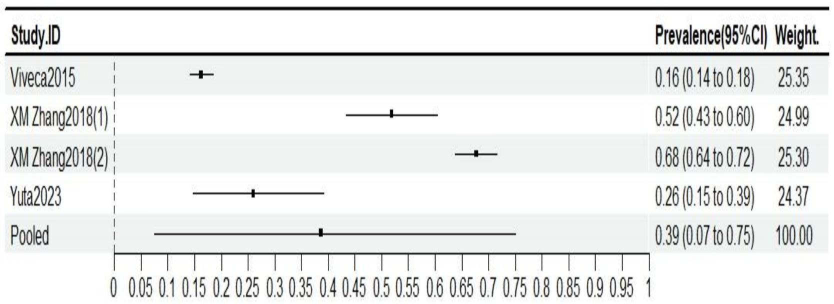

Statistics:
I-squared(95%CI): 99.362 (99.096 - 99.550)
Cochran's Q: 470.388
Chi2, p: 0
tau2: 0.468

Supplementary Figure 5. Sensitivity analyses (retaining only studies with nationally or regionally representative samples)—Forest plot of the proportions of each combination of 2-hour post-load glucose, fasting plasma glucose, and glycated hemoglobin among adult participants newly diagnosed with diabetes. (A) the general population; (B) the population with specific diseases
