# Supplementary material for: Contributions of 2‐h post‐load glucose, fasting blood glucose and glycosylated haemoglobin elevations to the prevalence of diabetes and pre‐diabetes in adults: A systematic analysis of global data
Source: Diabetes Obes Metab. 2025 Sep 15;27(12):7285–98. doi: 10.1111/dom.70130 (PMC12587253; doi:10.1111/dom.70130)
Supplement: Supplementary file 18 — Figure S6. Sensitivity analyses (retaining only studies with nationally or regionally representative samples)—forest plot of the proportions of each combination of 2‐h post‐load glucose, fasting plasma glucose and glycosylated haemoglobin among general population newly diagnosed with pre‐diabetes. [file DOM-27-7285-s001.pdf]

the general population

a. normal 2hPG and HbA1c but elevated FPG (isolated FPG elevation)

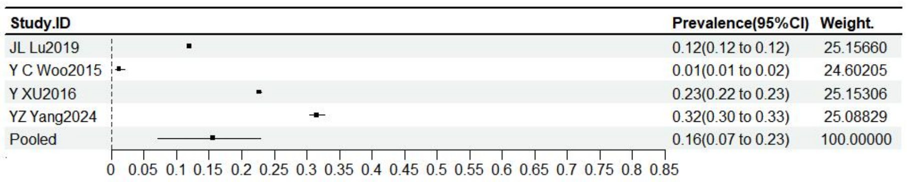

e. normal 2hPG but elevated FPG and HbA1c

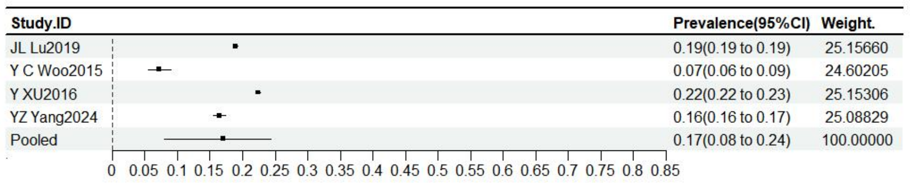

b. normal FPG and HbA1c but elevated 2hPG (isolated 2hPG elevation)

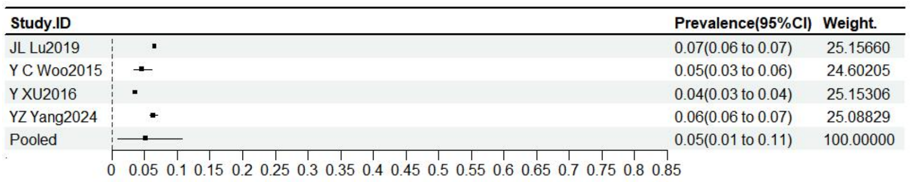

f. normal FPG but elevated 2hPG and HbA1c

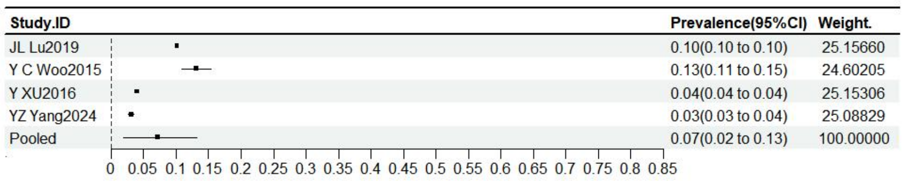

c. normal FPG and 2hPG but elevated HbA1cn (isolated HbA1c elevation)

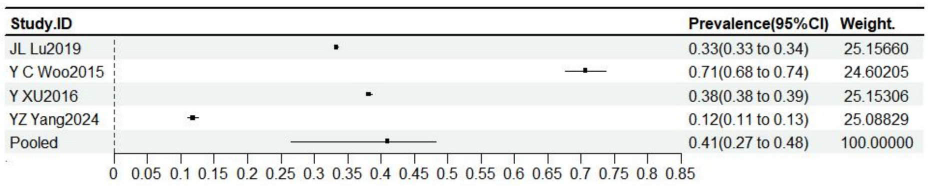

g. elevated FPG, 2hPG and HbA1c

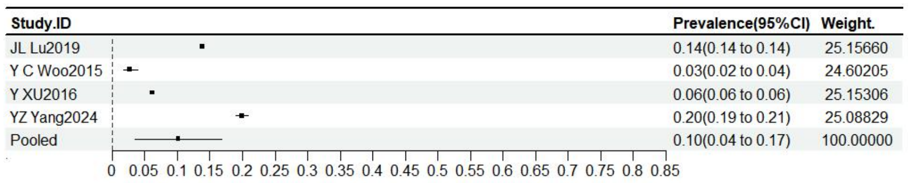

d. normal HbA1c but elevated FPG and 2hPG

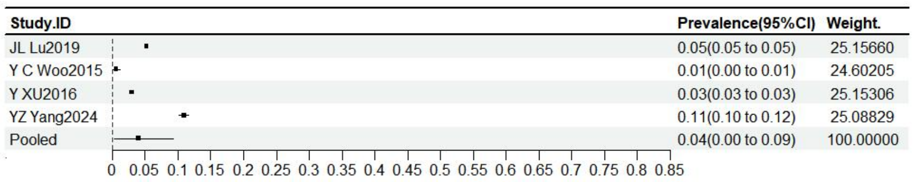

Statistics:
I-squared(95%CI): 99.917 (99.902 - 99.930)
Cochran's Q: 3634.727
Chi2, p: 0
tau2: 0.052

FPG. (a+d+e+g)

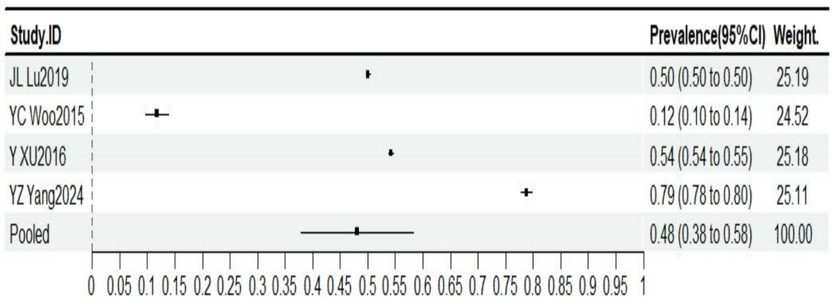

Statistics:
I-squared(95%CI): 99.901 (99.882 - 99.917)
Cochran's Q: 3030.246
Chi2, p: 0
tau2: 0.043

2hPG. (b+d+f+g)

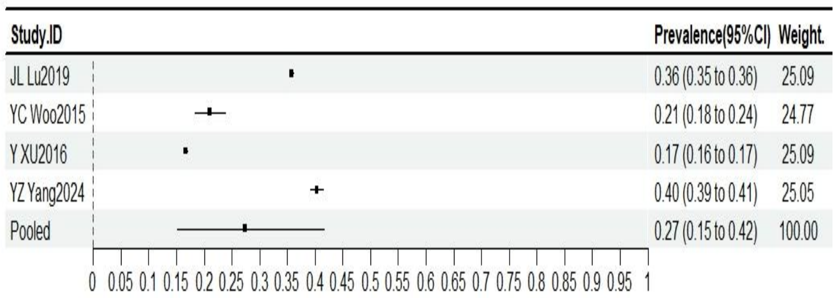

Statistics:
I-squared(95%CI): 99.952 (99.945 - 99.958)
Cochran's Q: 6225.030
Chi2, p: 0
tau2: 0.088

HbA1c. (c+e+f+g)

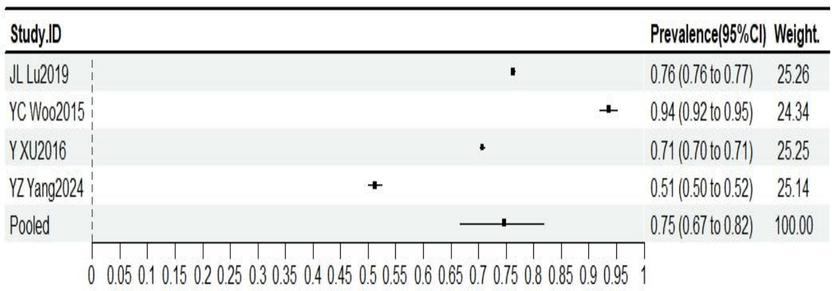

Statistics:
I-squared(95%CI): 99.862 (99.831 - 99.887)
Cochran's Q: 2177.018
Chi2, p: 0
tau2: 0.031

Supplementary Figure 6. Sensitivity analyses (retaining only studies with nationally or regionally representative samples)—Forest plot of the proportions of each combination of 2-hour post-load glucose, fasting plasma glucose, and glycated hemoglobin among general population newly diagnosed with pre-diabetes
