# Supplementary material for: Contributions of 2‐h post‐load glucose, fasting blood glucose and glycosylated haemoglobin elevations to the prevalence of diabetes and pre‐diabetes in adults: A systematic analysis of global data
Source: Diabetes Obes Metab. 2025 Sep 15;27(12):7285–98. doi: 10.1111/dom.70130 (PMC12587253; doi:10.1111/dom.70130)
Supplement: Supplementary file 20 — Figure S8. Sensitivity analyses (retaining only studies with ≥7 low‐risk items)—the proportions of different combinations of 2‐h post‐load glucose, fasting plasma glucose and glycosylated haemoglobin among general adult population newly diagnosed with pre‐diabetes. [file DOM-27-7285-s023.pdf]

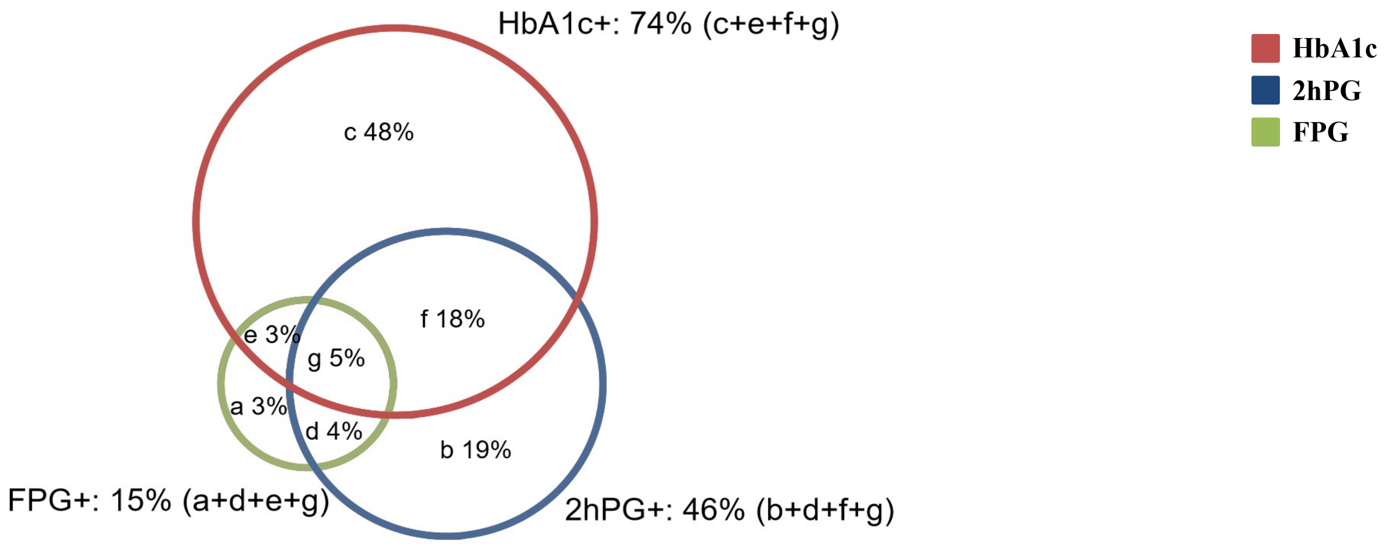

Supplementary Figure 8. Sensitivity analyses (retaining only studies with≥7 low-risk items)—The proportions of different combinations of 2-hour post-load glucose, fasting plasma glucose, and glycated hemoglobin among general adult population newly diagnosed with pre-diabetes.

Abbreviations: FPG: fasting blood glucose; 2hPG: 2-hour post-load glucose; HbA1c: glycosylated hemoglobin.
