# Supplementary material for: Contributions of 2‐h post‐load glucose, fasting blood glucose and glycosylated haemoglobin elevations to the prevalence of diabetes and pre‐diabetes in adults: A systematic analysis of global data
Source: Diabetes Obes Metab. 2025 Sep 15;27(12):7285–98. doi: 10.1111/dom.70130 (PMC12587253; doi:10.1111/dom.70130)
Supplement: Supplementary file 21 — Figure S9. Sensitivity analyses (retaining only studies with ≥7 low‐risk items)—Forest plot of the proportions of each combination of 2‐h post‐load glucose, fasting plasma glucose and glycosylated haemoglobin among adult participants newly diagnosed with diabetes. (A) The general population; (B) the population with specific diseases. [file DOM-27-7285-s012.pdf]

(A) the general population

a. normal 2hPG and HbA1c but elevated FPG (isolated FPG elevation)

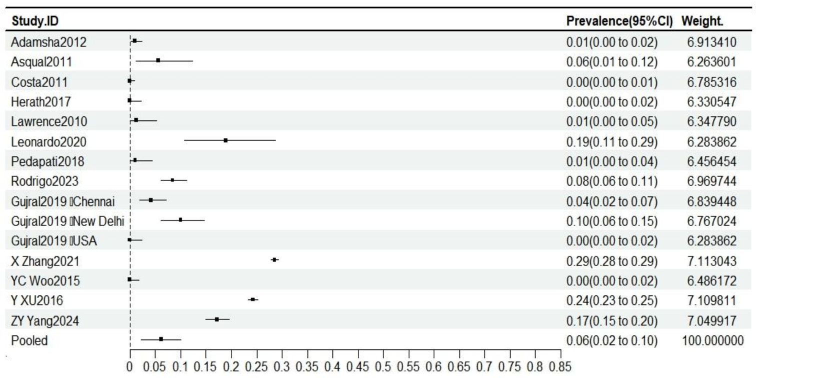

e. normal 2hPG but elevated FPG and HbA1c

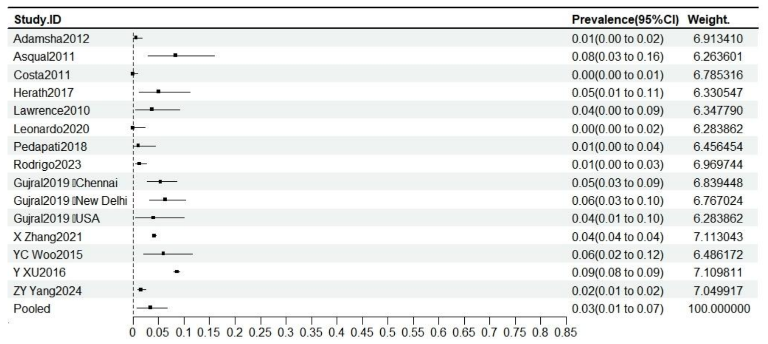

b. normal FPG and HbA1c but elevated 2hPG (isolated 2hPG elevation)

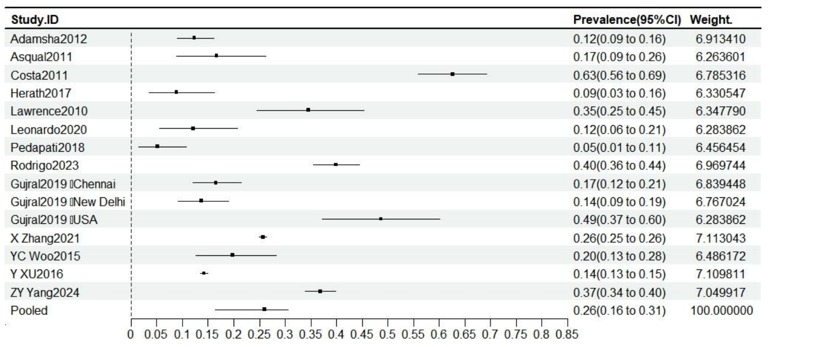

f. normal FPG but elevated 2hPG and HbA1c

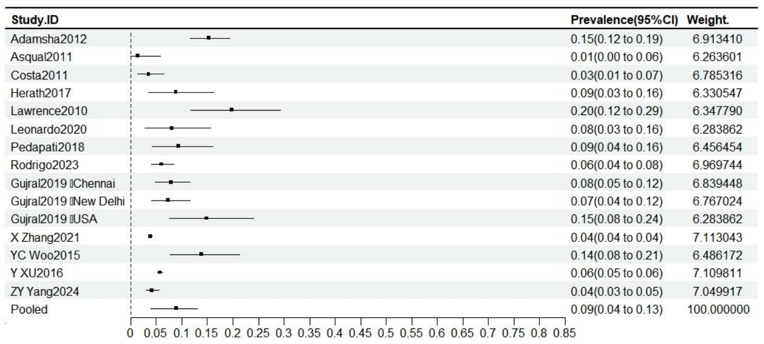

c. normal FPG and 2hPG but elevated HbA1c (isolated HbA1c elevation)

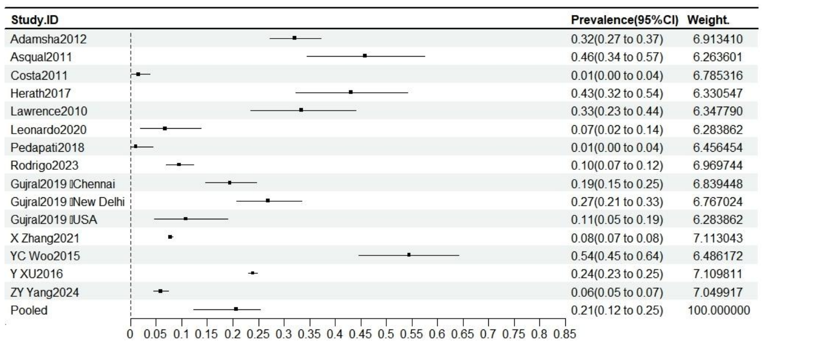

g. elevated FPG, 2hPG and HbA1c

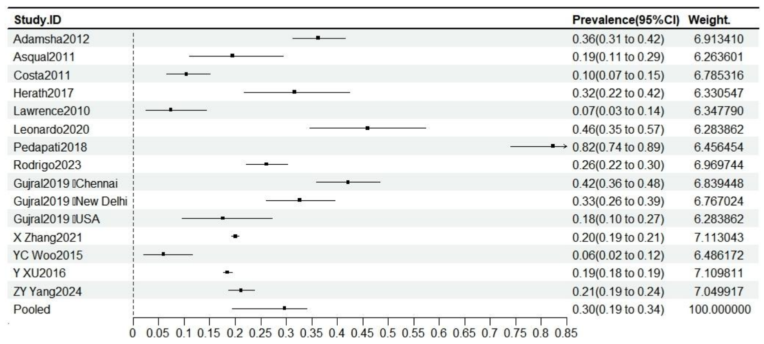

d. normal HbA1c but elevated FPG and 2hPG

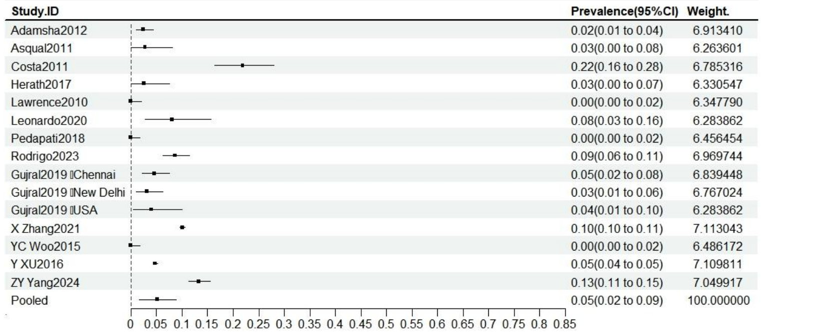

Statistics:

I-squared(95%CI): 99.036(98.838 - 99.200)

Cochran's Q: 1452.295

Chi2, p: 0

tau2: 0.101

FPG. (a+d+e+g)

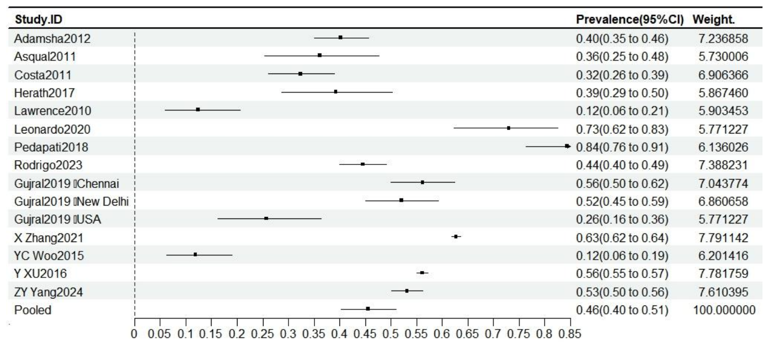

Statistics:

I-squared(95%CI): 97.480 (96.744 - 98.050)

Cochran's Q: 555.564

Chi2, p: 0

tau2: 0.038

2hPG. (b+d+f+g)

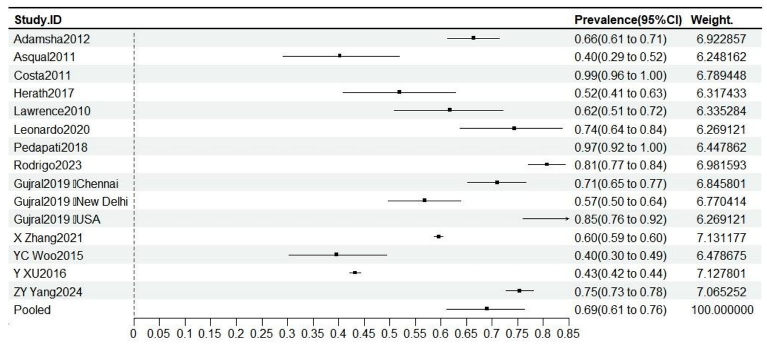

Statistics:

I-squared(95%CI): 98.996(98.786 - 99.169)

Cochran's Q: 1394.153

Chi2, p: 0

tau2: 0.097

HbA1c. (c+e+f+g)

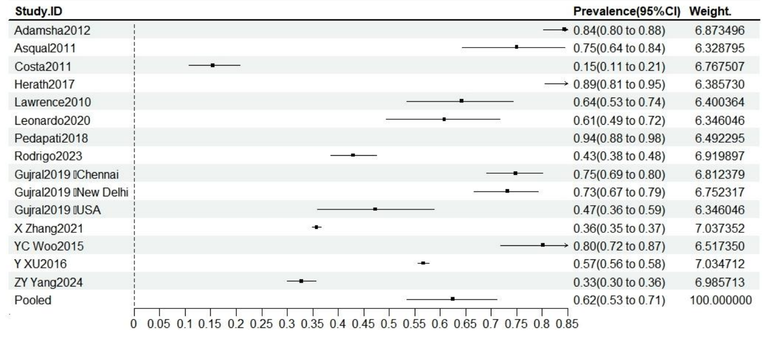

Statistics:

I-squared(95%CI): 99.203(99.050 - 99.331)

Cochran's Q: 1756.458

Chi2, p: 0

tau2: 0.122

(B) the population with specific diseases

a. normal 2hPG and HbA1c but elevated FPG (isolated FPG elevation)

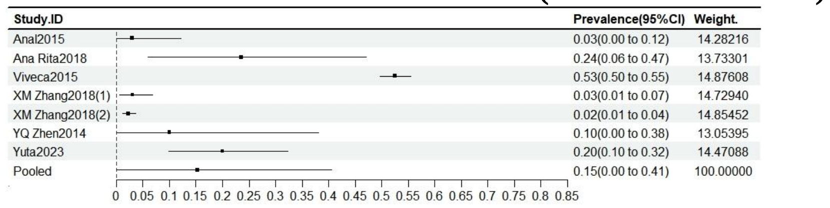

e. normal 2hPG but elevated FPG and HbA1c

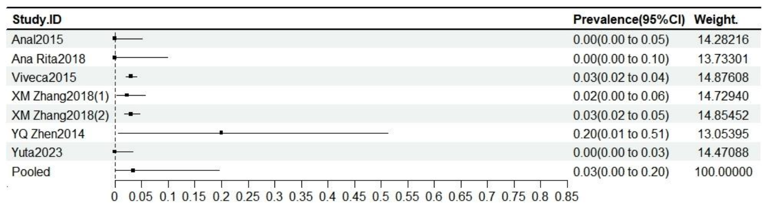

b. normal FPG and HbA1c but elevated 2hPG (isolated 2hPG elevation)

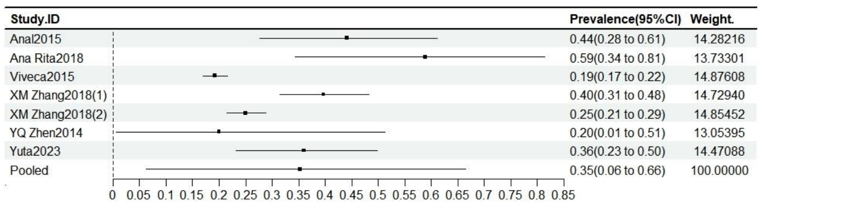

f. normal FPG but elevated 2hPG and HbA1c

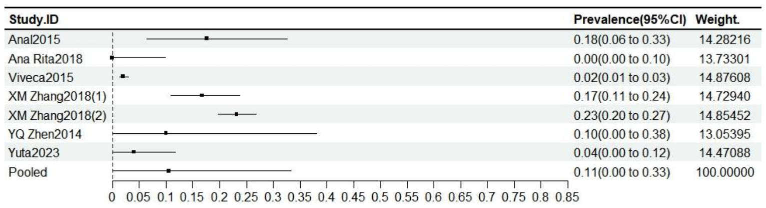

c. normal FPG and 2hPG but elevated HbA1c (isolated HbA1c elevation)

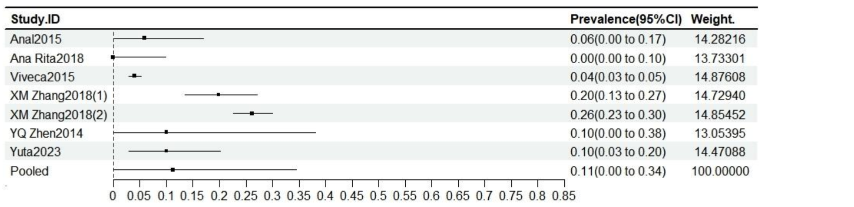

g. elevated FPG, 2hPG and HbA1c

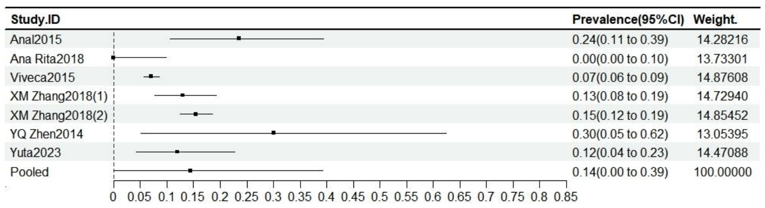

d. normal HbA1c but elevated FPG and 2hPG

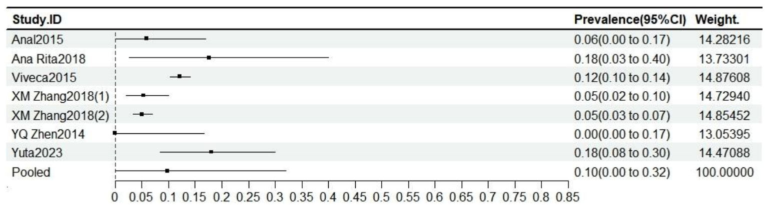

Statistics:

I-squared(95%CI): 99.190 (98.940 - 99.380)

Cochran's Q: 740.478

Chi2, p: 0

tau2: 0.675

FPG. (a+d+e+g)

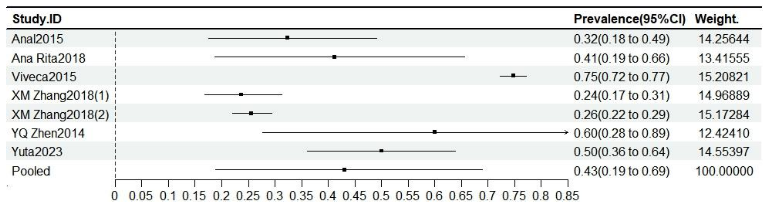

Statistics:

I-squared(95%CI): 98.705 (98.226 - 99.054)

Cochran's Q: 463.147

Chi2, p: 0

tau2: 0.420

2hPG. (b+d+f+g)

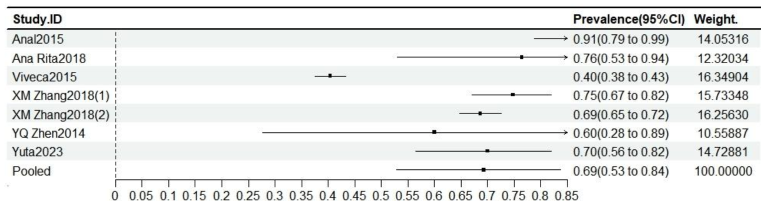

Statistics:

I-squared(95%CI): 96.879 (95.264 - 97.943)

Cochran's Q: 192.251

Chi2, p: 0

tau2: 0.171

HbA1c. (c+e+f+g)

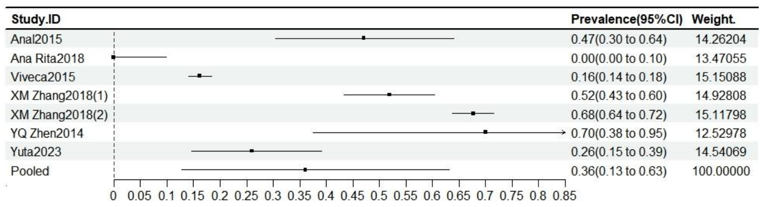

Statistics:

I-squared(95%CI): 98.790 (98.355 - 99.110)

Cochran's Q: 495.776

Chi2, p: 0

tau2: 0.450

Supplementary Figure 9. Sensitivity analyses (retaining only studies with≥7 low-risk items) —Forest plot of the proportions of each combination of 2-hour post-load glucose, fasting plasma glucose, and glycated hemoglobin among adult participants newly diagnosed with diabetes. (A) the general population; (B) the population with specific diseases
