# Supplementary material for: Contributions of 2‐h post‐load glucose, fasting blood glucose and glycosylated haemoglobin elevations to the prevalence of diabetes and pre‐diabetes in adults: A systematic analysis of global data
Source: Diabetes Obes Metab. 2025 Sep 15;27(12):7285–98. doi: 10.1111/dom.70130 (PMC12587253; doi:10.1111/dom.70130)
Supplement: Supplementary file 22 — Figure S10. Sensitivity analyses (retaining only studies with≥7 low‐risk items)—forest plot of the proportions of each combination of 2‐h post‐load glucose, fasting plasma glucose and glycosylated haemoglobin among the adult population with specific diseases newly diagnosed with pre‐diabetes. [file DOM-27-7285-s025.pdf]

the population with specific diseases

a. normal 2hPG and HbA1c but elevated FPG (isolated FPG elevation)

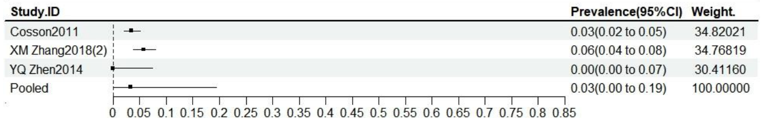

e. normal 2hPG but elevated FPG and HbA1c

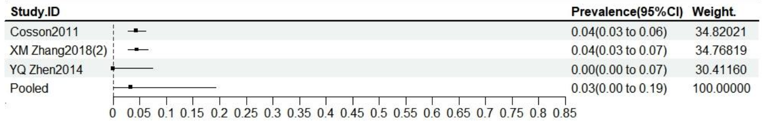

b. normal FPG and HbA1c but elevated 2hPG (isolated 2hPG elevation)

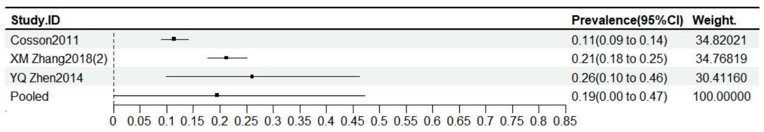

f. normal FPG but elevated 2hPG and HbA1c

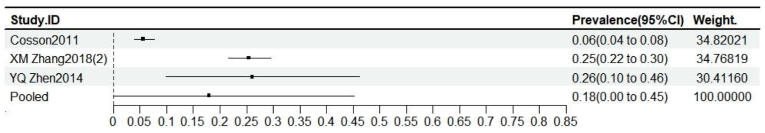

c. normal FPG and 2hPG but elevated HbA1c (isolated HbA1c elevation)

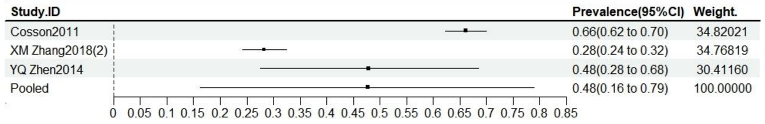

g. elevated FPG, 2hPG and HbA1c

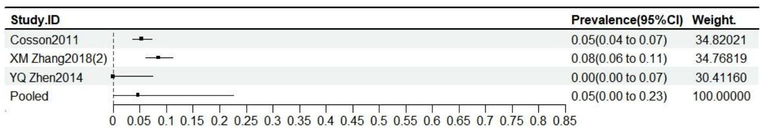

d. normal HbA1c but elevated FPG and 2hPG

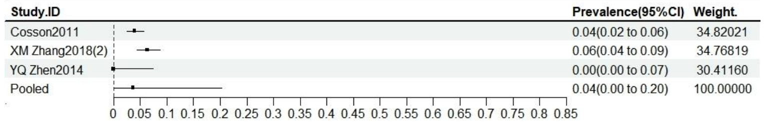

Statistics:

I-squared(95%CI): 98.737 (97.847 - 99.259)

Cochran's Q: 158.354

Chi2, p: 0

tau2: 0.280

FPG. (a+d+e+g)

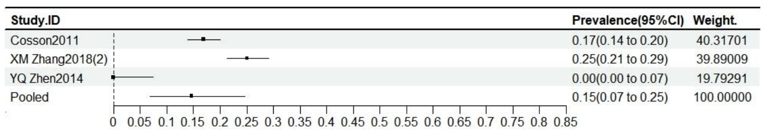

Statistics:

I-squared(95%CI): 91.320 (77.616 - 96.634)

Cochran's Q: 23.043

Chi2, p: 0

tau2: 0.038

2hPG. (b+d+f+g)

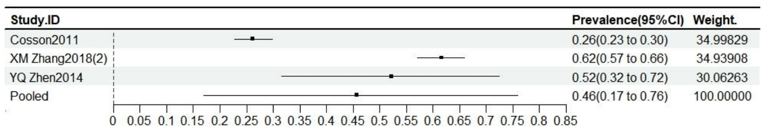

Statistics:

I-squared(95%CI): 98.571 (97.510 - 99.180)

Cochran's Q: 139.945

Chi2, p: 0

tau2: 0.247

HbA1c. (c+e+f+g)

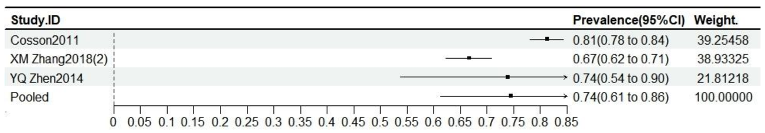

Statistics:

I-squared(95%CI): 93.236 (83.593 - 97.211)

Cochran's Q: 29.568

Chi2, p: 0

tau2: 0.049

Supplementary Figure 10. Sensitivity analyses (retaining only studies with≥7 low-risk items)—Forest plot of the proportions of each combination of 2-hour post-load glucose, fasting plasma glucose, and glycated hemoglobin among the adult population with specific diseases newly diagnosed with pre-diabetes
