# Supplementary material for: Contributions of 2‐h post‐load glucose, fasting blood glucose and glycosylated haemoglobin elevations to the prevalence of diabetes and pre‐diabetes in adults: A systematic analysis of global data
Source: Diabetes Obes Metab. 2025 Sep 15;27(12):7285–98. doi: 10.1111/dom.70130 (PMC12587253; doi:10.1111/dom.70130)
Supplement: Supplementary file 25 — Figure S13. Funnel plot for the meta‐analysis of contribution of fasting plasma glucose diagnosis elevation to prevalence of diabetes in general population. [file DOM-27-7285-s003.pdf]

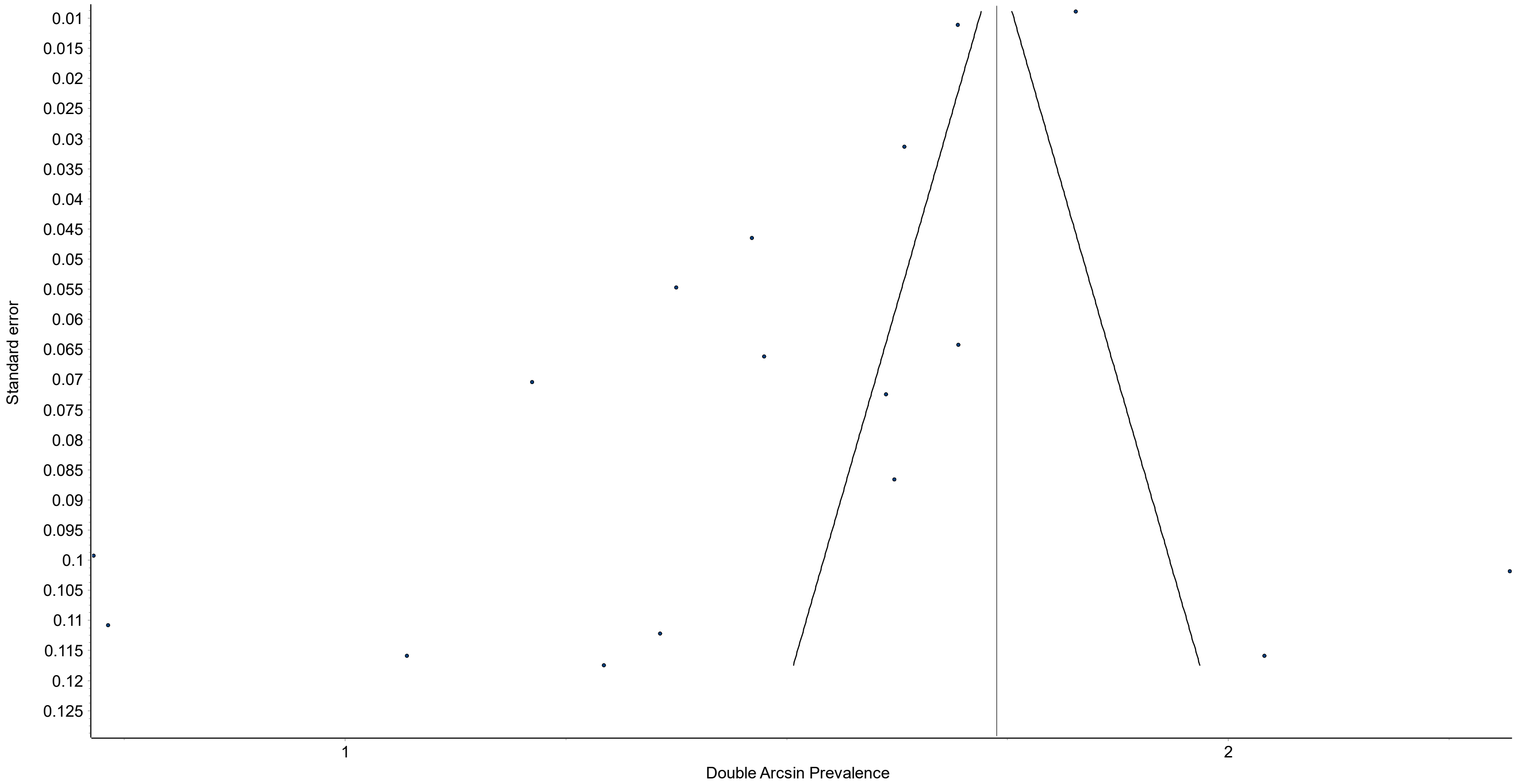

Supplementary Figure 13. Funnel plot for the meta-analysis of contribution of fasting plasma glucose diagnosis elevation to prevalence of diabetes in general population
